# Plant phenomics: an overview of image acquisition technologies and image data analysis algorithms

**DOI:** 10.1093/gigascience/gix092

**Published:** 2017-10-03

**Authors:** Fernando Perez-Sanz, Pedro J Navarro, Marcos Egea-Cortines

**Affiliations:** 1 Genetics, ETSIA, Instituto de Biotecnología Vegetal, Universidad Politécnica de Cartagena, 30202 Cartagena, Spain; 2 Genetics, Instituto de Biotecnología Vegetal, Universidad Politécnica de Cartagena, Campus Muralla del Mar, s/n, Cartagena 30202, Spain

**Keywords:** Algorithms, artificial vision, deep learning, hyperspectral cameras, machine learning, segmentation

## Abstract

The study of phenomes or phenomics has been a central part of biology. The field of automatic phenotype acquisition technologies based on images has seen an important advance in the last years. As with other high-throughput technologies, it addresses a common set of problems, including data acquisition and analysis. In this review, we give an overview of the main systems developed to acquire images. We give an in-depth analysis of image processing with its major issues and the algorithms that are being used or emerging as useful to obtain data out of images in an automatic fashion.

## Background

The development of systems to monitor large fields using the Normalized Difference Vegetation Index (NDVI) started more than 25 years ago when NDVI was used in the so-called remote sensing field [1]. It was an important milestone in the advance of automatic methods for analysing plant growth and biomass [2]. Ever since, new technologies have increased our capacity to obtain data from biological systems. The ability to measure chlorophyll status from satellite images allowed plant health to be measured in large fields and predict crops and productivity in very large areas such as the Canadian prairies, Burkina Faso, or the Indian Basin in Pakistan [3–6]. Thus, the field of remote sensing is an important basis where knowledge about data acquisition and analysis started. The development of phenotyping devices using local cameras for crops took off using an array of technologies including Infrared thermography to measure stomatal opening or osmotic stress [7–9]. Extraction of quantitative data from images has been developed to study root development [10–12] and has found a niche to identify germplasm resistant to abiotic stresses in plants such as cereals [13], Arabidopsis [14], and large-scale field phenotyping [15]. There are several recent reviews addressing the different types of growing setups [16–22], and we will not cover them in the current review.

**Figure 1:**
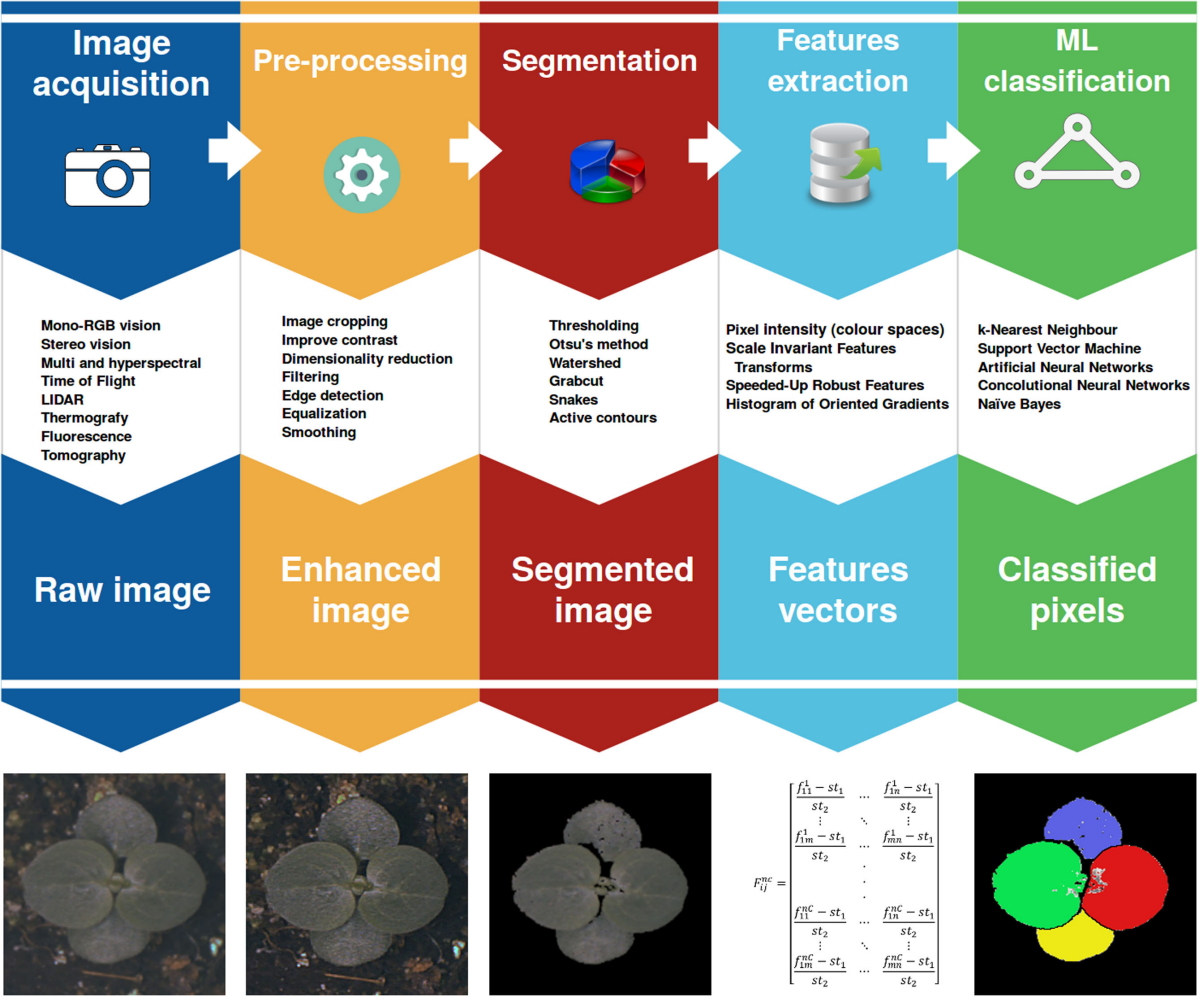
Basic workflow in computer vision–based plant phenotyping.

Two main aspects to consider are the type of image acquired and how to process it. There are a number of recent reviews on phenomics and high-throughput image data acquisition [15, 23–26]. In contrast, the majority of the literature concerning image processing and analysis is found in books where methods are described in detail [27–31]. There are some very good reviews on aspects of data acquisition and analysis, i.e., imaging techniques [32], machine learning (ML) for high-throughput phenotyping [33], or software for image analysis [34], but a detailed review on the different types of data analysis is lacking. In this review, we cover the current and emerging methods of image acquisition and processing that allow image-based phenomics (Fig. 1).

## Review

### Image acquisition

Image acquisition is the process through which we obtain a digital representation of a scene. This representation is known as an image, and its elements are called pixels (picture elements). The electronic device used to capture a scene is known as an imaging sensor. A charge-coupled device (CCD) and complementary metal oxide semiconductor (CMOS) are the most broadly used technologies in image sensors. A light wavelength is captured by small analogic sensors, which will acquire major or minor charge depending on the amount of incident light. These signals are amplified, filtered, transported, and enhanced by means of specific hardware. A suitable output interface and a lens in the same housing are all that is needed to perform image acquisition. The elements enumerated above comprise the main element of computer vision systems, the camera. Time delay and integration (TDI) is an imaging acquisition mode that can be implemented over CCD [35] or CMOS [36]. It improves the features of the image acquisition system considerably. TDI is used in applications that require the ability to operate in extreme lighting conditions, requiring both high speed and high sensitivity, e.g., inline monitoring, inspection, sorting, and remote sensing (for weather or vegetation observation) [36].

The aforementioned technologies, CCD, CMOS, and TDI, confer unique characteristics, which define the type of data a camera can provide with a degree of robustness. There are fundamental differences in the types of performance the different sensors offer. In recent years, CMOS technology has outperformed CCDs in most visible imaging applications. When selecting an imaging sensor (a camera), CCD technology causes less noise and produces higher-quality images, mainly in scenes with bad illumination. It has a better depth of colour due to the higher dynamic range. On the other hand, the CMOS sensors are faster at processing images. Due to the hardware architecture for pixel extraction, they need less electrical power to operate, they allow a region of interest to be processed on the device, and they are cheaper than CCDs. Furthermore, TDI mode with CCD or CMOS imaging sensors is used for high-speed and low–light level applications [37]. The latest technological developments in cameras show that the trend of the manufacturers such as IMEC, world-leader in nanoelectronics, is to fuse TDI technology with CCD and CMOS characteristics in the same device [38]. TDI technology is expected to be applied to high-throughput phenotyping processes in the near future.

The field of image acquisition is extremely developed in the literature, but image acquisition systems can be classified into 7 groups that are suitable for phenotyping.

#### Mono-RGB vision

Mono-RGB vision systems are composed of a set comprising a lens, imaging sensor, specific hardware, and input/output (IO) interface. Depending on if they use a line or matrix of pixels, they are classified as line cameras (or scanners) or matrix cameras. Most computer vision phenotyping devices are based on mono-RGB vision systems. Examples of mono-RGB vision devices include Smart tools for the Prediction and Improvement of Crop Yield, an automated phenotyping prototype of large pepper plants in the greenhouse. The system uses multiple RGB cameras to extract 2 types of features: features from a 3D reconstruction of the plant canopy and statistical features derived directly from RGB images [39]. A different approach has been used with 2 cameras inside a growth chamber to measure circadian growth features of *Petunia, Antirrhinum*, and *Opuntia* [40]. Two cameras with low and high magnifications were used to carry out phenotype studies of *Arabidopsis thaliana* seeds. The system is mounted on a 3-axis gantry, and the rotation of the samples allows the gravitropic bending response to be determined in the roots, as well as its posterior quantification [41]. Recently a high-throughput RGB system has been developed to identify quantitative trait loci (QTL) involved in yield in large recombinant inbred lines in maize [42], demonstrating the increasing impact of this approach in phenomics.

These devices have excellent spatial and temporal resolution; i.e., they can produce a very large number of images in very short periods and at a very low cost. They are portable, and there are many software tools to perform image processing (Table 1). Systems based on mono-RGB vision allow a quantification of the plant canopy [43], as well as sufficient computation of vegetation indices for most purposes. The main disadvantages are caused by the overlap of plant organs during growth and nutation phases and the relative position of the organs with respect to the device that makes the precise quantification difficult. In addition, these devices are affected by variations in illumination when used outdoors. The trend in outdoor plant phenotyping is to combine mono-RGB systems with other systems such as light detection and ranging (LIDAR) devices (see below) or thermal imaging, or adding new bands or filters to the camera that allow the segmenting of specific regions of the spectrum [44, 45].

**Table 1:** List of software tools for image processing

Vision libraries	Source	Language
OpenCV	http://opencv.org	C++, Python, Java, C#
EmguCV	http://www.emgu.com/	
PlantCV	http://plantcv.danforthcenter.org	Python
Scikit-image	http://scikit-image.org	
Bioimagetools, bayesimages, edci, DRIP, dpmixsim, raster, …	https://cran.r-project.org/	R
Cimg	http://cimg.eu	C++
Simplecv	http://simplecv.org	
Fastcv	https://developer.qualcomm.com/software/fastcv-sdk	
Ccv	http://libccv.org	
Vxl	http://vxl.sourceforge.net	
BoofCV	http://boofcv.org	Java
OpenIMAJ	http://openimaj.org	
JavaCV	https://github.com/bytedeco/javacv	

#### Stereo vision

Stereo vision systems try to correct a drawback of mono-RGB vision systems for distance measurement. The architecture of stereo vision systems emulates the behaviour of human vision using 2 mono vision systems. Basically, after locating a point in 2 mono vision systems, it is possible to compute the distance from the point to the system. Images produced are known as depth maps [46]. A stereo vision system has been used by Biskup and colleagues [47] to obtain structural features of plant canopies. The 3D reconstruction has been successfully employed to obtain 3D models of plants, thus demonstrating the power of this approach [48]. Simple depth reconstructions help to define stems, leaves, and grapes, showing the potential of this technology [49]. An RGB camera mounted on a mobile robot is used as an automated 3D phenotyping of vineyards under field conditions. Sequentially, the system captures a set of images, which are used to reconstruct a textured 3D point cloud of the whole grapevine row [50]. Stereo vision has been developed to perform high-throughput analysis of rapeseed leaf traits. The system uses 2 identical RGB cameras to obtain stereo images for canopy and 3D reconstruction [51]. Developing a 3D-mesh segmentation has allowed cotton growth to be analysed [52], showing the further possibilities of 3D imaging.

The main advantage of 3D systems is their simplicity; 2 cameras are enough to obtain depth maps. Stereo vision has evolved into multi-view stereo (MSV) and has found a place in plant phenotyping [53]. Furthermore, MSV is a low-cost 3D image acquisition system compared with other technologies such as LIDAR or tomography imaging [54]. Stereo vision systems have important weaknesses. They are affected by changes of the scene illumination, they need a high-performance computational system to carry out stereo-matching algorithms, and they have poor depth resolution [55]. These limitations are increased in outdoor environments, as image segmentation becomes more challenging.

#### Multi- and hyperspectral cameras

Multispectral and hyperspectral cameras have been used in numerous fields of science and in industrial applications [56–61]. The spectral resolution is the main factor that distinguishes multispectral imagery from hyperspectral imagery [62]. Multispectral cameras are devices able to capture images from a number of discrete spectral bands. The number of bands has increased in the last decade as technology has improved. Currently, the main camera manufacturers offer multispectral cameras acquiring between 3 and 25 bands, including visible RGB channels, near infrared (NIR), or a set of custom bands, with a tendency to provide increasing number of bands [63]. The spectral bands may not be continuous; thus for 1 pixel we obtain a vector of information comprising the number of elements corresponding to the number of bands registered. Hyperspectral systems may reach resolutions of a few nanometers in wavelength, obtaining for each pixel a digital signature that may contain several hundreds of continuous bands within a specific range of wavelengths [64]. Traditionally, both multispectral and hyperspectral imaging have been used for remote sensing and have an increased number of applications in phenomics. A multispectral system has been developed to improve the original colour of images for fruit recognition [65]. The authors fused the original colour image with an infrared image using nonlinear Daubechies wavelet transform (DWT). Thus, the additional information from the second image allows the original to be improved.

**Figure 2:**
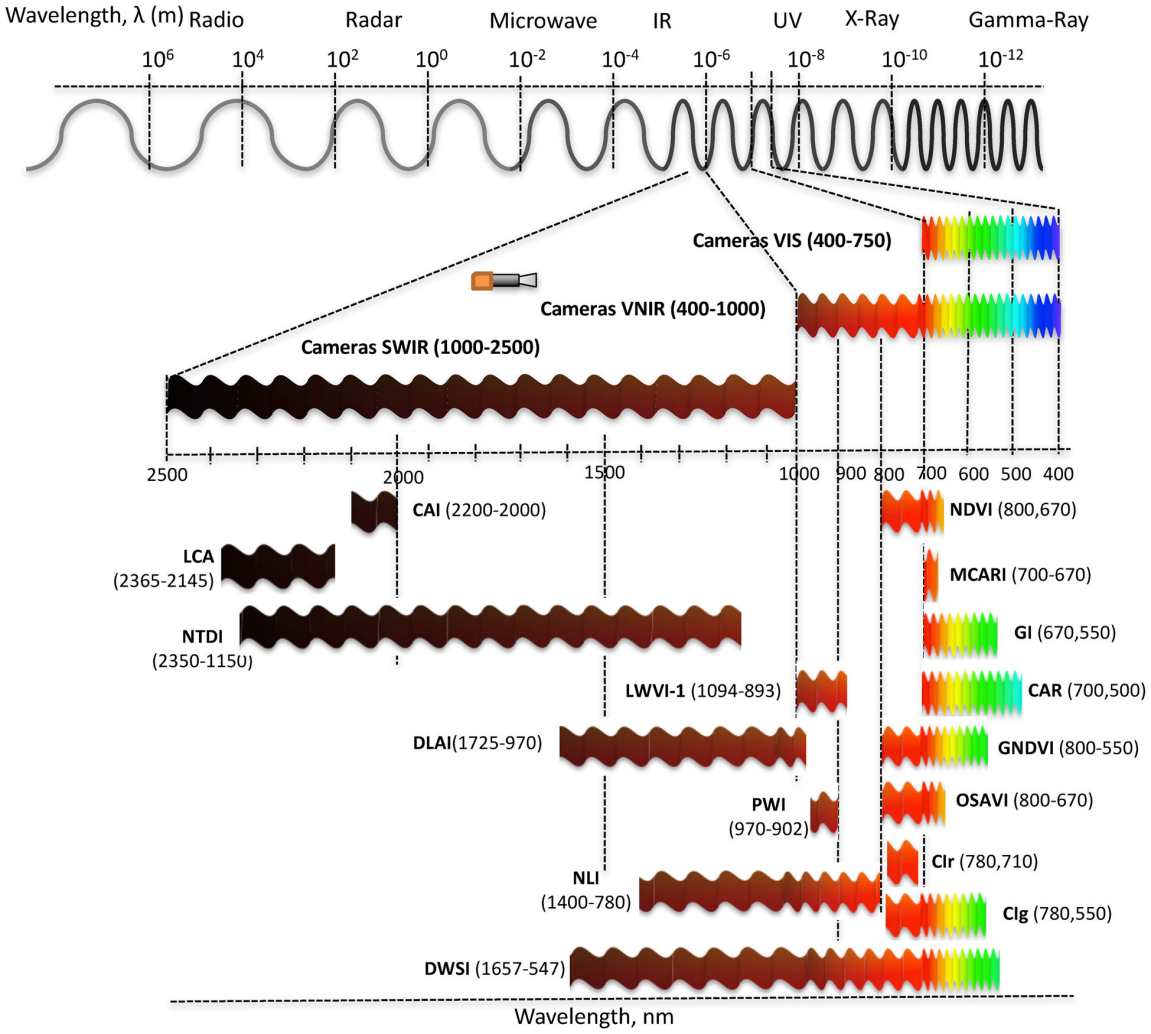
An overview of different spectra used for phenotyping and the associated cameras. The names of different indexes are found in Table 2.

The use of hyperspectral cameras is increasing in phenotyping experiments as they allow the identification of physiological responses, pathologies, or pests in a noninvasive way. Using hyperspectral images, a system has been developed to identify pathogens in barley leaves using probabilistic topic models [66]. A hyperspectral microscope was used to determine spectral changes on the leaf and cellular level of barley (*Hordeum vulgare*) during resistance reactions against powdery mildew (*Blumeria graminis f.sp. hordei, isolate K1*) [67]. A detailed description of the different wavelengths and combinations used in multispectral and hyperspectral cameras can be seen in Fig. 2, and their uses in Table 2. We expect to see an increase in phenomic setups using multispectral and hyperspectral cameras in the future. An emerging issue will be the data analysis as the number of pictures doubles with each additional spectrum used for analysis (see below).

**Table 2:** A list of indexes, the corresponding wavelength ranges, and their use to analyse plant material

Index	Range, nm	Applications
CAI—Cellulose Absorption Index	2200–2000	Quantification of mixed soil–plant litter scenes [178], estimation of non-photosynthetic biomass [179]
LCA—Lignin-Cellulose Absorption Index	2365–2145	Measure of the effects of soil composition and mineralogy of crop residue cover [180]
NTDI—Normalized Difference Tillage Index	2359–1150	Used for identifying crop residue cover in conventional and conservation tillage systems [181]
LWVI-1 – Normalized Difference Leaf water VI 2	1094–893	Discrimination of sugarcane varieties, allowed to detect large amounts of non-photosynthetically active constituents within the canopy [182]
DLAI—Difference Leaf Area Index	1725–970	Used for estimating leaf area index based on the radiation measurements in the visible and near-infrared [183]
PWI—Plant Water Index	970–902	Water content estimation and study of the characteristics of canopy spectrum and growth status [184, 185]
NLI—Nonlinear Vegetation Index	1400–780	Measurement of plant leaf water content; in combination with others, indexes can detect interaction of biochemicals such as protein, nitrogen, lignin, cellulose, sugar, and starch [186]
DWSI—Disease Water Stress Index	1657–547	To predict larval mosquito presence in wetland [187] and detect sugarcane “orange rust” disease [188]
NDVI—Normalized Difference Vegetation Index	800–670	Measurement of significant variations in photosynthetic activity and growing season length at different latitudes [189]
MCARI—Modified Chlorophyll Absorption Ratio Index	700–670	Study of vegetation biophysical parameters, as well as external factors affecting canopy reflectance [190]
GI—Greenness Index	670–550	Characterization of corn nitrogen status [191]
CAR—Chlorophyll Absorption Ratio	700–500	Estimating the concentration of individual photosynthetic pigments within vegetation [192]
GNDVI—Green Normalized Difference Vegetation Index	800–550	Providing important information for site-specific agricultural decision-making [193] and for identification of chlorophyll content and tissue nitrogen [194]
OSAVI—Optimized Soil Adjusted Vegetation Index	800–670	Measurement of highly sensitive chlorophyll content variations that are very resistant to the variations of LAI and solar zenith angle [195]
CI r—Coloration Index red	780–710	Mapping of coastal dune and salt marsh ecosystems [196]
CI g—Coloration Index green	780–550	Characterization of the state of soil degradation by erosion [197]

#### ToF cameras

The Time of Flight cameras (ToF cameras) have been one of the last imaging devices to be incorporated into automatic plant phenotyping [68]. ToF has as a general principle the measurement of the distance between the objective of the camera and each pixel. This is achieved by measuring the time it takes for a signal emitted in NIR to come back, reflected by the object. This allows a precision 3D reconstruction. Stereo vision coupled with ToF images has been implemented to increase the performance of methods of image segmentation to obtain leaf areas [69]. Beyond the tedious hand work required for manual analysis, sampling is done in a non-destructive way. Depth maps obtained by a ToF camera, together with colour images, are used to carry out the 3D modelling of leaves. The system is mounted on a robotic arm, which allows image acquisition to be automated [70]. A ToF has been successfully used to identify QTL regulating shoot architectures of *Sorghum* by means of 3D reconstruction [71].

Microsoft Kinect is a low-cost image acquisition system designed for video gaming that can be used for characterization and for tracking of phenological parameters [72]. The device is composed of an infrared projector and a camera that generates a grid from which the location of a nearby object in 3D can be ascertained [73]. Kinect has been used to measure plant structure and size for 2 species growing in California grasslands [74]. The quantitative 3D measurements of the architecture of the shoot and structure of the leaves can be performed when proper segmentation algorithms are developed, suggesting some potential for ToF systems [75].

The main disadvantages of this acquisition system are the low resolution, a reduced distance range of a few meters, and the high dependence on the reflecting surface for imaging. As a result, it cannot operate under strong sunlight and is more appropriate for indoor conditions. Its reduced cost and the possibility of obtaining 3D structures of entire plants, as well as of individual organs, make this system very attractive for indoor phenotyping.

#### LIDAR technology

LIDAR is a remote sensing technology developed at the beginning of the 70s to monitor the Earth's surface [76]. LIDAR uses a laser pulse light to measure the distance between the light source and the object by calculating the time of emission and the time of reflected light detection. It allows the creation of a cloud of points that reconstruct the 3D structure of an object [77, 78]. LIDAR has been used in image acquisition from distances of thousands of kilometres to centimetres, demonstrating the great potential of these types of devices. Satellite-based LIDAR systems are used for the measurements of vegetation canopy height, area, volume or biomass, etc. [79–81]. Recent development using both manned and unmanned flights has allowed the estimation of biomass dynamics of a coniferous forest using Landsat satellite images, together with ground and airborne LIDAR measurements [82]. Terrestrial LIDAR sensors are applied to detect and discriminate maize plants and weeds from soil surface [83]. Short-range LIDAR can be deployed for high-throughput phenotyping systems for cotton plant phenotyping in the field [84] or tomato leaf area by 3D laser reconstruction [85]. Fully automated crop monitoring is feasible using centimetre ranges from robotized or gantry systems [43]. An autonomous robotic system has allowed 3D mapping of plant structures to be performed with millimetric precision [86]. A LASER SCAN mounted on an XYZ gantry system was used to estimate the growth measures and structural information of plants through laser triangulation techniques [87]. Thus, using different devices, LIDAR has an impressive range of possibilities for plant phenomics.

Some shortcomings of LIDAR devices for plant phenotyping are the absence of colour in the measurement, excessive time to compute the cloud points, low precision for massive phenotyping, scanning noises caused by wind, rain, insects, and small particles in the air, and the requirement of calibration. Recent advantages suggest that the use of LIDAR technologies could overcome some of the challenges for the next-generation phenotyping technologies [88]. Developments in multispectral LIDAR instruments show novel systems that are capable of measuring multiple wavelengths and obtaining vegetation indexes (see below) [89, 90] or measuring arboreal parameters [91]. The massive adoption of LASER technologies by autonomous car manufactures has fostered the development of 3D high-definition LIDAR (HDL) with real-time (RT) capacities. The new 3D HDLs are capable of generating 1.3 million points per second with a precision of 2 cm and distances of up to 120 meters [92]. These new devices open the door to the RT massive phenotyping in outdoor and indoor crops.

#### Thermography and fluorescence imaging

Thermography is a widely used technology in remote sensing and plant phenotyping [93–96]. Thermographic cameras are able to acquire images at wavelengths ranging from 300 to 14 000 nm [97], thus allowing the conversion of the irradiated energy into temperature values once the environmental temperature is assessed. Plants open stomata in response to environmental cues and circadian clock depending on the type of photosynthetic metabolism they have [98, 99]. The evapotranspiration can be assessed with thermography [100], and quantification can be made at different scales, such as a leaf, a tree, a field, or a complete region. Water stress and irrigation management are 2 fields of application of thermography imaging [101–104]. Thermography imaging can detect local changes of temperature produced due to pathogen infection or defence mechanisms [105]. Oerke et al. used a digital infrared thermography to correlate the maximum temperature difference (MTD) of apple leaves with all stages of scab development [106].

Fluorescence imaging has been used in a large number of experimental setups, as ultraviolet (UV) light in the range of 340–360 nm is reflected by different plant components as discrete wavelengths [32]. The corresponding wavelengths emitted are cinnamic acids in the range of green-blue (440–520 nm). Early experiments using reflected fluorescence allowed the identification of phenylpropanoid synthesis mutants in Arabidopsis [107]. Chlorophyll fluorescence emits in red and far-red (690–740 nm). It is an important parameter that has been studied as a proxy for different biological processes such as circadian clock or plant health [8, 108, 109]. A system based on a UV light lamp and a conventional camera with a UV filter to avoid RGB and infrared (IR) images has been used to identify changes in UV absorbance related to pollination [110]. Multicolour fluorescence detection uses the combination of chlorophyll and secondary metabolite–emitted fluorescence to determine plant health in leaf tissues [111].

Thermography imaging results in an estimable tool for the monitoring of genotypes and detection of plant diseases [112] where all the specimens are located under strict control conditions: Temperature, wind velocity, irradiance, leaf angle, and canopy leaf structures are potential issues for quality image acquisition. The next generation of thermography imaging for phenotyping will have to resolve drawbacks related to temporal variations of environment conditions, aspects relating to angles of view, distance, sensitivity, and the reproducibility of the measurements [104]. Both thermographic and fluorescent images capture a single component, and images are in principle easy to analyse as segmentation based on thresholds can be applied to the acquired images. Combining thermographic and fluorescent imaging requires sophisticated data analysis methods based on neural networks to obtain quality data, but it is an emerging solution [111].

#### Tomography imaging

Magnetic resonance imaging (MRI) is a non-invasive imaging technique that uses radio frequency (RF) magnetic fields to construct tomographic images [113]. Commonly, MRI has been used to investigate the anatomical structure of the body (especially the brain) in both health and disease [114]. In plant phenomics, MRI is used to visualize internal structures and metabolites. This method poses a great potential to monitor physiological processes occurring *in vivo* [115]. MRI has allowed the development of root systems over time in the bean to be mapped [116], moisture distribution to be visualized during development in rice [117], and water presence to be analysed during the maturity process of barley grains [118].

Positron emission tomography (PET) is a nuclear medicine imaging modality that allows the assessment of biochemical processes *in vivo*, to diagnose and stage diseases and monitor their treatment [119]. Karve et al. [120] presented a study about C-allocation (carbon allocation from CO2 through photosysthesis) in large grasses such as *Sorghum bicolor.* The study concluded that the commercial PET scanners can be used reliably, not only to measure C-allocation in plants but also to study dynamics in photoassimilate transport.

X-ray computed tomography (x-ray CT) employs x-rays to produce tomographic images of specific areas of the scanned object. The process of attenuation of rays together with a rotation and axial movement over objects produces 3D images [32]. A high-throughput phenotyping system based on x-ray CT is 10 times more efficient than human operators, being capable of detecting a single tiller mutant among thousands of rice plants [121]. The remarkable penetration of x-rays has made this technology a great ally of phenotyping carried out below ground. The study of root systems and their quantification has been a field of habitual application of x-ray CT [122–126]. New developments address the reduction of penetrability and the increase of the image resolution of x-ray CT in plant tissue using phosphotungstate as a contrast agent, due to its capacity of increasing the contrast and penetrability of thick samples [127].

MRI, PET, and x-ray imaging techniques are available for screening 3D objects. MRI and PET are 2 non-destructive and non-invasive scanning technologies that have been applied in plant sciences to acquire 3D structural information [128]. MRI and PET data acquisition is time consuming, and software tools need to be further developed to analyse data and obtain physiologically interpretable results [97]. High-resolution x-ray computed tomography (HRXCT) promises to be the broadest non-destructive imaging method used in plant sciences. HRXCT will provide 3D data at a resolution suited for detailed analysis of morphological traits of *in vivo* plant samples and at a cellular resolution for *ex vivo* samples [128]. In terms of the development of devices, the trend will be to increase the resolution of images, the size of the fields of view, and increase the devices’ portability [129].

### Image analysis

Extracting information from images is performed through the process of segmentation. The aim of a segmentation procedure is to extract the components of an image that are of interest, i.e., object or region of interest from the rest of the image, i.e., background of the image or irrelevant components. Thus, we end up with a partitioned image with significant regions. The significant regions may be defined as foreground vs background or by selecting a number of individual components from an image. The construction of the selected regions is based on the image characteristics such as colour (colour spaces), spectral radiance (vegetation indexes), edge detection, neighbour similarity [130], or combinations that are integrated via a machine learning process [131]. In some cases, preprocessing is required in order to obtain a meaningful segmentation.

#### Image preprocessing

Image preprocessing is an important aspect of image analysis. The aim of image preprocessing is to improve contrast and eliminate noise in order to enhance the objects of interest in a given image [132]. This process can be extremely helpful to enhance the feature extraction quality and the downstream image analysis [133]. Preprocessing can include simple operations such as image cropping, contrast improvement, or other significantly more complex operations such as dimensionality reduction via principal component analysis or clustering [33]. One preprocessing pipeline has been proposed for plant phenotyping based on converting the image to grayscale, application of a median filter, binarization, and edge detection [134]. A similar preprocessing method has been developed to identify plant species under varying illumination conditions [135]. It comprises conversion to grayscale, image binarization, smoothing, and application of a filter to detect edges. In a comparative study to analyze leaf diseases, histogram equalization was found to be the best way to obtain preprocessing of color images converted to grayscale [136]. However, RGB images have been found to perform better than grayscale conversions when identifying leaf pathogens [137].

We cannot conclude that a single preprocessing method will outperform other methods. The quality and type of image are fundamental to selecting a type of preprocessing procedure. Nevertheless, preprocessing is a basic step that can improve image analysis, and sometimes make it possible. It should be described in the materials and methods of image procedures to make data comply to the new standards—Findability, Accessibility, Interoperability, and Reusability (FAIR) [138].

#### Image segmentation

As we mentioned above, image segmentation is the core of image processing for artificial vision-based plant phenotyping. Segmentation allows the isolation and identification of objects of interest from an image, and it aims to discriminate background or irrelevant objects [139]. The objects of interest are defined by the internal similarity of pixels in parameters such as texture, colour, statistic [133], etc. (See a list of Open software libraries for image segmentation in Table 1.)

One of the simplest algorithms used is threshold segmentation, based on creating groups of pixels on a grayscale according to the level of intensity, thus separating the background from targets. Such an approach has been used with Android OS (ApLeaf) in order to identify plant leaves [140].

The Otsu's method [141] is a segmentation algorithm that searches for a threshold that minimizes the weighted within-class variance [132]. This method has been used for background subtraction in a system that records and performs automatic plant recognition [142] and can give high-contrast segmented images in an automatic fashion [143]. Under certain circumstances, it can underestimate the signal, causing under segmentation, and is significantly slower than other thresholding methods [132].

The Watershed [144] transformation is a popular algorithm for segmentation. It treats an image as a topological surface that is flooded, and seed regions are included, usually by the user. This generates an image with gradients of magnitudes, where crests appear in places where borders are apparent (strong edges) and causes segmentation to stop at those points [130]. It has been used to identify growth rate [145], recognition of partially occluded leaves [56], individual tree crown delineation [146], and leaf segmentation [147].

Grabcut [148] is a segmentation algorithm based on graph cut [149]. It is created on graph theory to tackle the problem of separating an object or foreground from the background. The user should mark a rectangle (bounding box) surrounding the object of interest, thus defining the outrebound of the box as background [150]. This algorithm has been tested to extract trees from a figure, but it has been successful only with very simple backgrounds [151]. More recently, Grabcut has been deployed as a segmentation algorithm in a pipeline for plant recognition with multimodal information, i.e., leaf contour, flower contour, etc. [152]. Grabcut loses precision or even fails when pictures have complex backgrounds but is highly precise with simple backgrounds [151, 142].

Snakes are a special type of active contour [153] and are used as methods to fit lines (splines) either to open or close edges and lines in an image. These methods have been used for face recognition, iris segmentation, and medical image analysis. Within the field of plant phenotyping, there are procedures where active contours are used inside a protocol constructing a vector of features with data of colour intensity, local texture, and a previous knowledge of the plant incorporated via Gaussian mixture models, previously segmented [154]. These steps give an initial rough segmentation, upon which active contours can operate with a much higher precision.

Active contours have used images of flowers for plant recognition [155], based on a combination of the algorithm proposed by Yonggang and Karl [156] and the model of active contours without edges [157]. Whilst the work proposed by Minervini et al. [154] appears to give significantly better results compared with the results of Suta et al. [155], the usage of images with a natural background may be related to the apparent differences in segmentation. Thus, a current problem concerning the comparison of algorithms and procedures lies with the different backgrounds used for image acquisition.

#### Features extraction

Features extraction constitutes one of the pillars of the identification and classification of objects based on computer vision. Beyond the raw image, a feature is information that is used to resolve a specific computer vision problem. The features extracted from an image are disposed in the so-called “feature vectors.” The construction of feature vectors uses a wide set of methods to identify the objects in an image. The main features are edges, intensity of image pixels [39], geometries [158], textures [154, 159], image transformations, e.g., Fourier [160] or Wavelet [65, 161] or combinations of pixels of different colour spaces [131]. The end goal of feature extraction is to feed up a set of classifiers and machine learning algorithms (see below).

One system proposed uses a feature vector composed of a combination of RGB and CIE L*a*b* colour spaces to segment the images captured during the day [131]. The night-time image segmentation computed a vector composed of statistical features over 2 decomposition levels of the wavelet transform using IR images.

Iyer-Pascuzzi et al. presented an imaging and analysis platform for automatic phenotyping to identify genes underlying root system architecture. The authors employed a set of 16 statistical, geometric, and shape features obtained from 2297 images from 118 individuals such as median and maximum number of roots, the total root length, perimeter, and depth, among others [162].

There are a number of algorithms to identify invariant feature detectors and descriptors. This type of image analysis ensures the detection of points of interest in a scale- and rotation-independent manner. This is crucial for camera calibration and for matching to produce a set of corresponding image points in 3D image reconstruction. Furthermore, it allows the identification of points of interest even when they change scale and/or position or situations of uncontrolled illumination, a common issue when phenotyping plants. The Scale Invariant Features Transforms (SIFT) [163], Speeded-Up Robust Features (SURF) [164], and the Histograms of Oriented Gradients (HoG) [165] are algorithms used to extract characteristics in computer vision, and they have been extended to plant phenotyping. Wei et al. [166] presented an image-based method that automatically detects the flowering of paddy rice. The method uses a scale-invariant feature transform descriptor, bag of visual words, and a machine learning method. The SIFT algorithm has been used to combine stereo and ToF images with automatic plant phenotyping. It can create dense depth maps to identify pepper leaf in glasshouses [69]. SIFT and SURF algorithms have been tested for detecting local invariant features for obtaining a 3D plant model from multi-view stereo images [167]. A HoG framework allows the extraction of a reliable quantity of phenotypic data of grapevine berry using a feature vector composed of colour information [168].

So far, feature extraction has been an arduous and difficult task, requiring the testing of hundreds of feature extraction algorithms and a greater number of combinations between them. This task demands expert skills in different subjects. The success in the identification does not depend on the robustness of the classification methods, but on the robustness of the data.

#### Machine learning in plant image analysis

The amount of data generated in current and future phenomic setups with high-throughput imaging technologies has brought the use of machine learning (ML) statistical approaches. Machine learning is applied in many fields of research [169–171]. As phenotyping can generate terabytes of information, ML tools provide a good framework for data analysis. A list of ML libraries can be found in Table 3. A major advantage of ML is the possibility of exploring large datasets to identify patterns using combinations of factors instead of performing independent analysis [33].

**Table 3:** List of machine learning software libraries and their languages

Libraries ML/DL	Source	Language
MICE, rpart, Party, CARET, randomForest, nnet, e1071, KernLab, igraph, glmnet, ROCR, tree, Rweka, earth, klaR,	https://cran.r-project.org/	R
Scikit-learn	http://scikit-learn.org/stable/	Python
Tensorflow	https://www.tensorflow.org/	
Theano	http://deeplearning.net/software/theano	
Pylearn2,	http://deeplearning.net/software/pylearn2	
NuPIC	http://numenta.org/	
Caffe	http://caffe.berkeleyvision.org/	
PyBrain	http://pybrain.org/	
Weka	http://www.cs.waikato.ac.nz/ml/weka/	Java
Spark	http://spark.apache.org/	
Mallet	http://mallet.cs.umass.edu/	
JSAT	https://github.com/EdwardRaff/JSAT	
ELKI	http://elki.dbs.ifi.lmu.de/	
Java-ML	http://java-ml.sourceforge.net/	
Accord	http://accord-framework.net/	C#, C++, C
Multiboost	http://www.multiboost.org/	
Shogun	http://shogun-toolbox.org/	
LibSVM	http://www.csie.ntu.edu.tw/∼cjlin/libsvm/	
mlpack	http://mlpack.org/	
Shark	http://image.diku.dk/shark/	
MLC++	http://www.sgi.com/tech/mlc/source.html	

Among the ML algorithms, a predictive model of regression has been used to phenotype Arabidopsis leaves, based on geometric features as a training dataset [158]. Three different algorithms were tested, k Nearest Neighbour (kNN), Support Vector Machine (SVM), and Naïve Bayes, to segment *Antirrhinum majus* leaves. Colour images have a characteristic vector intensity in the RBG and CIE L*a*b*, while the NIR vector is obtained with the wavelet transform. The best results were obtained with kNN for colour images and SVM for NIR. This shows that segmentation has several components, as mentioned before, including the wavelength of image acquisition [131].

As the specific wavelength used for image acquisition plays a key role in the type of data obtained, hyperspectral cameras are becoming important tools; however, hyper images can be in the order of gigabites of size, making ML a necessity. Examples of coupling hyperspectral and thermal imaging with ML have allowed the early detection of stress caused by *Alternaria* in Brassica [172]. The best image classification was obtained doing a second derivative transformation of the hyperspectral images together with a back propagation of neural networks, allowing the identification of fungi on leaves days after infection [172].

A current concept derived from ML is deep learning (DL), comprising a set of algorithms aimed to model with a high level of abstraction. This allows the development of complex concepts starting from simpler ones, thus getting closer to the idea of artificial intelligence (AI) [173]. Convolutional neural networks (CNN) are an example of DL derived from artificial neural networks (ANN). These multi-layered networks are formed by a layer of neurons that work in a convolutional way, reducing the sampling process to end with a layer of perception neurons for final classification [174]. Recently DL has been implemented using a CNN to automatically classify and identify different plant parts [175], thus obtaining both classification and localization that significantly improve the current methods. A CNN has been used to detect plant pathogen attacks [176]. Although the training period is computationally heavy, requiring several hours of CPU clusters, classification was performed in less than 1 second [176]. Nevertheless, DL is a step forward in ML and has great potential to allow the management and analysis of the data produced in phenomic experiments.

Although direct testing maybe the best way to determine the superior algorithm in each case, there are a number of examples that may guide initial approaches [33, 177, 178]. As a general rule, discriminating methods such as SVM, ANN, and kNN give better results in large datasets that are labelled [33]. Generative methods such as Naive Bayes, Gaussian mixture models, and Hide Markov models give better results with smaller datasets, both labelled and unlabelled. The use of unsupervised algorithms, i.e., k-means, may help identify unexpected characteristics of a dataset. As mentioned above, preprocessing plays a fundamental role in increasing the ML output. A summary of the complete pipeline of image analysis, including sensors, preprocessing, segmentation procedures, feature extractions, and machine learning algorithms, can be found in Table 4.

**Table 4:** A list of current procedures for image analysis based on the type of sensor used

Data type/source	Preprocessing	Segmentation	Feature extraction	Machine learning
Mono—RGB	*Homomorphic filtering to minimize illumination issues in outdoor images [198]	*Many vegetation indexes apply to segmentation in [132]	*Fourier descriptors and Zernike moments [200]	*ANN to detect Phalaenopsis seedling diseases [202]
	*Filtering and histogram equalization in plant disease detection [136]	*NDVI index to discriminate background and foreground [199]	*Statistical parameters and Wavelet transform with geometric characteristics [131]	*SVM to detect tomato leaf viruses [203]
		*Cellular neural networks edge detection [200]	*SIFT and SURF in 3D reconstruction images from multiple RGB cameras with basil specimen [167]	*Gaussian mixture model to detect biotic stress in wheat [204]
		*HSV algorithm [205]	*Histogram to color features and Fast Fourier Transform + Discrete Wavelet Transform to texture features extraction [145]	*k-NN to identify leaf disease [206]
				*Probabilistic Neural Networks and Genetic Algorithm [200]
				*Random forest to QTL analysis [207]
StereoVision	*Complete and general preprocessing pipeline [208]	*Otsu's method & growing region [209]	*Graph-cut and local correlation [210]	*SVM to identify diseased pixels in leaves [211]
	*Rectification of image based on SIFT and epipolar transformation, in vitis vinifera segmentation [212]	*SVM to remove background [211]	*SURF to stereo-match images based on their feature vectors [209]	*SVM & Gaussian Processes Classifier to detect soil moisture deficit [213]
	*Camera stereo calibration, leaf quantifying *Brassica napus* [214]		*Combined with thermal images (global and local features (temperature, depth, color) using PCA and analysis of variance [211]	
	*RGB2GrayScale [209]		*Simple statistical and intensity values [213]	
	*Align and depth estimation [213, 211]			
Multi-Hyper spectral	*Savitzky-Golay filter: remove noise and smooth the image [215]	*NDVI (750–705/750+705) nm with threshold of 0.20 [216]	*Pixels averaged to obtain average reflectance [216]	*Cascade of data mining techniques to detect foliar disease in barley leaves [217]
	*Gaussian filter to remove noise: detection of disease in banana leaves [218]			*Bayes, logistic, random forest, and decision trees to detect biotic stress in Alternaria genus [172]
	*Savitzky-Golay filter: detection of disease in plants [219]			*k-NN to identify leaf disease [206]
				*PCA and partial least squares regression to predict water, macronutrient, and micronutrient concentrations [216]
ToF	*Correction of the distance error caused by the extra contribution of electrons from sunlight using an offset parameter [68]	*Combine hierarchical color segmentation with quadratic surface fitting using ToF depth data [70]	*SIFT, Hough Transform, and RANSAC algorithms to extract relevant features [220]	
	* Carry out a calibration stage before fusing the depth data and color information [69, 70]	* The maximally stable extremal regions algorithm for the segmentation of single object over background in gray level images [221]		
	*Removal of spurious individual points (outliers) using statistical filter [74]	*Removal of background by simple thresholding pixel values greater than a certain threshold [220]		
	*Removal of lens distortion [220]	*Segmentation inspired from the maximally stable extremal regions algorithm [221]		
LIDAR	*RANSAC algorithm to detect ground plane [222]	*Clustering to detect individual plants [222]	*Statistical features from reflectace and geometry [222]	*ANN for Wheat Green Area Index measurement [223]
	*Reduction of noise, filtering point clouds based on deviation [224]		*Surface feature histograms to characterize the grapevine and wheat organs [225]	*ANN, SVM, logistic regression for plant identification (the best results) [222]
				*Generalized linear model (the best) to model plant richness [226]
				*SVM obtained a highly reliable classification of about 96% [225]
Thermography/Flourescence	*Align with stereo images (in combination with stereo images) [213, 211]	*Semi-automated segmentation through a geometric algorithm implemented in Python-based software ChopIt [227]	*Combined with thermal images (global and local features: temperature, depth, color) using PCA and analysis of variance [211]	*SVM to identify diseased pixels in leaves [211]
	*Normalize thermal information with thermal indexes [228]	*Manual thresholding comparing conventional color images with fluorescent images (Fv/Fm) [229]		*SVM and Gaussian Processes Classifier to detect soil moisture deficit [213]
	*Trimming extraneous images from image stack [227]			*Analysis of variance (not ML) to analyze different water status [228]
				*ANN and SVM to detect zinc deficiency stress using fluorescence imaging [230]
MRI/Tomography	*2D and 3D Fourier transformations (MRI) [231]	*Yang 2011: watershed segmentation [232]	*Intensity features, Haralick textural features, intensity local binary pattern features, contrast features, and Gabor intensity textural features [233]	*Supervised learning with ANN, Mahalanobis distance, linear discriminant analysis, and quadratic discriminant analysis to determine boundary lines [233]
	*Median filter, binaryzation, fill holes, remove small particles, and morphological filter (erosion) [232]	*Histogram thresholding method to binaryze the image [233]		
	*Re-slicing, cropping, and contrast enhancement [233]			

## Conclusions and Future Prospects

The implementation of phenomic technologies is a welcome change toward reproducibility and unbiased data acquisition in basic and applied research. A successful approach requires integrating sensors with wavelength and image acquisitions that will allow the proper identification of the items under analysis. A lot of work has been conducted in indoor setups, where reasonable conditions can be created to obtain high-quality images amenable to further processing. The difficulty with outdoor setups increases as a result of limitations in the actual image acquisition devices and the uncontrolled conditions that directly affect image quality. The new technologies such as the high-definition LIDAR or the multi-hyperspectral cameras have great potential to improve in the near future, especially in outdoor environments.

Preprocessing and segmentation data are 2 aspects of data treatment and acquisition that require careful design in order to avoid distortions and reproducibility [138]. As images are machine-produced data, but image types and processing procedures may be very different, the standardization of image capture, preprocessing, and segmentation may play an important role. Furthermore, a single procedure for image analysis cannot be considered a better choice, and it is the researcher that needs to assess the different algorithms to come up with an optimized procedure for their specific setup. It is a matter of time until databases with raw images become part of the standard in phenomics; using images very much like NCBI or Uniprot plays a key role in genomic and proteomic projects. With the decrease in the price of hyperspectral devices, new experiments may be performed that produce even larger datasets, and these datasets will have to go through artificial intelligence–based data analysis in order to give the researchers results interpretable by humans. We guess that, like in other omic approaches, there will be a confluence of standard procedures that are not currently common ground, making the current literature look intimidatingly diverse. Nevertheless, most of the basic processes described here are shared by the different experimental setups and data analysis pipes.

## Abbreviations

AI: artificial intelligence; ANN: artificial neural networks; CAI: Cellulose Absorption Index; CAR: chlorophyll absorption ratio; CCD: charge coupled device; Cig: Coloration Index green; Cir: Coloration Index red; CMOS: complementary metal oxide semiconductor; CNN: convolutional neural networks; CPU: central processing unit; DL: deep learning; DLAI: Difference Leaf Area Index; DSWI: disease water stress index; DWT: Daubechies wavelet transform; EVI: Enhanced Vegetation Index; FAIR: Findability, Accessibility, Interoperability, and Reusability; GI: Greenness Index; GMM: Gaussian mixture model; GNDVI: Green Normalized Difference Vegetation Index; HoG: Histograms of Oriented Gradients; KNN: K nearest neighbour; LAI: Leaf Area Index; LCA: Lignin-Cellulose Absorption Index; LIDAR: light detection and ranging; LWVI-1: Normalized Difference Leaf water VI 1; MCARI: Modified Chlorophyll Absorption Ratio Index; MCFI: multicolour fluorescence imaging; ML: machine learning; NDVI: Normalized Difference Vegetation Index; NIR: near infrared; NLI: Nonlinear Vegetation Index; NTDI: Normalized Tillage Difference Index; OSAVI: Optimized Soil Adjusted Vegetation Index; PCA: principal component analysis; PET: positron emission tomography; PWI: Plant Water Index; QTL: quantitative trait locus; RF: radiofrequency; RGB: red, green, blue; RT: real-time; SIFT: Scale Invariant Features Transforms; SURF: Speeded-Up Robust Features; SVM: Support Vector Machine; TDI: time delay and integration; ToF: time of flight; UV: ultraviolet.

## Competing interests

The authors declare they have no competing interests.

## Funding

This work was funded by grants FEDER BFU-2013–45 148-R, Fundación Séneca 19 398/PI/14 to MEC and FEDER ViSelTR (TIN2012–39 279) to PJN.

## Author contributions

F.P.S., M.E.C., and P.J.N. defined the scope of the manuscript. F.P.S., M.E.C., and P.J.N. wrote and corrected the manuscript. M.E.C. and P.J.N. wrote the grant applications.

## Supplementary Material

GIGA-D-17-00043_Original-Submission.pdf

GIGA-D-17-00043_Revision-1.pdf

GIGA-D-17-00043_Revision-2.pdf

Response-to-Reviewer-Comments_Original-Submission.pdf

Response-to-Reviewer-Comments_Revision-1.pdf

Reviewer-1-Report-(Original-Submission).pdf

Reviewer-1-Report-(Revision-1).pdf

Reviewer-2-Report-(Original-Submission).pdf

Reviewer-2-Report-(Revision-1).pdf
