# Supplementary material for: Plant phenomics: an overview of image acquisition technologies and image data analysis algorithms
Source: Gigascience. 2017 Oct 3;6(11):1–18. doi: 10.1093/gigascience/gix092 (PMC5737281; doi:10.1093/gigascience/gix092)

GigaScience

Phenomics: an overview of image acquisition technologies and image data analysis algorithms

--Manuscript Draft--

**Manuscript Number:**	GIGA-D-17-00043
**Full Title:**	Phenomics: an overview of image acquisition technologies and image data analysis algorithms
**Article Type:**	Review
**Funding Information:**	Ministerio de Economía y Competitividad (ES) (BFU-2013-45148-R) — Prof.Dr. Marcos Egea-Cortines<br>Ministerio de Economia y Competitividad (TIN2012-39279) — Prof Pedro Javier Navarro<br>Fundación Séneca (19398/PI/14) — Prof.Dr. Marcos Egea-Cortines
**Abstract:**	The field of automatic phenotype acquisition technologies or phenomics has seen an important advance in the last years. As other omic technologies, it bears from a common set of problems, including data acquisition and analysis. In this review, we give an overview of the main systems developed to acquire images, and we give an in-depth analysis of image processing with its major issues, and the algorithms that are being used or emerging as useful to obtain data out of images in an automatic fashion.
**Corresponding Author:**	Marcos Egea-Cortines, PhD<br>Universidad Politecnica de Cartagena<br>Cartagena, Murcia SPAIN
**Corresponding Author Secondary Information:**	
**Corresponding Author's Institution:**	Universidad Politecnica de Cartagena
**Corresponding Author's Secondary Institution:**	
**First Author:**	Fernando Perez-Sanz, MsC
**First Author Secondary Information:**	
**Order of Authors:**	Fernando Perez-Sanz, MsC<br>Pedro Javier Navarro, PhD<br>Marcos Egea-Cortines, PhD
**Order of Authors Secondary Information:**	
**Opposed Reviewers:**	
**Additional Information:**	
**Question**	**Response**
Are you submitting this manuscript to a special series or article collection?	No
**Experimental design and statistics**<br><br>Full details of the experimental design and statistical methods used should be given in the Methods section, as detailed in our Minimum Standards Reporting Checklist. Information essential to interpreting the data presented should be made available in the figure legends.	No

Powered by Editorial Manager® and ProduXion Manager® from Aries Systems Corporation

Have you included all the information requested in your manuscript?	
If not, please give reasons for any omissions below.<br><br>as follow-up to "**Experimental design and statistics**<br><br>Full details of the experimental design and statistical methods used should be given in the Methods section, as detailed in our Minimum Standards Reporting Checklist. Information essential to interpreting the data presented should be made available in the figure legends.<br><br>Have you included all the information requested in your manuscript?<br><br>"	Not aplicable
**Resources**<br><br>A description of all resources used, including antibodies, cell lines, animals and software tools, with enough information to allow them to be uniquely identified, should be included in the Methods section. Authors are strongly encouraged to cite Research Resource Identifiers (RRIDs) for antibodies, model organisms and tools, where possible.<br><br>Have you included the information requested as detailed in our Minimum Standards Reporting Checklist?	Yes
**Availability of data and materials**<br><br>All datasets and code on which the conclusions of the paper rely must be either included in your submission or deposited in publicly available repositories (where available and ethically appropriate), referencing such data using a unique identifier in the references and in the “Availability of Data and Materials” section of your manuscript.<br><br>Have you have met the above requirement as detailed in our Minimum	Yes

Standards Reporting Checklist?	

Powered by Editorial Manager® and ProduXion Manager® from Aries Systems Corporation

Manuscript

Click here to download Manuscript gigascience_Fer_Final.docx

Gigascience Review

Phenomics: an overview of image acquisition technologies and image data analysis algorithms

Fernando Perez-Sanz1, Pedro J. Navarro2, Marcos Egea-Cortines2

[1]Genetics, ETSIA, Instituto de Biotecnología Vegetal, Universidad Politécnica de Cartagena, 30202 Cartagena, Spain

[2]DSIE, Universidad Politécnica de Cartagena, Campus Muralla del Mar, s/n. Cartagena 30202, Spain

email- Fernando Perez-Sanz- fernando.perez8@um.es; Pedro J. Navarro pedroj.navarro@upct.es

Correspondence Marcos Egea-Cortines marcos.egea@upct.es

Abstract

The field of automatic phenotype acquisition technologies or phenomics has seen an important advance in the last years. As other omic technologies, it bears from a common set of problems, including data acquisition and analysis. In this review, we give an overview of the main systems developed to acquire images, and we give an in-depth analysis of image processing with its major issues, and the algorithms that are being used or emerging as useful to obtain data out of images in an automatic fashion.

Keywords: algorithms; artificial vision; deep learning; hyperspectral cameras; machine learning; segmentation

Background

The development of systems to monitor large fields using NDVI, started a long successful career over 25 years ago when NDVI was used in the so-called remote sensing field [1]. It was an important milestone in the advancement of automatic methods to analyse plant growth and biomass. Ever since, new technologies have increased our capacity to obtain data from biological systems. The ability to measure chlorophyll status from satellite images allowed to assess large field plant health and predict crops and productivity in very large areas such as the Canadian prairies, Burkina Faso or the Indian Basin in Pakistan [2–5]. Thus, the field of remote sensing is an important basis where knowledge about data acquisition and analysis started. The development of phenotyping devices using local cameras for crops took off using an array of technologies including Infrared thermography to measure stomatal opening or osmotic stress [6–8]. Analysis of direct imaging has been developed to study root development [9–11], and has found a niche to identify germplasm resistant to abiotic stresses in plants such as cereals [12], Arabidopsis [13] and for large-scale field phenotyping [14]. There are several recent reviews addressing the different types of setups [15–21], and we will not cover them in the current review.

The use of high-throughput screening systems based on imaging techniques had a major impact in the identification of mutants involved in different processes. Historically, the first type of screenings was developed using the Luciferase reporter gene driven by a promoter. Upon mutagenesis of a parental line harbouring a regulatory region activated or repressed by a certain biological process or an environmental condition, new germplasm has been recovered. This allowed the identification of a large number of mutants affecting complex traits such as response to abiotic stress [22] or circadian clock [23]. A second type of analysis based on measuring growth helped identify genes involved in chloroplast function [24]. Further studies using promoters driving a reporter gene have been used in Bryophytes such as *Physcomitrella patens*, or the unicellular green Algae *Chlamydomonas reinhardti* to study circadian regulation [25,26]. Complex screens have been set up for instance to identify the formation of Cajal bodies in nuclei using alternatively spliced GFP protein variants [27]. Once promoter driven lines are established they can be reused for further studies. A screen of 720 chemical compounds was performed in Arabidopsis plants with a GIGANTEA promoter driving luciferase identified compounds that affect circadian clock and cause actin stabilization, an otherwise difficult parameter to measure [28]. Altogether, these screens have proven the importance of unbiased image acquisition systems, demonstrating the universal power of this approach for in-depth research in plants. Those studies based on transgenic material have been extensively used model systems such as Arabidopsis, *Physcomitrella* or *Chlamydomonas*, and it is also its major drawback for crops.

Both large field and green-house or growth chamber setups have in common the increasing number of images produced. Two major aspects to consider are the type of image acquired and how to handle it. In this review, we cover the main types of image acquisition devices and the current and emerging methods of image data analysis.

Review

Image acquisition

Image acquisition is the process through which we obtain a digital representation of a scene. This representation is known as image and its elements are called pixels (picture elements). The electronic device used to capture a scene is known as imaging sensor. CCD (charge-coupled device), CMOS (complementary metal oxide semiconductor) and TDI (time delay and integration) are the most broadly used technologies in image sensors. A light wavelength is captured by small analogic sensors, which will acquire major or minor charge depending on the amount of incident light. These signals are amplified, filtered, transported and enhanced by means of specific hardware. A suitable output interface and a lens in the same housing is all that it is needed to perform image acquisition. The elements enumerated above conform the main element of computer vision systems, the camera.

The aforementioned technologies, CCD, CMOS and TDI confer unique characteristic, which define the type of usage a camera can provide. There are fundamental differences in the type of performance the different sensors offer. In the last years CMOS technology, has outperformed CCDs in most visible imaging applications. TDI with CCD imaging sensor is used for high speed and low light level applications and CCDs are used to capture NIR images and line scan applications [29].

The field of image acquisition is extremely developed with considerable literature but image acquisition systems can be classified into five groups that are suitable for phenotyping.

1. Mono vision

Mono-vision systems are composed by a set comprising a lens, imaging sensor, specific hardware and IO interface. Depending if they use a line or matrix of pixels, they are classified in line cameras (or scanners) and matrix cameras. Most computer vision phenotyping devices are based on mono vision systems. Examples of mono vision devices include SPICY, an automated phenotyping prototype of large pepper plants in the greenhouse. The system uses multiple RGB cameras to extract two types of features: (1) features from a 3D reconstruction of the plant canopy; and (2) statistical features derived directly from RGB images [30]. A different approach has been used with two

cameras inside a growth chamber to measure the features of *Petunia, Antirrhinum* and *Opuntia*. Using configurable periods of time simulating days and nights, coupled to software and hardware development allow to measure large temporal series of nutation and growth speed of wildtype and mutants and changes in circadian regulation [31]. Recently a high-throughput RGB system has been developed to quantify QTLs involved in yield in large recombinant inbred lines in maize [32], demonstrating the increasing impact of this approach to obtain elite lines.

2. Stereo vision

Stereo vision systems try to correct a drawbacks of mono vision systems for measuring of distances. Architecture of stereo vision systems emulate the behaviour of human vision using two mono vision systems. Basically, and after locating a point in two mono vision systems, it is possible to compute the distance from the point to the system. Images produced are known as deep maps. A stereo vision system has been used by Biskup and colleagues [33] to obtain structural features of plant canopies. Stereo vision coupled with ToF images have been implemented to increase the performance of methods of image segmentation [34]. The 3D reconstruction has been successfully employed to obtain 3-D models of plants, thus demonstrating the power of this approach [35]. Simple depth reconstructions helped to define stems, leaves and grapes showing the potential of this technology [36]. A stereo-vision has been developed to perform high throughput analysis of rape leaf traits. The system uses two identical RBG cameras to obtain stereo images for canopy and 3-D reconstruction[37]. Developing a 3D-mesh segmentation has allowed to analyse cotton growth [38], further showing the possibilities of 3D imaging. Nevertheless, the major drawback of stereo vision systems and 3D reconstruction is its low throughput as compared to 2D systems.

3. Multi and hyper spectral cameras

Multispectral cameras are devices able to capture images of a number of discrete spectral bands, usually between 2 and 10. The spectral bands may not be continuous, thus for one pixel we obtain a vector of information comprised by the number of elements corresponding to the number of bands registered. Hyperspectral systems

obtain for each pixel a digital signature that may contain several hundreds of continuous bands within a specific range of wavelengths [39]. Both multispectral and hyperspectral imaging have been used for remote sensing and have an increased number of applications in phenomics. A multispectral system has been developed to improve the original color of images for fruit recognition [40]. The authors fused the original color image with an infrared image using nonlinear Daubechies wavelet transform (DWT). Thus, the additional information from a second image allows improving the first original one.

The use of hyperspectral cameras is increasing in phenotyping experiments as they allow the identification of physiological responses, pathologies or pests in a non-invasive way. Using hyperspectral images, a system has been developed to identify pathogens in barley leaves using probabilistic topic models [41]. A detailed description of the different wavelengths and combinations used in multispectral and hyperspectral cameras can be seen in Figure 2, and their uses in Table 1.

4. ToF cameras

The Time of Flight cameras or ToF has as a general principle the measurement for each pixel of an image of the distance of the object to the camera. This is achieved measuring the time it takes for a signal emitted in NIR to come back, reflected by the object. This allows a precision 3D reconstruction. This type of systems have been used in combination with stereo images to obtain leaf areas [34]. Beyond the tedious hand work required for manual analysis sampling is done in a non-destructive way.

The main disadvantage of ToF cameras is the low resolution and the high dependence on the reflecting surface for imaging. As a result, they cannot operate under strong sunlight and are more appropriate for indoor conditions.

5. LIDAR technology

Light Detection and Ranging (LIDAR) is a remote sensing technology developed at the beginning of the 70s to monitor the Earth Surface. These systems use a laser pulse light to measure the distance between the light source and the object by measuring the time of emission and time of reflected light detection. It allows the creation of a cloud of points that reconstruct the 3D structure of an object

Currently LIDAR technologies are being implemented beyond the original usage of surface recognition and land digital modelling. These include autonomous vehicle guidance [42] or a large number of applications in geology and ecological studies [43]. It is an emerging technology with a future impact on phenotyping.

6. Thermography and Fluorescence Imaging

Thermography is a widely-utilized technology in remote sensing and plant phenotyping. Thermographic cameras are able to acquire images at infrared wavelengths of 14.000 nm (1.4e-5 m), thus allowing the conversion of the irradiated energy into temperature values, once the environmental temperature is assessed. Plants open stomata in response to environmental cues and circadian clock depending on the type of photosynthetic metabolism they have, thus emitting water vapour causing evapotranspiration. The evapotranspiration can be assessed with thermography [44], and quantification can be made at different scales such as a leaf, a tree, a field or a complete region.

The multicolour fluorescence is a non-invasive technique that allows to capture autofluorescence produced in the plant as a result of UV light excitation. In contrast to the widely used thermographic techniques, multicolour fluorescence (MCFI) has been used mostly in basic research and in very few cases has found its way into field experiments [45,46]. In the last years, the large scale implementation is getting attention [47]. The combination of MCFI and thermography has been used in a model to predict plant health in zucchini [48] showing a precision of 85-100% to detect infections or plant pathologies.

Image analysis

Extracting information from images is performed through the process of segmentation. The aim of a segmentation procedure is to extract the components of an image that are of interest from the rest of the image. Thus, we end up with a partitioned image with significant regions. The significant regions may be defined as foreground versus background, or by selecting a number of individual components from an image. The construction of the selected regions is based on the image characteristics such as colour (colour spaces), spectral radiance (vegetation indexes), edge detection, neighbour similarity [49] or combinations that are integrated via a machine learning process [50]. In some cases, a pre-processing is required in order to obtain a meaningful segmentation. . A basic workflow performed in image processing is represented in Figure 1.

Image pre-processing:

Pre-processing is a large field of work in artificial vision. Here we give an overview of the most common ways to pre-process and discuss the major pitfalls. A major task of pre-processing is to obtain an estimation of the regions of interest. It can have a positive effect over the quality that is under study, and may increase substantially the image quality and the parameter under analysis. However, there are two major drawbacks. First pre-processing may cause an important loss of information and second it may cause distortions in the images when applied incorrectly [51]. It is important to remind that pre-processing does not increase the information in an image. In fact, when information is measured as entropy, pre-processing causes a decrease in entropy. As a result, an alternative to pre-processing is to obtain high quality images that will allow segmentation without the pre-processing step. Pre-processing maybe nevertheless required and has a set of algorithms that may perform corrections and enhancements [51].

During image acquisition, artefacts may be generated in the images requiring corrections prior to measurement and characterization. Amongst the typical corrections are:

- Sensor corrections due to distortions of the lenses or loss of pixels.
- Light corrections to improve contrast or eliminate shades.
- Noise elimination.
- Geometric corrections via rotations or other geometric distortions of the image.
- Colour corrections via redistribution of the intensity values of the different channels.

Image enhancement is used to optimize or facilitate the measurement of certain characteristics of interest. Amongst the most common procedures for image enhancement are:

- Global enhancement of illumination via LUT remapping, local enhancement via gradient filters, histogram equalization or range filters.
- Image filtering via focussing and blurring or smoothing. In some cases, processes such as image rotation cause distortions either blurring or over-marking the edges of an image. This may be corrected via the opposite operation i.e. sharpening or blurring.
- Morphological operations where pixels are calculated according to values of neighbour pixels. Two morphological operations routinely used are dilation and erosion and the corresponding derived procedures opening and closing. Morphological gradients maybe used also as a morphological operation.

Although image pre-processing is a very important component of image analysis, it is not well documented in the phenomics field or in plant biology. It could be that it is a newly developed step or that it is assumed that it must be used in many cases. However, we believe that the impact on data analysis and downstream processing is big enough to warrant at least a description of the procedure in order to achieve reproducible results in the emerging field of image processing for phenomics where big data dimensions can be easily reached in relatively small experiments.

Vegetation indexes:

Vegetation indexes (VIs) are combination of reflectance of two or more wavelengths aimed at enhancing one property of vegetation that needs to be measured. Currently there are over 150 publications of VIs in the literature, where Vis have been shown to be in good correlation with different parameters such as Leaf Area Index (LAI), biomass, chlorophyll concentration, photosynthetic activity etc.

The best known is the Normalized Difference Vegetation Index (NDVI). It is based on the contrast between the maximal absorption of red light by chlorophyll and maximal reflexion of near infrared by the cellular structures of the leaf [52].

$$NDVI = \frac{R_{800} - R_{670}}{R_{800} + R_{670}}$$

Where R_{800} is the reflectance at 800 nm and R_{670} the reflectance at 670 nm. Although the exact wavelengths maybe adjusted to the object under study and the available sensors, the most common wavelengths used are the ones mentioned here [53]. Despite being a standard way of measurement it has a major drawback of signal saturation at high vegetation density, thus underestimating all parameters beyond a threshold.

As a result, other indexes have been developed such as the Renormalized Differences Vegetative Index (RDVI) or the Modified Simple Ratio (MSR). Both have been designed in order to linearize signals and biophysical variables.

RDVI combines the advantages of the vegetation index by subtraction and the normalized index (NDVI):

$$RDVI = \frac{R_{800} - R_{670}}{\sqrt{(R_{800} + R_{670})}}$$

Where NIR is near infrared at 800nm and VIS is visible 670nm.

MSR was proposed as an improvement over RDVI in terms of enhanced sensitivity so biophysical parameters based on a simple NIR/red ratio.

$$MSR = \frac{\left({R_{800}}/{R_{670}}\right) - 1}{\sqrt{\left({R_{800}}/{R_{670}}\right) + 1}}$$

EVI (enhanced vegetation index) was developed by NASA in 1999 and improves NDVI using similar calculations. However it incorporates corrections for distortions caused by air particles [54].

$$EVI = \frac{2.5\ (\ NIR - RED\ )}{(\ NIR + 6 \cdot RED\ -\ 7.5 \cdot BLUE + 1)}$$

The previous indexes have been used in remote sensing. However, the phenotyping at the greenhouse scale or growth chamber has different technical challenges. One problem of segmentation in phenotyping is the heterogeneity of the pictures that make difficult the process (see Hartmann et al [55] for a complete discussion). Indeed RGB images have as major advantage a high resolution but as they capture background information it can be difficult to obtain accurate segmentations [56].

Plant image acquisition for accurate phenotyping of aerial organs has the additional problem of organ movement, a feature uncovered by Charles Darwin [57]. Plant movement maybe circadian controlled but is also affected by environmental conditions such as gravity or light [58,59]. This causes a challenge to identify the true size or image signal of the organ under study. Different technical solutions have been provided. Using a stereo-based imaging, the angle of soybean leaves, and the effect of drought stress has been dissected [33]. Further analysis using 3-D imaging has allowed the study of leaf disks and establish by comparison to intact leaves, the importance of local signalling for growth [60].

A different approach to the problem of growth is based on mathematical treatment of data. The shoot apical meristem is the region where a group of stem cells control lateral organ formation in higher plants [61]. The growth of the stem occurs at a certain distance from the top and displaces itself continuously requiring the continuous adjustment of the Region of Interest (ROI) to be measured via a mathematical adjustment of the image [31]. This type of dynamic ROI has been an important issue and software and hardware technologies have been developed for tracking mobile devices such as cars or unmanned vehicles using ROI [62,63].

A new field is the identification of physicochemical characteristics in a non-invasive manner [64]. This type of data acquisition requires the development of specific indexes for certain purposes that may need *ad-hoc* solutions. A method to detect water stress in plants has been implemented based on short wave infrared hyperspectral images (SWIR) [65]. Thus, images obtained in the 1500-1590 nm ranges divided by images in the 1390-1430nm give a high contrast between leaves with different hydric status. The use of vegetation indexes have been proposed as a mean to segment images and automatically identify brown spot and blast disease in rice [66].

The use of hyperspectral cameras opens new possibilities to explore new spectral ranges and bandwidth combinations. Indeed a new probabilistic model based on a spectral range of 400-1000nm called “wordify” has been developed to identify leaf pathogens [67].

Image segmentation

As we mentioned above, image segmentation is the core of image processing for artificial vision-based plant phenotyping. Segmentation allows the isolation and identification of objects of interest from an image, and it aims to discriminate background or irrelevant objects. The objects of interest are defined by the internal similarity of pixels in parameters such as texture, colour, statistic [68], etc. See a list of Open software libraries for image segmentation in Table 2.

Colour is one of the main features used to detect leaves in the visible spectrum (VIS). Many images are represented in the RGB colour space, but it is not always easy to find a region of interest within this specific space. A transformation to hue, saturation, value (HSV) has been proposed [69] where hue represents the colour tone, saturation is the distance for the colour to white and value is the luminosity. In this transformation, hue can discriminate to detect chlorophyll, being the other two parameters of lesser importance, thus reducing information to one dimension. As a complement to the change in colour space, some authors propose a dynamic approach instead of using fixed thresholds based on a single colour component, using for that purpose the analysis of colour histogram peaks [69–71]. Nevertheless, the HSV transformation includes an improvement approximating the colour histogram by a weighted sum of Gaussian probability density functions.

$$h(.) \approx \sum_{i=1}^{m} \alpha_i N(\mu_i, \sigma_i^2)$$

where α_i is the weighting parameter and N (μ, σ^2) is the Gaussian probability density function with average μ and variance σ^2.

The Watershed transformation is a popular algorithm form segmentation. It treats an image as topological surface that is flooded, and seed regions are included, usually by the user. This generates an image with gradients of magnitudes, where crests appear in places where borders are apparent (strong edges), and causes segmentation to stop at those points [49]. A modified Watershed algorithm has been implemented to identify partially covered leaves [72]. These modifications include pre-flooding, morphological, criteria with pre-flooding and aperture, and criteria for limits on concavity in aperture. Whilst the pre-processing has a positive effect on the ratio of detection, it may not be an ideal algorithm in cases where there is a local minimum or two or more unconnected regions. A different approach has been used by segmentation of the background image via a neural network followed by Watershed to discriminate rosette leaves in Arabidopsis as individual objects [73].

Grabcut [74] is a segmentation algorithm based on graph cut [75]. It is based on graph theory to tackle the problem of separating an object or foreground from the background. The user should mark a rectangle (bounding box) surrounding the object of interest thus defining the outrebound of the box as background. The inner part or bounding box contains an undefined combination of background and foreground. The following steps require the identification of the edges between the object or foreground and the background using standard minimum cut/maximum flow algorithms [51]. The major advantage of grabcut over graph cut relies on the possibility of using it over colour images using a Gaussian Mixture Model (GMM). It replaces the l min cut algorithm by an iterative process of estimation and learning of the parameters. This algorithm has been tested to extract trees from a figure but it has been successful only with very simple backgrounds. More recently grabcut has been used together with two additional segmentation algorithms (otsu and colour sliding) [64] within a more complex workflow for plant recognition based on leaf structure, producing substantially better results. However, we have to consider that in contrast to the work described for tree image extraction [76], the image background was black and uniform, and background complexity is key for algorithm outputs.

Snakes are a special type of active contours [77], and are used as methods to fit lines (splines) either to open or close edges and lines in an image. The mathematical representation is a curve v[s]=[x(s), y(s)] moving in space via t iterations. The equation has a parameter (s) related to the variables x, y. Considering t as a second parameter, the curve is defined as v[s, t]=[x(s, t), y(s, t)], where s is the space occupied by the curve and t the number of iterations. These methods have been used for face recognition, iris segmentation or medical image analysis. Within the field of plant phenotyping, there are procedures where active contours are used inside a protocol constructing a vector of features with data of colour intensity, local texture and a previous knowledge of the plant incorporated via Gaussian Mixture Models, previously segmented [78]. These steps give an initial rough segmentation upon which, active contours can operate with a much higher precision.

Active contours have used for plant recognition via images of flowers [79], based on a combination of the algorithm proposed by Yonggang and Karl [80] and the model of active contours without edges [81]. Whilst the work proposed by Minervini [78] appears to give significantly better results as those of Suta [79], the usage of images with a natural background maybe related to the apparent differences in segmentation. Thus, a current problem concerning the comparison of algorithms and procedures lies on the different backgrounds used for image acquisition.

Recently an optimization of the Chan-Vese model has been proposed for leaf segmentation [82]. The rationale behind the optimization is based on a combination of local statistics and an improvement of the energy function. The local statistics are obtained with grey scale images and average values using a 5x5 pixel mask. This procedure decreases the lack of homogeneity that may be found in intensity values in the leaves. The local information obtained is added to the internal energy function, decreasing the deviation of the level set function of the Signed Distance Function (SDF) thus optimizing the requirement to reinitiate SDF.

Characteristic extractions based on detection of points of interest

There are a number of algorithms to identify invariant features detectors and descriptors. This type of image analysis ensures the detection of points of interest in a scale and rotation independent manner. This is crucial for camera calibration. Furthermore, it allows the identification of points of interest even when they change scale and/or position, a common issue when phenotyping plants.

The Scale Invariant Features Transforms (SIFT) [83] is an algorithm widely implemented to extract characteristics. It is used either directly or as a base to develop other algorithms. Despite being around for over a decade, SIFT took off as a technique in the last two or three years. SIFT defines each extracted datum as a characteristic described by position, scale, orientation and descriptive vector, and has four stages of characteristics extractions:

1) Maxima and minima detection of a scale. This first stage defines the localization and scale of the potential points of interest using Difference of Gaussian functions (DoG).

2) Location of the points of interest (key points). Candidate points are localized and refined, eliminating those with low contrast.

3) Orientation assignment. The orientation of the key points is calculated with local gradients in the image.

4) Generation of a descriptor for key points. This last stage computes a local descriptor of the image for each point based on the gradient magnitude and the orientation of each point in the image.

Speeded-Up Robust Features (SURF) [84] is a widely used algorithm to extract characteristics. It is based on the same principles as SIFT. However, it has a different scheme, geared towards processing speed. SURF starts detecting possible points of interest and their localization. Very much like SIFT it determines first the scale following by orientation and finishes with the descriptor. Scales are computed with Hessian matrix but with a very basic approximation. Once scaling is achieved, it uses the wavelet transform with de Haar base for orientation. This allows to obtain key points that are invariant to rotation, illumination and orientation. The method is highly robust as key points can be considered of interest only if they are detected by different parameters (scale, orientation, etc).

Comparing both algorithms [85] in a set of two images SIFT detects more points of interest (892-934) than SURF (281-245). However, the speed of analysis is three-fold lower in SIFT (1.543 s Vs 0.546 s). Furthermore, the number of matching feature was 41 in SIFT and 28. This is roughly 4.5% in SIFT versus 10.64% in SURF. So, despite detecting a lower number of points, the speed of execution and percentage of coincidences is higher in SURF, compared to SIFT.

A way to reduce computing time and memory is by using algorithms that decrease the dimensionality of the data such as Principal Component Analysis (PCA) of the descriptors. A second one is by converting the descriptors defined in float point to a binary string. This requires to compute the complete set of descriptors before further processing. BRIEF (Binary Robust Independent Elementary Features) [86] improves processing time via directly computing binary strings from image patches. This is achieved via direct comparison of intensities of pairs of points on a single line without previous training. Ethan Rublee et al [87] developed Oriented FAST and Rotate BRIEF (ORB), a fusion of the point detector FAST and the descriptor BRIEF with some modifications. It uses FAST ab initio [88] in order to identify points of interest. It uses Harris corner measure to define the N best points amongst the set localized. As FAST does not compute the orientation, it is obtained by the average intensity of the centroid of a window that has a corner in the centre (intensity weighted centroid of the patch with located corner at center). The vector direction gives the orientation. As FAST is not rotation invariant, the momentum of x and y are calculated and should be within a circle with ratio of the size of the window. Data is processed further with BRIEF descriptors but as BRIEF has a poor performance with rotations, the algorithm is guided according to the orientation previously computed

A direct comparison of the three algorithms show that ORB is the fastest while SIFT has the best performance [89]. When rotation angles equal 90º ORB and SURF override SIFT. On the other hand, images with noise are equally computed by ORB or SIFT. ORB tend to show points of interest concentrated in the centre of the image while SIFT and SURF tend to show scattered points distributed throughout the image.

The Histograms of Oriented Gradients (HOG) [90] is a characteristic descriptor used for object detection. It is based on counting events in orientation gradients, in localized portions of an image. The way the algorithm works in a resumed manner is as follows:

1- Images are divided in small connected regions or cells. A gradient histogram is computed for each cell.

2- Each histogram is discretized in angular bins according to the orientation gradient.
3- Each pixel in a cell contributes to the gradient in a weighted manner taking into account the angular bin.
4- Neighbour cells are considered special regions or blocks that are further used to group and normalize the histograms.
5- The groups of normalized histograms represent a block and give the descriptor.

There are few works were these type of algorithms have been used for plant phenotyping, and their main use has been in 3D plant image reconstruction [91–93]. However, they have great potential for automatic species recognition, identification of overlapping organs or pathogen localization.

Massive segmentation

The amount of data that are generated in current and future phenomic setups with high throughput imaging technologies has brought the use of Machine Learning (ML) statistical approaches. Machine Learning is applied in many fields of research [94–96]. As phenotyping can generate Terabytes of information, ML tools provide a good framework for data analysis. A list of ML libraries can be found in Table 3.

Typically, the ML analysis process starts with a set of data that should be big enough to generate a model. A portion of the dataset is used as training dataset to create a model, while the rest of the dataset or testing dataset is used to test and validate the model. If the precision of the model is high enough it can be used with new data for identification, prediction, quantification etc [97].

There are two fundamental techniques in ML, supervised and unsupervised. In supervised ML models, the training data are tagged by the user. Thus, the user may previously choose one or several images and generate a series of vectors that are tagged and represent foreground and background. These characteristic vectors are used to train the ML algorithm. In unsupervised ML techniques, the function will categorize

pixels with similarities creating clusters that have low intracluster distance compared to intercluster distances.

There are several types of ML algorithms, thus the selection of the one to use may require actual experimentation for optimal results. It is important the definition of a set of training data that is representative enough to obtain successful classification.

Among the ML algorithms a predictive model of regression has been used to phenotype Arabidopsis leaves, based on geometric features as training dataset [98].

Three different algorithms were tested, k Nearest Neighbour (kNN), Support Vector Machine (SVM) and Naïve Bayes to segment Antirrhinum majus leaves [50]. Colour images have as characteristic vector intensity in the RBG and CIE L*a*b*, while the NIR vector is obtained with the wavelet transform. The best results were obtained with kNN for colour images and SVM for NIR. This shows that segmentation has several components as mentioned before including wavelength of image acquisition.

As the specific wavelength used for image acquisition plays a key role in the type of data obtained, hyperspectral cameras are becoming important tools, however, hyperimages can be in the order of Gbites of size, making ML a necessity. Examples of coupling hyperspectral and thermal imaging with ML have allowed the early detection of stress caused by *Alternaria* in *Brassica* [99]. The best image classification was obtained doing a second derivative transformation of the hyperspectral images together with a back propagation of neural networks allowing the identification of fungi on leaves days after infection [99].

A current concept derived from ML is Deep Learning (DL) comprising a set of algorithms aimed to model with a high level of abstraction. This allows the development of complex concepts starting from simpler ones, thus getting closer to the idea of Artificial Intelligence (AI) (www.deeplearningbook.org). Convolutional neural networks (CNN), are an example of DL derived of artificial neural networks (ANN). These multi-layered networks are formed by a layer of neurons that work in a convolutional way reducing the sampling process and end with a layer of perception neurons for final classification [100]. Recently DL has been implemented using a CNN to automatically classify and

identify different plant parts [101], thus obtaining both classification and localization that improve significantly the current methods. A CNN has been used to detect plant pathogen attacks [102].

In order to test the applicability of CNN to identify pathogen attacks in leaves [102], two CNN architectures have been analyzed: AlexNet [103] y GoogLeNet [104] with a dataset of 54,306 from PlantVillage [105]. The accuracy obtained was 85.53% using AlexNet::TrainingFromScratch::GrayScale::80-20 (80% data train, 20% data test) and 99.34% using GoogLeNet::TransferLearning::Color::80-20. The advantage of this technique is the high accuracy without having to extract characteristics datasets to train the system. Although the training period is computationally heavy, requiring several hours of CPU clusters, classification was performed in less than one second. This model has limitations as testing a set of images under different training conditions causes a decrease in precision to 31.4%. An additional constrain is that it is currently restricted to single leaves facing up, and real applications should ideally classify directly from whole plants. Furthermore, plant pathogens may show visible phenotypes in plant parts other than the abaxial part of the leaf.

Nevertheless, DL is a step forward in ML and has great potential to allow the management and analysis of the data produced in phenomic experiments.

Conclusions and future prospects

The implementation of phenomic technologies is a welcome change towards reproducibility and unbiased data acquisition in basic and applied research. A successful approach requires integrating sensors, with wavelength and image acquisitions that will allow the proper identification of the items under analysis. The pre-processing and segmentation of data are two aspects of data treatment and acquisition that require careful design in order to avoid distortions and reproducibility. With the decrease in price of hyperspectral devices, new experiments may be performed that produce even larger data sets, and these data sets will have to go through Artificial Intelligence-based data analysis in order to give the researchers results interpretable by humans. We guess that like in other omic approaches, there will be a confluence to standard procedures

that currently are not common ground, making the current literature look intimidatingly diverse. Nevertheless, most of the basic processes described here are shared by the different experimental setups and data analysis pipes.

Abbreviations

AI: Artificial intelligence

ANN: Artificial neural networks

BRIEF: Binary robust independent elementary features

CAI: Cellulose Absorption Index

CAR: Chlorophyll absorption ratio

CCD: Charge coupled device

Cig: Coloration green

Cir: Coloration Index red

CMOS: Complementary metal oxide semiconductor

CNN: Convolutional neural networks

CPU: Central processing unit

DL: Deep learning

DLAI: Difference Leaf Area Index

DoG: Difference of Gaussian

DSWI: Disease water stress index

DWT: Daubechies wavelet transform

EVI: Enhanced vegetation index

FAST: Features from accelerated segment test

GI: Greenness Index

GMM: Gaussian mixture model

GNDVI: Green normalized difference vegetation index

HOG: Histograms of oriented gradients

HSV: Hue saturation value

IO: Input output

KNN: K nearest neighbour

LAI: Leaf area index

LCA: Lignin-Cellulose Absorption Index

LIDAR: Light detection and ranging

LUT: Lookup table

LWVI-1: Normalized Difference Leaf water VI 1

MCARI: Modified Chlorophyll Absorption Ratio Index

MCFI: Multicolour fluorescence imaging

ML: Machine learning

MSR: Modified simple ratio

NDVI: Normalized Difference Vegetation index

NIR: Near infrared

NLI: Nonlinear vegetation index

NTDI: Normalized Tillage Difference Index

ORB: Oriented FAST and rotate

OSAVI: Optimized Soil Adjusted Vegetation Index

PCA: Principal component analysis

PWI: Plant Water Index

QTL: Quantitative trait locus

RDVI: Renormalized differences vegetative index

RGB: Red, green, blue

ROI: Region of interest

SDF: Signed distance function

SIFT: Scale invariant features transforms

SURF: Speeded-up robust features

SVM: Support vector machine

SWIR: Short wave infrared

TDI: Time delay and integration

ToF: Time of flight

VI: Vegetation index

VIS: Visible spectrum

Competing interests

The authors declare they have no competing interests

Funding

This work was funded by grants FEDER BFU-2013-45148-R, Fundación Séneca 19398/PI/14 to MEC and FEDER ViSelTR (TIN2012-39279) to PJN

Availability of supporting data and material

Not applicable

Authors contributions

FPS, MEC and PJN defined the scope of the manuscript, FPS, MEC and PJN wrote and corrected the manuscript, MEC and PJN wrote the grant applications.

Bibliography

1. Tucker C. Red and photographic infrared linear combinations for monitoring vegetation. Remote Sens. Environ. [Internet]. 1979 [cited 2016 Oct 11]; Available from: http://www.sciencedirect.com/science/article/pii/0034425779900130

2. DeFries R, Townshend J. NDVI-derived land cover classifications at a global scale. Int. J. Remote [Internet]. 1994 [cited 2016 Oct 11]; Available from: http://www.tandfonline.com/doi/abs/10.1080/01431169408954345

3. Pettorelli N, Vik J, Mysterud A, Gaillard J. Using the satellite-derived NDVI to assess ecological responses to environmental change. Trends Ecol. [Internet]. 2005 [cited 2016 Oct 11]; Available from: http://www.sciencedirect.com/science/article/pii/S016953470500162X

4. Mkhabela MS, Bullock P, Raj S, Wang S, Yang Y. Crop yield forecasting on the Canadian Prairies using MODIS NDVI data. Agric. For. Meteorol. 2011;151:385–93.

5. GROTEN SME. NDVI—crop monitoring and early yield assessment of Burkina Faso. Int. J. Remote Sens. [Internet]. Taylor & Francis Group ; 1993 [cited 2016 Dec 6];14:1495–515. Available from:

http://www.tandfonline.com/doi/abs/10.1080/01431169308953983

6. Jones HG, Stoll M, Santos T, de Sousa C, Chaves MM, Grant OM. Use of infrared thermography for monitoring stomatal closure in the field: application to grapevine. J. Exp. Bot. [Internet]. Oxford University Press; 2002 [cited 2016 Dec 6];53:2249–60. Available from: http://www.ncbi.nlm.nih.gov/pubmed/12379792

7. Chaerle L, Van der Straeten D. Seeing is believing: imaging techniques to monitor plant health. Biochim. Biophys. Acta-Gene Struct. Expr. 2001;1519:153–66.

8. Sirault XRR, James RA, Furbank RT, Bernstein L, Hayward H, Flowers T, et al. A new screening method for osmotic component of salinity tolerance in cereals using infrared thermography. Funct. Plant Biol. [Internet]. CSIRO PUBLISHING; 2009 [cited 2016 Dec 6];36:970. Available from: http://www.publish.csiro.au/?paper=FP09182

9. Lobet G, Pagès L, Draye X. A Novel Image Analysis Toolbox Enabling Quantitative Analysis of Root System Architecture. Plant Physiol. [Internet]. 2011;157:29–39. Available from: http://www.ncbi.nlm.nih.gov/pubmed/21771915

10. Galkovskyi T, Mileyko Y, Bucksch A, Moore B, Symonova O, Price CA, et al. GiA Roots: software for the high throughput analysis of plant root system architecture. BMC Plant Biol. [Internet]. BioMed Central; 2012 [cited 2016 Sep 10];12:116. Available from: http://bmcplantbiol.biomedcentral.com/articles/10.1186/1471-2229-12-116

11. French A, Ubeda-Tomas S, Holman TJ, Bennett MJ, Pridmore T. High-Throughput Quantification of Root Growth Using a Novel Image-Analysis Tool. Plant Physiol. [Internet]. American Society of Plant Biologists; 2009 [cited 2016 Sep 20];150:1784–95. Available from: http://www.pubmedcentral.nih.gov/articlerender.fcgi?artid=2719150&tool=pmcentrez&rendertype=abstract

12. Golzarian MR, Frick RA, Rajendran K, Berger B, Roy S, Tester M, et al. Accurate inference of shoot biomass from high-throughput images of cereal plants. Plant Methods [Internet]. BioMed Central; 2011 [cited 2016 Sep 10];7:2. Available from: http://plantmethods.biomedcentral.com/articles/10.1186/1746-4811-7-2

13. Fabre J, Dauzat M, Negre V, Wuyts N, Tireau A, Gennari E, et al. PHENOPSIS DB: an Information System for Arabidopsis thaliana phenotypic data in an environmental context. BMC Plant Biol. 2011;11.

14. Araus JL, Cairns JE. Field high-throughput phenotyping: The new crop breeding

frontier. Trends Plant Sci. 2014;19:52–61.

15. Furbank RT. Plant phenomics: from gene to form and function. Funct. Plant Biol. [Internet]. 2009 [cited 2016 Aug 31];36:V–Vi. Available from: http://citeseerx.ist.psu.edu/viewdoc/download?doi=10.1.1.547.5673&rep=rep1&type=pdf

16. Poorter H, Fiorani F, Pieruschka R, Putten WH Van Der, Kleyer M, Schurr U. Tansley review Pampered inside , pestered outside ? Differences and similarities between plants growing in controlled conditions and in the field. New Phytol. 2016;838–55.

17. Yang W, Duan L, Chen G, Xiong L, Liu Q. Plant phenomics and high-throughput phenotyping: Accelerating rice functional genomics using multidisciplinary technologies. Curr. Opin. Plant Biol. [Internet]. Elsevier Ltd; 2013;16:180–7. Available from: http://dx.doi.org/10.1016/j.pbi.2013.03.005

18. White J, Andrade-Sanchez P, Gore M. Field-based phenomics for plant genetics research. F. Crop. [Internet]. 2012 [cited 2016 Aug 31]; Available from: http://www.sciencedirect.com/science/article/pii/S037842901200130X

19. Fahlgren N, Gehan MA, Baxter I. Lights, camera, action: High-throughput plant phenotyping is ready for a close-up. Curr. Opin. Plant Biol. 2015. p. 93–9.

20. Furbank RT, Tester M. Phenomics - technologies to relieve the phenotyping bottleneck. Trends Plant Sci. 2011;16:635–44.

21. Granier C, Vile D. Phenotyping and beyond: Modelling the relationships between traits. Curr. Opin. Plant Biol. [Internet]. Elsevier Ltd; 2014;18:96–102. Available from: http://dx.doi.org/10.1016/j.pbi.2014.02.009

22. Xiong L, David L, Stevenson B, Zhu J-K. High Throughput Screening of Signal Transduction Mutants With Luciferase Imaging. Plant Mol. Biol. Report. [Internet]. Kluwer Academic Publishers; 1999 [cited 2016 Sep 10];17:159–70. Available from: http://link.springer.com/10.1023/A:1007519200505

23. Millar AJ, Carré IA, Strayer CA, Chua NH, Kay SA. Circadian clock mutants in Arabidopsis identified by luciferase imaging. Science [Internet]. 1995 [cited 2016 Sep 20];267:1161–3. Available from: http://www.ncbi.nlm.nih.gov/pubmed/7855595

24. Leister D, Varotto C, Pesaresi P, Niwergall A, Salamini F. Large-scale evaluation of plant growth in Arabidopsis thaliana by non-invasive image analysis. Plant Physiol. Biochem. 1999;37:671–8.

25. Matsuo T, Okamoto K, Onai K, Niwa Y, Shimogawara K, Ishiura M. A systematic forward genetic analysis identified components of the Chlamydomonas circadian system. Genes Dev. 2008;22:918–30.

26. Aoki S, Kato S, Ichikawa K, Shimizu M. Circadian expression of the PpLhcb2 gene encoding a major light-harvesting chlorophyll a/b-binding protein in the moss Physcomitrella patens. Plant Cell Physiol. 2004;45:68–76.

27. Kanno T, Lin WD, Fu JL, Wu MT, Yang HW, Lin SS, et al. Identification of coilin mutants in a screen for enhanced expression of an alternatively spliced GFP reporter gene in Arabidopsis thaliana. Genetics. 2016;203:1709–20.

28. Tõth R, Gerding-Reimers C, Deeks MJ, Menninger S, Gallegos RM, Tonaco IAN, et al. Prieurianin/endosidin 1 is an actin-stabilizing small molecule identified from a chemical genetic screen for circadian clock effectors in Arabidopsis thaliana. Plant J. 2012;71:338–52.

29. Teledyne Dalsa. No Title. https://www.teledynedalsa.com/corp/.

30. Van Der Heijden G, Song Y, Horgan G, Polder G, Dieleman A, Bink M, et al. SPICY: Towards automated phenotyping of large pepper plants in the greenhouse. Funct. Plant Biol. CSIRO PUBLISHING; 2012;39:870–7.

31. Navarro PJ, Fernández C, Weiss J, Egea-Cortines M. Development of a configurable growth chamber with a computer vision system to study circadian rhythm in plants. Sensors (Basel). [Internet]. 2012 [cited 2016 Sep 20];12:15356–75. Available from: http://www.ncbi.nlm.nih.gov/pubmed/23202214

32. Zhang X, Huang C, Wu D, Qiao F, Li W, Duan L, et al. High-throughput phenotyping and QTL mapping reveals the genetic architecture of maize plant growth. Plant Physiol. [Internet]. 2017;pp.01516.2016. Available from: http://www.plantphysiol.org/lookup/doi/10.1104/pp.16.01516

33. Biskup B, Scharr H, Schurr U, Rascher U. A stereo imaging system for measuring structural parameters of plant canopies. Plant, Cell Environ. [Internet]. Blackwell Publishing Ltd; 2007 [cited 2016 Sep 20];30:1299–308. Available from: http://doi.wiley.com/10.1111/j.1365-3040.2007.01702.x

34. Song Y, Glasbey CA, van der Heijden GWAM, Polder G, Dieleman JA. Combining Stereo and Time-of-Flight Images with Application to Automatic Plant Phenotyping. Springer Berlin Heidelberg; 2011. p. 467–78.

35. Nguyen TT, Slaughter DC, Max N, Maloof JN, Sinha N. Structured light-based 3D reconstruction system for plants. Sensors (Switzerland) [Internet]. Multidisciplinary Digital Publishing Institute; 2015 [cited 2016 Sep 21];15:18587–612. Available from: http://www.mdpi.com/1424-8220/15/8/18587/

36. Klodt M, Herzog K, Töpfer R, Cremers D, Töpfer R, Hausmann L, et al. Field phenotyping of grapevine growth using dense stereo reconstruction. BMC Bioinformatics [Internet]. BioMed Central; 2015 [cited 2016 Oct 7];16:143. Available from: http://www.biomedcentral.com/1471-2105/16/143

37. Xiong X, Yu L, Yang W, Liu M, Jiang N, Wu D, et al. A high-throughput stereo-imaging system for quantifying rape leaf traits during the seedling stage. Plant Methods [Internet]. BioMed Central; 2017 [cited 2017 Feb 8];13:7. Available from: http://plantmethods.biomedcentral.com/articles/10.1186/s13007-017-0157-7

38. Paproki A, Sirault XRR, Berry S, Furbank RT, Fripp J. A novel mesh processing based technique for 3D plant analysis. BMC Plant Biol. [Internet]. 2012;12:63. Available from: http://www.biomedcentral.com/1471-2229/12/63

39. Kise M, Park B, Heitschmidt GW, Lawrence KC, Windham WR. Multispectral imaging system with interchangeable filter design. Comput. Electron. Agric. 2010;72:61–8.

40. Li P, Lee S-H, Hsu H-Y, Park J-S. Nonlinear Fusion of Multispectral Citrus Fruit Image Data with Information Contents. Sensors [Internet]. Multidisciplinary Digital Publishing Institute; 2017 [cited 2017 Jan 24];17:142. Available from: http://www.mdpi.com/1424-8220/17/1/142

41. Wahabzada M, Mahlein A-K, Bauckhage C, Steiner U, Oerke E-C, Kersting K. Plant Phenotyping using Probabilistic Topic Models: Uncovering the Hyperspectral Language of Plants. Sci. Rep. [Internet]. Nature Publishing Group; 2016 [cited 2017 Jan 24];6:22482. Available from: http://www.ncbi.nlm.nih.gov/pubmed/26957018

42. Navarro P, Fernández C, Borraz R, Alonso D. A Machine Learning Approach to Pedestrian Detection for Autonomous Vehicles Using High-Definition 3D Range Data. Sensors [Internet]. Multidisciplinary Digital Publishing Institute; 2016 [cited 2017 Jan 24];17:18. Available from: http://www.mdpi.com/1424-8220/17/1/18

43. Eitel JUH, Höfle B, Vierling LA, Abellán A, Asner GP, Deems JS, et al. Beyond 3-D: The new spectrum of lidar applications for earth and ecological sciences. Remote Sens.

Environ. 2016;186:372–92.

44. Costa JM, Grant OM, Chaves MM, I D, M F, RD J, et al. Thermography to explore plant-environment interactions. J. Exp. Bot. [Internet]. Oxford University Press; 2013 [cited 2017 Jan 24];64:3937–49. Available from: https://academic.oup.com/jxb/article-lookup/doi/10.1093/jxb/ert029

45. Heisel F, Sowinska M, Miehé JA, Lang M, Lichtenthaler HK. Detection of Nutrient Deficiencies of Maize by Laser Induced Fluorescence Imaging. J. Plant Physiol. 1996;148:622–31.

46. Saito Y, Kanoh M, Hatake K, Kawahara TD, Nomura A. Investigation of laser-induced fluorescence of several natural leaves for application to lidar vegetation monitoring. Appl. Opt. [Internet]. 1998 [cited 2017 Jan 24];37:431–7. Available from: http://www.ncbi.nlm.nih.gov/pubmed/18268602

47. Latouche G, Debord C, Raynal M, Milhade C, Cerovic ZG. First detection of the presence of naturally occurring grapevine downy mildew in the field by a fluorescence-based method. Photochem. Photobiol. Sci. [Internet]. 2015 [cited 2017 Jan 24];14:1807–13. Available from: http://www.ncbi.nlm.nih.gov/pubmed/26293623

48. Pérez-Bueno ML, Pineda M, Cabeza FM, Barón M. Multicolor Fluorescence Imaging as a Candidate for Disease Detection in Plant Phenotyping. Front. Plant Sci. [Internet]. Frontiers Media SA; 2016 [cited 2017 Jan 24];7:1790. Available from: http://www.ncbi.nlm.nih.gov/pubmed/27994607

49. Jan Erik Solem. Programming Computer Vision with Python. Andy Oram, Mike Hendrikosn, editors. Program. Comput. Vis. with Python [Internet]. 1st ed. Sebastopol: O'Reilly Media; 2012;264. Available from: http://programmingcomputervision.com/

50. Navarro PJ, Pérez F, Weiss J, Egea-Cortines M. Machine learning and computer vision system for phenotype data acquisition and analysis in plants. Sensors (Switzerland) [Internet]. Multidisciplinary Digital Publishing Institute; 2016 [cited 2016 Sep 21];16:1–12. Available from: http://www.mdpi.com/1424-8220/16/5/641

51. Sonka M, Hlavac V, Boyle R. Image Processing, Analysis, and Machine Vision. 3rd ed. Thomson, editor. Toronto: Thomson; 2008.

52. Haboudane D. Hyperspectral vegetation indices and novel algorithms for predicting green LAI of crop canopies: Modeling and validation in the context of precision agriculture. Remote Sens. Environ. [Internet]. 2004 [cited 2016 Sep 26];90:337–52.

Available from: http://linkinghub.elsevier.com/retrieve/pii/S0034425704000264

53. Walter A, Liebisch F, Hund A, Johannsen W, Johannsen W, Schulze W, et al. Plant phenotyping: from bean weighing to image analysis. Plant Methods [Internet]. BioMed Central; 2015 [cited 2016 Sep 26];11:14. Available from: http://www.plantmethods.com/content/11/1/14

54. Rocha A V., Shaver GR. Advantages of a two band EVI calculated from solar and photosynthetically active radiation fluxes. Agric. For. Meteorol. [Internet]. 2009 [cited 2016 Sep 26];149:1560–3. Available from: http://linkinghub.elsevier.com/retrieve/pii/S0168192309000860

55. Hartmann A, Czauderna T, Hoffmann R, Stein N, Schreiber F, Granier C, et al. HTPheno: An image analysis pipeline for high-throughput plant phenotyping. BMC Bioinformatics [Internet]. BioMed Central; 2011 [cited 2016 Sep 10];12:148. Available from: http://bmcbioinformatics.biomedcentral.com/articles/10.1186/1471-2105-12-148

56. Klodt M, Herzog K, Töpfer R, Cremers D, Töpfer R, Hausmann L, et al. Field phenotyping of grapevine growth using dense stereo reconstruction. BMC Bioinformatics. BioMed Central; 2015;16:143.

57. Darwin CR. On the movements and habits of climbing plants. J. Linn. Soc. London. 1865;9:1–118.

58. Someya N, Niinuma K, Kimura M, Yamaguchi I, Hamamoto H. Circumnutation of Arabidopsis thaliana inflorescence stems. Biol. Plant. 2006;50:287–90.

59. Johnsson A, Solheim BGB, Iversen TH. Gravity amplifies and microgravity decreases circumnutations in Arabidopsis thaliana stems: results from a space experiment. New Phytol. 2009;182:621–9.

60. Biskup B, Scharr H, Fischbach A, Wiese-Klinkenberg A, Schurr U, Walter A. Diel Growth Cycle of Isolated Leaf Discs Analyzed with a Novel, High-Throughput Three-Dimensional Imaging Method Is Identical to That of Intact Leaves. Plant Physiol. 2009;149:1452–61.

61. Lenhard M, Laux T. Shoot meristem formation and maintenance. Curr. Opin. Plant Biol. 1999;2:44–50.

62. Courbon J, Mezouar Y, Guénard N. Vision-based navigation of unmanned aerial vehicles. Control Eng. [Internet]. 2010 [cited 2016 Sep 12]; Available from:

http://www.sciencedirect.com/science/article/pii/S0967066110000808

63. Gonzalez L, Montes G, Puig E, Johnson S. Unmanned Aerial Vehicles (UAVs) and artificial intelligence revolutionizing wildlife monitoring and conservation. Sensors [Internet]. 2016 [cited 2016 Sep 12]; Available from: http://www.mdpi.com/1424-8220/16/1/97

64. Humplík JF, Lazár D, Husičková A, Spíchal L. Automated phenotyping of plant shoots using imaging methods for analysis of plant stress responses - a review. Plant Methods [Internet]. BioMed Central; 2015 [cited 2016 Sep 21];11:29. Available from: http://www.plantmethods.com/content/11/1/29

65. Kim DM, Zhang H, Zhou H, Du T, Wu Q, Mockler TC, et al. Highly sensitive image-derived indices of water-stressed plants using hyperspectral imaging in SWIR and histogram analysis. Sci. Rep. [Internet]. 2015 [cited 2016 Sep 26];5:15919. Available from: http://digitalcommons.wustl.edu/open_access_pubs/4377

66. Phadikar S, Goswami J. Vegetation indices based segmentation for automatic classification of brown spot and blast diseases of rice. 2016 3rd Int. Conf. Recent Adv. Inf. Technol. [Internet]. IEEE; 2016 [cited 2016 Sep 28]. p. 284–9. Available from: http://ieeexplore.ieee.org/lpdocs/epic03/wrapper.htm?arnumber=7507917

67. Wahabzada M, Mahlein A-K, Bauckhage C, Steiner U, Oerke E-C, Kersting K. Plant Phenotyping using Probabilistic Topic Models: Uncovering the Hyperspectral Language of Plants. Sci. Rep. [Internet]. Nature Publishing Group; 2016 [cited 2016 Oct 18];6:22482. Available from: http://www.nature.com/articles/srep22482

68. Krig S. Computer vision metrics: Survey, Taxonomy, and Analysis. Weiss S, Douglas S, editors. ApressOpen; 2014.

69. De Vylder J, Vandenbussche F, Hu Y, Philips W, Van Der Straeten D. Rosette tracker: an open source image analysis tool for automatic quantification of genotype effects. Plant Physiol. [Internet]. American Society of Plant Biologists; 2012 [cited 2016 Nov 2];160:1149–59. Available from: http://www.ncbi.nlm.nih.gov/pubmed/22942389

70. Arvidsson S, Pérez-Rodríguez P, Mueller-Roeber B. A growth phenotyping pipeline for Arabidopsis thaliana integrating image analysis and rosette area modeling for robust quantification of genotype effects. New Phytol. 2011;191:895–907.

71. Clément A, Vigouroux B. Unsupervised segmentation of scenes containing vegetation (Forsythia) and soil by hierarchical analysis of bi-dimensional histograms.

Pattern Recognit. Lett. 2003.

72. Lee WS, Slaughter DC. Recognition of partially occluded plant leaves using a modified watershed algorithm. Trans. ASAE. American Society of Agriculltural Engineers, ASAE; 2004;47:1269–80.

73. Vukadinovic D, Polder G. Watershed and supervised classification based fully automated method for separate leaf segmentation. COST FA 1306 -The quest Toler. Var. plant Cell. Lev. Gatersleben; 2015.

74. Rother C, Kolmogorov V, Blake A. GrabCut -Interactive Foreground Extraction using Iterated Graph Cuts. ACM Trans. Graph. [Internet]. 2004. Available from: https://www.microsoft.com/en-us/research/publication/grabcut-interactive-foreground-extraction-using-iterated-graph-cuts/

75. Boykov YY, Jolly M-P. Interactive graph cuts for optimal boundary & region segmentation of objects in N-D images. Proc. Eighth IEEE Int. Conf. Comput. Vision. ICCV 2001 [Internet]. IEEE Comput. Soc; 2001 [cited 2016 Nov 3]. p. 105–12. Available from: http://ieeexplore.ieee.org/document/937505/

76. Wang X. The GrabCut Segmentation Technique as Used in the Study of Tree Image Extraction. In: Zhu FG and X, editor. Proc. 2009 Int. Work. Inf. Secur. Appl. (IWISA 2009). Qingdao, China: Academy Publisher; 2009.

77. Kass M, Witkin A, Terzopoulos D. Snakes: Active contour models. Int. J. Comput. Vis. [Internet]. Kluwer Academic Publishers; 1988 [cited 2016 Nov 5];1:321–31. Available from: http://link.springer.com/10.1007/BF00133570

78. Minervini M, Abdelsamea MM, Tsaftaris SA. Image-based plant phenotyping with incremental learning and active contours. Ecol. Inform. 2014;23:35–48.

79. Suta L, Bessy F, Veja C, Vaida M-F. Active contours: Application to plant recognition. 2012 IEEE 8th Int. Conf. Intell. Comput. Commun. Process. [Internet]. IEEE; 2012 [cited 2016 Nov 5]. p. 181–7. Available from: http://ieeexplore.ieee.org/document/6356183/

80. Shi Y, Karl WC. A real-time algorithm for the approximation of level-set-based curve evolution. IEEE Trans. Image Process. [Internet]. 2008 [cited 2016 Nov 5];17:645–56. Available from: http://ieeexplore.ieee.org/document/4480128/

81. Chan TF, Vese LA. Active contours without edges. IEEE Trans. Image Process. [Internet]. IEEE Press; 2001 [cited 2016 Nov 5];10:266–77. Available from:

http://ieeexplore.ieee.org/document/902291/

82. Peng W, Wenlin L, Wenlong S. Segmentation of Leaf Images Based on the Active Contours. Int. J. Sci. Technol. [Internet]. 2015 [cited 2016 Nov 5];8:63–70. Available from: http://dx.doi.org/10.14257/ijunesst.2015.8.6.07

83. Lowe DG. Distinctive Image Features from Scale-Invariant Keypoints. Int. J. Comput. Vis. [Internet]. Kluwer Academic Publishers; 2004 [cited 2016 Dec 7];60:91–110. Available from: http://link.springer.com/10.1023/B:VISI.0000029664.99615.94

84. Bay H, Ess A, Tuytelaars T, Van Gool L. Speeded-Up Robust Features (SURF). Comput. Vis. Image Underst. 2008;110:346–59.

85. Panchal PM, Panchal SR, Shah SK. A Comparison of SIFT and SURF. ISSN Int. J. Innov. Res. Comput. Commun. Eng. [Internet]. 2013 [cited 2016 Dec 7];1:2320–9798. Available from: www.ijircce.com

86. Calonder M, Lepetit V, Strecha C, Fua P. BRIEF: Binary Robust Independent Elementary Features. 2010 [cited 2016 Dec 7]. p. 778–92. Available from: http://link.springer.com/10.1007/978-3-642-15561-1_56

87. Rublee E, Rabaud V, Konolige K, Bradski G. ORB: An efficient alternative to SIFT or SURF. 2011 Int. Conf. Comput. Vis. [Internet]. IEEE; 2011 [cited 2016 Dec 12]. p. 2564–71. Available from: http://ieeexplore.ieee.org/document/6126544/

88. Rosten E, Drummond T. Machine Learning for High-Speed Corner Detection. Springer Berlin Heidelberg; 2006 [cited 2016 Dec 8]. p. 430–43. Available from: http://link.springer.com/10.1007/11744023_34

89. Karami E, Prasad S, Shehata M. Image Matching Using SIFT , SURF , BRIEF and ORB : Performance Comparison for Distorted Images Image Matching Using SIFT , SURF , BRIEF and ORB : Performance Comparison for Distorted Images. 2015;

90. Dalal N, Triggs B. Histograms of Oriented Gradients for Human Detection. 2005 IEEE Comput. Soc. Conf. Comput. Vis. Pattern Recognit. [Internet]. IEEE; [cited 2016 Dec 9]. p. 886–93. Available from: http://ieeexplore.ieee.org/document/1467360/

91. Santos T, Oliveira A. Image-based 3D digitizing for plant architecture analysis and phenotyping. … SIBGRAPI 2012 (XXV Conf. … [Internet]. 2012 [cited 2016 Dec 8]; Available from: http://www.cnptia.embrapa.br/~thiago/pool/2012-08-24_sibgrapi.pdf

92. Lou L, Liu Y, Sheng M, Han J, Doonan JH. A Cost-Effective Automatic 3D Reconstruction Pipeline for Plants Using Multi-view Images. 2014 [cited 2016 Dec 8]. p.

221–30. Available from: http://link.springer.com/10.1007/978-3-319-10401-0_20

93. Pound MP, French AP, Fozard JA, Murchie EH, Pridmore TP. A patch-based approach to 3D plant shoot phenotyping. Mach. Vis. Appl. [Internet]. Springer Berlin Heidelberg; 2016 [cited 2016 Dec 9];27:767–79. Available from: http://link.springer.com/10.1007/s00138-016-0756-8

94. Lantz B. Machine Learning with R. 1st ed. Jones J, Sheikh A, editors. Birmingham: Packt Publishing; 2013.

95. Müller A, Guido S. Introduction to Machine Learning with Python. 1st ed. Schanafelt D, editor. Sebastopol: O'Reilly Media; 2016.

96. Smola A, Vishwanathan SV. Introduction to Machine Learning. 1st ed. Cambridge: Cambridge University Press; 2008.

97. Singh A, Ganapathysubramanian B, Singh AK, Sarkar S. Machine Learning for High-Throughput Stress Phenotyping in Plants. Trends Plant Sci. 2016. p. 110–24.

98. Pape J-M, Klukas C. Utilizing machine learning approaches to improve the prediction of leaf counts and individual leaf segmentation of rosette plant images. Proc. Comput. Vis. Probl. Plant Phenotyping [Internet]. British Machine Vision Association; 2015 [cited 2016 Sep 21];1–12. Available from: http://www.bmva.org/bmvc/2015/cvppp/papers/paper003/index.html

99. Baranowski P, Jedryczka M, Mazurek W, Babula-Skowronska D, Siedliska A, Kaczmarek J, et al. Hyperspectral and Thermal Imaging of Oilseed Rape (Brassica napus) Response to Fungal Species of the Genus Alternaria. Wilson RA, editor. PLoS One [Internet]. Public Library of Science; 2015 [cited 2016 Nov 6];10:e0122913. Available from: http://dx.plos.org/10.1371/journal.pone.0122913

100. Fukushima K. Neocognitron: a self organizing neural network model for a mechanism of pattern recognition unaffected by shift in position. Biol. Cybern. [Internet]. 1980 [cited 2016 Nov 6];36:193–202. Available from: http://www.ncbi.nlm.nih.gov/pubmed/7370364

101. Pound MP, Burgess AJ, Wilson MH, Atkinson JA, Griffiths M, Jackson AS, et al. Deep Machine Learning provides state-of-the-art performance in image-based plant phenotyping. bioRxiv [Internet]. 2016 [cited 2016 Sep 21];53033. Available from: http://biorxiv.org/lookup/doi/10.1101/053033

102. Mohanty SP, Hughes D, Salathé M. Using Deep Learning for Image-Based Plant

Disease Detection. 2016;1–7.

103. Krizhevsky A, Sutskever I, Hinton GE. ImageNet Classification with Deep Convolutional Neural Networks. Pereira F, Burges CJC, Bottou L, editors. Adv. Neural Inf. Process. Syst. 2012;1–9.

104. Szegedy C, Liu W, Jia Y, Sermanet P, Reed S, Anguelov D, et al. Going deeper with convolutions. Proc. IEEE Comput. Soc. Conf. Comput. Vis. Pattern Recognit. [Internet]. 2015 [cited 2016 Nov 7]. p. 1–9. Available from: http://arxiv.org/abs/1409.4842

105. Hughes D, Marcel Salathe. An open access repository of images on plant health to enable the development of mobile disease diagnostics [Internet]. 2015 Nov. Available from: http://arxiv.org/abs/1511.08060

106. Nagler PL, Inoue Y, Glenn E., Russ A., Daughtry CS. Cellulose absorption index (CAI) to quantify mixed soil–plant litter scenes. Remote Sens. Environ. 2003;87:310–25.

107. Ren H, Zhou G, Zhang F, Zhang X. Evaluating cellulose absorption index (CAI) for non-photosynthetic biomass estimation in the desert steppe of Inner Mongolia. Chinese Sci. Bull. SP Science China Press; 2012;57:1716–22.

108. Serbin G, Daughtry CST, Hunt ER, Reeves JB, Brown DJ. Effects of soil composition and mineralogy on remote sensing of crop residue cover. Remote Sens. Environ. 2009;113:224–38.

109. Eskandari I, Navid H, Rangzan K. Evaluating spectral indices for determining conservation and conventional tillage systems in a vetch-wheat rotation. Int. Soil Water Conserv. Res. 2016;4:93–8.

110. Galvão LS, Formaggio AR, Tisot DA. Discrimination of sugarcane varieties in Southeastern Brazil with EO-1 Hyperion data. Remote Sens. Environ. 2005;94:523–34.

111. Price J. Leaf area index estimation from visible and near-infrared reflectance data. Remote Sens. Environ. 1995;52:55–65.

112. Zarco-Tejada P, Berjón A, Miller J. Stress detection in crops with hyperspectral remote sensing and physical simulation models. Proc. Airborne. 2004;

113. Cai J, Golzarian MR, Miklavcic SJ. Novel Image Segmentation Based on Machine Learning and Its Application to Plant Analysis. Int. J. Inf. Electron. Eng. 2011;1.

114. Gong P, Pu R, Biging G. Estimation of forest leaf area index using vegetation indices derived from Hyperion hyperspectral data. IEEE Trans. 2003;

115. Brown HE, Diuk-Wasser MA, Guan Y, Caskey S, Fish D. Comparison of three satellite sensors at three spatial scales to predict larval mosquito presence in Connecticut wetlands. Remote Sens. Environ. 2008;112:2301–8.

116. Apan A, Held A, Phinn S, Markley J. Detecting sugarcane “orange rust” disease using EO-1 Hyperion hyperspectral imagery. Int. J. Remote Sens. Taylor & Francis Group ; 2004;25:489–98.

117. Tucker CJ, Slayback DA, Pinzon JE, Los SO, Myneni RB, Taylor MG. Higher northern latitude normalized difference vegetation index and growing season trends from 1982 to 1999. Int. J. Biometeorol. Springer-Verlag; 2001;45:184–90.

118. Haboudane D, Miller JR, Pattey E, Zarco-Tejada PJ, Strachan IB. Hyperspectral vegetation indices and novel algorithms for predicting green LAI of crop canopies: Modeling and validation in the context of precision agriculture. Remote Sens. Environ. 2004;90:337–52.

119. Pagani A, Echeverría HE, Andrade FH, Sainz Rozas HR. Characterization of Corn Nitrogen Status with a Greenness Index under Different Availability of Sulfur. Agron. J. American Society of Agronomy; 2009;101:315.

120. Blackburn GA. Spectral indices for estimating photosynthetic pigment concentrations: A test using senescent tree leaves. Int. J. Remote Sens. Taylor & Francis Group ; 1998;19:657–75.

121. Hunt, Jr. ER, Hively WD, Fujikawa SJ, Linden DS, Daughtry CST, McCarty GW. Acquisition of NIR-Green-Blue Digital Photographs from Unmanned Aircraft for Crop Monitoring. Remote Sens. Molecular Diversity Preservation International; 2010;2:290–305.

122. Bell GE, Howell BM, Johnson GV, Raun WR, Solie JB, Stone ML. Optical Sensing of Turfgrass Chlorophyll Content and Tissue Nitrogen. HortScience. American Society for Horticultural Science; 2004;39:1130–2.

123. Haboudane D, Miller JR, Tremblay N, Zarco-Tejada PJ, Dextraze L. Integrated narrow-band vegetation indices for prediction of crop chlorophyll content for application to precision agriculture. Remote Sens. Environ. 2002;81:416–26.

124. Timm BC, McGarigal K. Fine-scale remotely-sensed cover mapping of coastal dune and salt marsh ecosystems at Cape Cod National Seashore using Random Forests. Remote Sens. Environ. 2012;127:106–17.

125. Parenteau MP, Bannari A, El-Harti A, Bachaoui M, El-Ghmari A. Characterization of the state of soil degradation by erosion using the hue and coloration indices. IGARSS 2003. 2003 IEEE Int. Geosci. Remote Sens. Symp. Proc. (IEEE Cat. No.03CH37477). IEEE; p. 2284–6.

Tables

Table 1 A list of indexes, the corresponding wavelength ranges and their use to analyse plant material.

Index	Range nm	Applications
**CAI** – Cellulose Absorption Index	2200-2000	Quantification mixed soil–plant litter scenes [106], estimation of non-photosynthetic biomass [107]
**LCA** – Lignin-Cellulose Absorption Index	2365-2145	Measure the effects of soil composition and mineralogy of crop residue cover [108]
**NTDI** – Normalized Difference Tillage Index	2359-1150	Used for identifying crop residue cover in conventional and conservation tillage systems [109]
**LWVI-1** – Normalized Difference Leaf water VI 2	1094-893	Discrimination of sugarcane varieties, allowed to detect large amounts of non photosynthetically-active constituents within the canopy [110]
**DLAI** – Difference Leaf Area Index	1725-970	Used for estimating leaf area index based on the radiation measurements in the visible and near-infrared [111]
**PWI** – Plant Water Index	970-902	Water content estimation and study of the characteristics of canopy spectrum and growth status [112][113]
**NLI** – Nonlinear vegetation index	1400-780	Measurement of plant leaf water content. In combination with others indexes can detect interaction of biochemicals such as protein, nitrogen, lignin, cellulose, sugar, and starch [114]
**DWSI** – Disease water stress index	1657-547	To predict larval mosquito presence in wetland [115]and detect sugarcane 'orange rust' disease [116]
**NDVI** – Normalized Difference Vegetation Index	800-670	Measurement significant variations in photosynthetic activity and growing season length at different latitudes [117]
**MCARI** – Modified Chlorophyll Absorption Ratio Index	700-670	Study of vegetation biophysical parameters, as well as to external factors affecting canopy reflectance [118]
**GI** – Greenness Index	670-550	Characterization of corn nitrogen status [119]
**CAR** – Chlorophyll absorption ratio	700-500	Estimating the concentration of individual photosynthetic pigments within vegetation [120]
**GNDVI** – Green normalized difference vegetation index	800-550	Providing important information for site-specific agricultural decision making [121] and for identification of chlorophyll content and tissue nitrogen [122]
**OSAVI** – Optimized Soil Adjusted Vegetation Index	800-670	Measurement with high sensitive of chlorophyll content variations and very resistant to the variations of LAI and solar zenith angle [123]
**CI r** – Coloration Index red	780-710	Mapping of coastal dune and salt marsh ecosystems [124]
**CI g** – Coloration Index green	780-550	Characterization of the state of soil degradation by erosion [125]

Table 2 List of Artificial Vision software libraries and their languages

Vision libraries	Source	Language
OpenCV	http://opencv.org	C++, Python, Java
PlantCV<br>Scikit-image	*http://plantcv.danforthcenter.org*<br>*http://scikit-image.org*	Python
Bioimagetools, bayesimages, edci, DRIP, dpmixsim, raster, ...	https://cran.r-project.org/	R
Cimg<br>Simplecv<br>Fastcv<br>Ccv<br>Vxl	*http://cimg.eu*<br>*http://*Simplecv.org<br>https://developer.qualcomm.com/software/fastcv-sdk<br>*http://*libccv.org<br>*http://*vxl.sourceforge.net	C++
BoofCV<br>OpenIMAJ<br>JavaCV	http://boofcv.org<br>http://openimaj.org<br>https://github.com/bytedeco/javacv	Java

Table 3 List of Machine Learning software libraries and their languages

**Libraries ML/DL**	**Source**	**Language**
MICE, rpart, Party, CARET, randomForest, nnet, e1071, KernLab, igraph, glmnet, ROCR, tree, Rweka, earth, klaR,	https://cran.r-project.org/	R
Scikit-learn<br>Tensorflow<br>Theano<br>Pylearn2,<br>NuPIC<br>Caffe<br>PyBrain	http://scikit-learn.org/stable/<br>*https://www.tensorflow.org/*<br>*http://deeplearning.net/software/theano*<br>*http://deeplearning.net/software/pylearn2*<br>*http://numenta.org/*<br>*http://caffe.berkeleyvision.org/*<br>*http://pybrain.org/*	Python
Weka<br>Spark<br>Mallet<br>JSAT<br>ELKI<br>Java-ML	http://www.cs.waikato.ac.nz/ml/weka/<br>http://spark.apache.org/<br>http://mallet.cs.umass.edu/<br>https://github.com/EdwardRaff/JSAT<br>http://elki.dbs.ifi.lmu.de/<br>http://java-ml.sourceforge.net/	Java
Multiboost<br>Shogun<br>LibSVM<br>mlpack<br>Shark<br>MLC++	http://www.multiboost.org/<br>http://shogun-toolbox.org/<br>http://www.csie.ntu.edu.tw/~cjlin/libsvm/<br>http://mlpack.org/<br>http://image.diku.dk/shark/<br>http://www.sgi.com/tech/mlc/source.html	C++, C

Figure Legends

Figure 1. Basic workflow in computer vision-based plant phenotyping

Figure 2. An overview of different spectra used for phenotyping and the associated cameras. Names of different indexes are found in Table 1.

Figure Click here to download Figure Figure1_gigascience.pdf

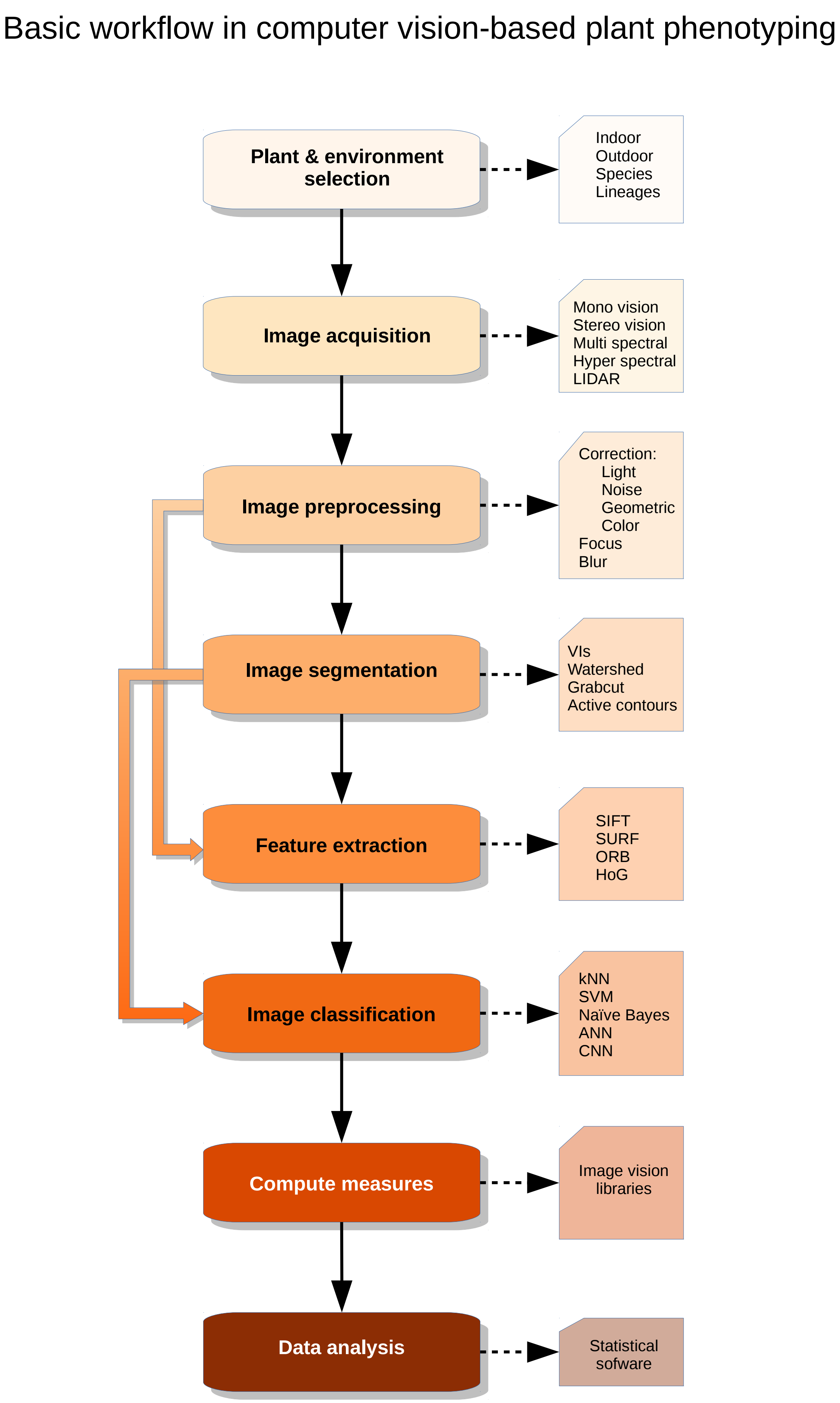

Figure

Click here to download Figure figure2Gigascience.pdf

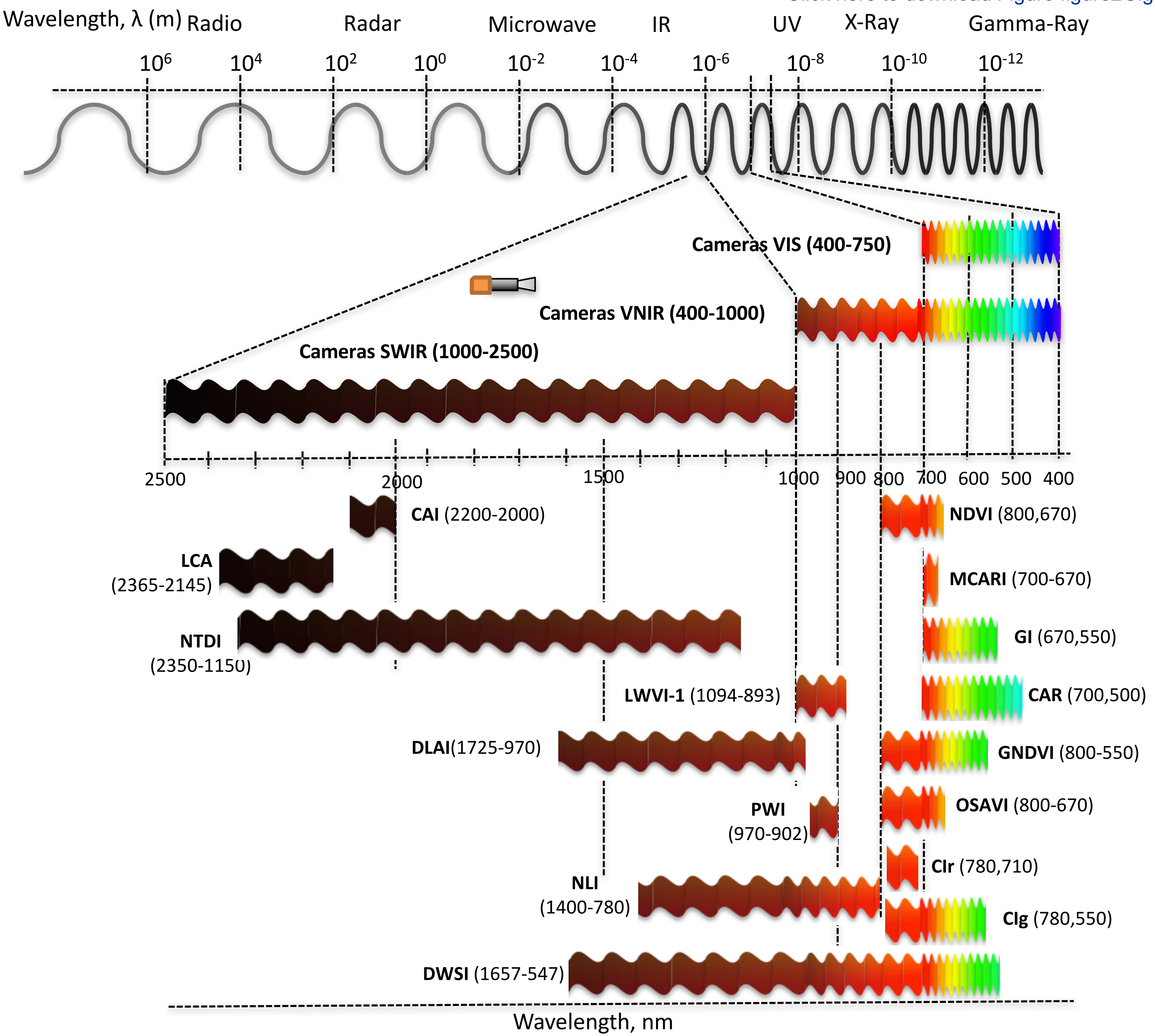

Supplement: GIGA-D-17-00043_Original-Submission.pdf [file gix092_giga-d-17-00043_original-submission.pdf]
