# Supplementary material for: Plant phenomics: an overview of image acquisition technologies and image data analysis algorithms
Source: Gigascience. 2017 Oct 3;6(11):1–18. doi: 10.1093/gigascience/gix092 (PMC5737281; doi:10.1093/gigascience/gix092)

GigaScience

Plant phenomics: an overview of image acquisition technologies and image data analysis algorithms

--Manuscript Draft--

**Manuscript Number:**	GIGA-D-17-00043R2
**Full Title:**	Plant phenomics: an overview of image acquisition technologies and image data analysis algorithms
**Article Type:**	Review
**Funding Information:**	Ministerio de Economía y Competitividad (ES) (BFU-2013-45148-R) — Prof.Dr. Marcos Egea-Cortines<br>Ministerio de Economia y Competitividad (TIN2012-39279) — Prof Pedro Javier Navarro<br>Fundación Séneca (19398/PI/14) — Prof.Dr. Marcos Egea-Cortines
**Abstract:**	The study of phenomes or phenomics has been a central part of biology. The field of automatic phenotype acquisition technologies based on images has seen an important advance in the last years. As other high throughput technologies, it bears from a common set of problems, including data acquisition and analysis. In this review, we give an overview of the main systems developed to acquire images. We give an in-depth analysis of image processing with its major issues, and the algorithms that are being used or emerging as useful to obtain data out of images in an automatic fashion.
**Corresponding Author:**	Marcos Egea-Cortines, PhD<br>Universidad Politecnica de Cartagena<br>Cartagena, Murcia SPAIN
**Corresponding Author Secondary Information:**	
**Corresponding Author's Institution:**	Universidad Politecnica de Cartagena
**Corresponding Author's Secondary Institution:**	
**First Author:**	Fernando Perez-Sanz, MsC
**First Author Secondary Information:**	
**Order of Authors:**	Fernando Perez-Sanz, MsC<br>Pedro Javier Navarro, PhD<br>Marcos Egea-Cortines, PhD
**Order of Authors Secondary Information:**	
**Response to Reviewers:**	Dear Dr. Nogoy,<br>Thanks very much for the opportunity to improve the manuscript. We have met all the requests. We wanted to point out that referee •2 requested a list of advantages and disadvantages for the different types of algorithms used in image analysis. We believe that, while that can be generalized for sensors, it is not the case in image analysis. We argue that the researchers need to test several methods to identify the best for their specific type of image, and forecasting the best outcome is not reasonable.<br>We have written this in the text, and as requested added yet another table.<br>We hope that the work will suffice for publication, waiting of course the veredict of the referees.<br><br>Bests<br>Marcos<br><br>Reviewer reports:<br>Reviewer #1:

Powered by Editorial Manager® and ProduXion Manager® from Aries Systems Corporation

	I would like to thank the authors for the changes they made in the manuscript. I have the feeling it reads much better now and is more accessible to a broader audience.<br><br>I still have a couple of comments/suggestions (minor ones):<br><br><br>Line 49-line 76: I am still not sure to understand why the authors choose to focus this paragraph on the used of gene reporter, which is a very narrow example of the phenotyping today. I would personally remove this section.<br><br>We have removed it<br><br>Line 581: "The majority of the work has been made in indoor-setups ": I am really not sure about that... A lot of tools and methods have been developed recently to work outdoor and into the field.<br><br>We have rephrased it to have a less blunt statement<br><br><br>Reviewer #2: I am appreciated that the authors made big efforts to rewrite this review according to the reviewer's opinions. In recent years, many similar reviews related with Plant Phenomics had been published. And the authors stated that: "a detailed review on different type of data analysis is lacking. In this review, we cover the current and emerging methods of image acquisition and processing allowing image-based phenomics". This review should focus on image data analysis depending on different data type or sensors, which is lacking. Thus, two major issues should be improved and summarized more clearly in this review. Moreover, some previous comments are ignored by the authors or the related sentences had been removed, which should be declared.<br><br>We took into consideration all the previous comments, but as we made a major rewriting, they do not always appear as a word by word answer.<br><br>From the previous version:<br>“We have corrected this (line 222)<br>17.Line 160-163: please add references.<br>18.Line 167-169: please add references.<br>19.Line 173-177: please add references.<br><br>20.Line 178-181: More applications of plant phenotpying with LIDAR in recent years should be cited. Please discuss the disadvantage of the LIDAR.<br>21.Line 177: the end of the sentence lacks punctuation.<br>We have rewritten this whole part and included new references<br>22.Line 186: "14.000 nm" should be change to "14,000 nm". The image which obtained by thermographic camera should include a range of wavelength. Moreover, please add the reference.<br>We have added the reference. We have added fluorescence imaging with the corresponding ranges and references (line 297-322)<br><br>23.Line 196: "as a result of UV light excitation" is not rigorous, and please add the reference.<br>We have rewritten this part (see above line 397-322)<br>24.Line 75-203: more image acquisition techniques, such as x-ray CT, should be added. And the authors should summarize the merit and drawback of these imaging techniques.<br><br>25.Line 227-229: please add the reference of the "In fact, when information is measured as entropy, pre-processing causes a decrease in entropy". Or this is the author's own opinion.<br>We have rewritten this entire section<br>26.Line 235-265: please introduce the procedures of image correction and images enhancement more concisely, and please add the reference.<br>We have rewritten this entire section<br>27.Line 271-272: please add the references to the "Leaf Area Index (LAI), biomass,

	chlorophyll concentration, photosynthetic activity", respectively.<br>28.Line 287: please add the references to the "RDVI" and "MSR".<br>29.Line 294: what "NIR" and "VIS" represented?<br>30.Line 301: "EVI (enhanced vegetation index)" should be changed into "enhanced vegetation index (EVI)". Please check the similar mistake carefully in the main text.<br>31.Line 305: you should add the meaning of "RED" and "BLUE".<br>32.Line 267-312: the summarization of indexes in Table 1 is appreciated. But the "Vegetation indexes" part may not be appropriate for the "Image pre-processing" part, and this part is too redundant.<br>33.Line 320: 3D or 3-D.<br>34.Line 336-337: please add the references of the "1500-1590 nm" and "1390-1430 nm".<br>35.Line 355: Despite RGB and HSV colour space, other colour components such as ExG are also frequently used in plant detection. The authors should introduce more colour components.<br>36.Line 359-360: please add the references of the "hue can discriminate to detect chlorophyll".<br>37.Line 368: what is the meaning of "h(.)"?<br>38.Line 394: please add the references of the "Gaussian Mixture Model (GMM)". And what is the meaning of "I"?<br>39.Line 474: what are the meaning of "(892-934)" and "(281-245)"?<br>40.Line 476: 28 in SURF?<br>41.Line 487: please use the full name of "FAST" for the first time.<br>42.Line 442-517: The authors give too much detail about the features. Little was introduced about the application of these features in plant phenotyping.<br>43.Line 538-544: The authors should give some suggestion about when to select supervised/unsupervised techniques.<br>44.Line 545-547: I agree that the selection of ML algorithm require actual experimentation for optimal results. However, there are some general advices, the author should mention that and give some suggestions.<br><br>We have rewritten this part to make it more accessible. As a result, all the comments have been taken into account<br>"<br><br>As you can see, the major rewriting does take into account your ideas and suggestions.<br><br>1.The authors should summarize the imaging techniques in one table, which includes sensors, applications, advantages, disadvantages, and so on.<br><br>The authors could find a good example in Table 1 of the reference: "44. Fiorani F, Schurr U. Future scenarios for plant phenotyping. Annu. Rev. Plant Biol."<br><br>-We wrote a table as suggested. Please notice that the table is empty on the algorithms of machine learning and ToF. As of today there isn't a single paper published applied to phenomics.<br>The aforementioned table does describe advantages and disadvantages of sensors(Fiorani and Schurr). However, during image analysis, the advantages or disadvantages are a case by case situation. Researchers end up using the procedure giving better results for the specific image acquisition setup.<br><br>The complex combinations of background, reflected image, wavelength, sensors and the rest of elements that comprise image acquisition, makes it impossible to come with a single recipe for image analysis. The current review tries to give a number of methods for the different stages of image processing. But it will always be the researcher the one to test the different approaches to identify the combinations that give better results.<br><br>So it would be misleading to write for instance that a SIFT algorithm will have advantages over RGB2Grayscale for stereo vision (see new table 4), as it will depend

	on the image, background, type of plant, etc.<br><br>The fact that there are so many publications testing different methods and algorithms shows that image analysis is very much a trial and error procedure till a defined pipeline is setup for a specific need. We think this message has to get through.<br><br>2.In image analysis section, the structure should be re-organized and more sub-sections should be added. For example in image pre-processing section, the review should provide different strategies according to different sensors or data types (such as CT raw data, hyperspectral raw data, RGB raw data, infrared raw data, and so on).<br>We disagree with this as once an image is taken, the downstream process does not depend on the type of sensor used. We made table 4 to show what has been done so far, as requested. But once you have a matrix of pixels, the rest should be common procedures.<br><br>We have included a comprehensive table (Table 4) has been included as requested<br><br>Line 574:<br>A summary of the complete pipeline of image analysis including sensors, preprocessing, segmentation procedures, feature extractions and machine learning algorithms can be found in Table 4.<br><br><br>I believe that different imaging data need different pre-processing strategies. The authors just reviewed many pre-processing methods, but why choose this method and how to apply is lacking.<br><br>-As we said before this is incorrect, an image is a matrix of pixels. Authors test several methods and report the one that gave the best results.<br><br>The authors stated that: "We cannot conclude that a single preprocessing method will outperform other methods". Thus, the better or common used preprocessing method to handle different images is needed and should be listed in one table, which can benefit broader readers. The similar suggestions of the section "image segmentation, feature extraction" should be considered to modify this review.<br><br>We have added on table 4 a list of the most common used procedures. How to apply the different methods is a matter of taking the raw data and using different procedures and see for EACH case what gives better results. As we wrote above, recommending one over the other would be misleading. Furthermore, the current list of methods will probably increase in the future as the number of labs doing image analysis has increased dramatically in the last years.
**Additional Information:**	
**Question**	**Response**
Are you submitting this manuscript to a special series or article collection?	No
**Experimental design and statistics**<br><br><br>Full details of the experimental design and statistical methods used should be given in the Methods section, as detailed in our Minimum Standards Reporting Checklist. Information essential to interpreting the data presented should be made available in the figure legends.<br><br><br>Have you included all the information requested in your manuscript?	No

Powered by Editorial Manager® and ProduXion Manager® from Aries Systems Corporation

If not, please give reasons for any omissions below.<br><br>as follow-up to "**Experimental design and statistics**<br><br>Full details of the experimental design and statistical methods used should be given in the Methods section, as detailed in our Minimum Standards Reporting Checklist. Information essential to interpreting the data presented should be made available in the figure legends.<br><br>Have you included all the information requested in your manuscript?<br><br>"	Not aplicable
**Resources**<br><br>A description of all resources used, including antibodies, cell lines, animals and software tools, with enough information to allow them to be uniquely identified, should be included in the Methods section. Authors are strongly encouraged to cite Research Resource Identifiers (RRIDs) for antibodies, model organisms and tools, where possible.<br><br>Have you included the information requested as detailed in our Minimum Standards Reporting Checklist?	Yes
**Availability of data and materials**<br><br>All datasets and code on which the conclusions of the paper rely must be either included in your submission or deposited in publicly available repositories (where available and ethically appropriate), referencing such data using a unique identifier in the references and in the “Availability of Data and Materials” section of your manuscript.<br><br>Have you have met the above requirement as detailed in our Minimum Standards Reporting Checklist?	Yes

Manuscript
Click here to download Manuscript GIGA-D-17-00043_reviewed2 NN.docx

Gigascience Review

Plant phenomics: an overview of image acquisition technologies and image data analysis algorithms

Fernando Perez-Sanz1, Pedro J. Navarro2, Marcos Egea-Cortines2

[1]Genetics, ETSIA, Instituto de Biotecnología Vegetal, Universidad Politécnica de Cartagena, 30202 Cartagena, Spain

[2]DSIE, Universidad Politécnica de Cartagena, Campus Muralla del Mar, s/n. Cartagena 30202, Spain

email- Fernando Perez-Sanz- fernando.perez8@um.es; Pedro J. Navarro pedroj.navarro@upct.es

Correspondence Marcos Egea-Cortines marcos.egea@upct.es

Abstract

The study of phenomes or phenomics has been a central part of biology. The field of automatic phenotype acquisition technologies based on images has seen an important advance in the last years. As other high throughput technologies, it bears from a common set of problems, including data acquisition and analysis. In this review, we give an overview of the main systems developed to acquire images. We give an in-depth analysis of image processing with its major issues, and the algorithms that are being used or emerging as useful to obtain data out of images in an automatic fashion.

Keywords: algorithms; artificial vision; deep learning; hyperspectral cameras; machine learning; segmentation

Background

The development of systems to monitor large fields using Normalized Difference Vegetation Index (NDVI), started a long successful career over 25 years ago when NDVI was used in the so-called remote sensing field [1]. It was an important milestone in the advance of automatic methods for analysing plant growth and biomass [2]. Ever since, new technologies have increased our capacity to obtain data from biological systems. The ability to measure chlorophyll status from satellite images allowed plant health to be measured in large fields and predict crops and productivity in very large areas such as the Canadian prairies, Burkina Faso or the Indian Basin in Pakistan [3–6]. Thus, the field of remote sensing is an important basis where knowledge about data acquisition and analysis started. The development of phenotyping devices using local cameras for crops took off using an array of technologies including Infrared thermography to measure stomatal opening or osmotic stress [7–9]. Extraction of quantitative data from images has been developed to study root development [10–12], and has found a niche to identify germplasm resistant to abiotic stresses in plants such as cereals [13], Arabidopsis [14] and for large-scale field phenotyping [15]. There are several recent reviews addressing the different types of growing setups [16–22], and we will not cover them in the current review.

Two main aspects to consider are the type of image acquired and how to process it. There are a number of recent reviews on phenomics and high-throughput image data acquisition [15,23–26]. In contrast, the majority of the literature concerning image processing and analysis is found in books where methods are described in detail [27–31]. There are some very good reviews on aspects of data acquisition and analysis i.e. imaging techniques [32], Machine Learning (ML) for high throughput phenotyping [33] or software for image analysis [34], but a detailed review on different type of data analysis is lacking. In this review, we cover the current and emerging methods of image acquisition and processing allowing image-based phenomics (Figure 1).

Review

Image acquisition

Image acquisition is the process through which we obtain a digital representation of a scene. This representation is known as image and its elements are called pixels (picture elements). The electronic device used to capture a scene is known as imaging sensor. CCD (charge-coupled device) and CMOS (complementary metal oxide semiconductor) are the most broadly used technologies in image sensors. A light wavelength is captured by small analogic sensors, which will acquire major or minor charge depending on the amount of incident light. These signals are amplified, filtered, transported and enhanced by means of specific hardware. A suitable output interface and a lens in the same housing is all that it is needed to perform image acquisition. The elements enumerated above conform the main element of computer vision systems, the camera. Time delay and integration (TDI) is an imaging acquisition mode that can be implemented over CCD [35] or CMOS [36]. It improves the features of the image acquisition system considerably. TDI is used in applications that require the ability to operate in extreme lighting conditions, requiring both high speed and high sensitivity, for example: inline monitoring, inspection, sorting, and remote sensing (for weather o vegetation observation) [36].

The aforementioned technologies, CCD, CMOS and TDI confer unique characteristics, which define the type of data a camera can provide with a degree of robustness. There are fundamental differences in the type of performance the different sensors offer. In the last years CMOS technology, has outperformed CCDs in most visible imaging

applications. When selecting an imaging sensor (a camera), CCD technology causes less noise and produces higher quality images, mainly in scenes with bad illumination. They have a better depth of colour due to their higher dynamic range. On the other hand, the CMOS sensors are faster at processing images. Due to the hardware architecture for pixel extraction, they need less electrical power to operate, they allow a Region of Interest (ROI) to be processed on the device and are cheaper than CCDs. Furthermore, TDI mode with CCD or CMOS imaging sensors is used for high speed and low light level applications [37]. The latest technological developments in cameras show that the trend of the manufacturers such as IMEC, world-leader in nanoelectronics, is to fuse TDI technology with the CCD and CMOS characteristics in the same device [38]. TDI technology is expected to be applied to high throughput phenotyping processes in the nearby future.

The field of image acquisition is extremely developed with considerable literature but image acquisition systems can be classified into seven groups that are suitable for phenotyping.

1. Mono-RGB vision

Mono-RGB vision systems are composed of a set comprising a lens, imaging sensor, specific hardware and IO interface. Depending if they use a line or matrix of pixels, they are classified in line cameras (or scanners) and matrix cameras. Most computer vision phenotyping devices are based on mono-RGB vision systems. Examples of mono-RGB vision devices include “Smart tools for Prediction and Improvement of Crop Yield (SPICY)”, an automated phenotyping prototype of large pepper plants in the greenhouse. The system uses multiple RGB cameras to extract two types of features: features from a 3D reconstruction of the plant canopy and statistical features derived directly from RGB images [39]. A different approach has been used with two cameras inside a growth chamber to measure circadian growth features of *Petunia, Antirrhinum* and *Opuntia* [40]. Two cameras with low and high magnifications were used to carry-out phenotype studies of *Arabidopsis thaliana* seeds. The system is mounted on a three-axis gantry and the rotation of the samples allow the gravitropic bending response to be determined in the roots and its posterior quantification [41]. Recently a high-throughput RGB system has been developed to identify Quantitative Trait Loci (QTL) involved in

yield in large recombinant inbred lines in maize [42], demonstrating the increasing impact of this approach in phenomics.

These devices have excellent spatial and temporal resolution, i.e. they can produce a very large number of images in very short periods and at a very low cost. They are portable and there are many software tools to perform image processing (Table 1). Systems based on mono-RGB vision allow a quantification of the plant canopy [43], as well as sufficient computation of vegetation indices, for most purposes. The main disadvantages are caused by the overlap of plant organs during growth and nutation phases and the relative position of the organs with respect to the device that makes the precise quantification difficult. In addition, these devices are affected by variations in illumination when used outdoors. The trend in outdoor plant phenotyping is to combine mono-RGB systems with other systems such as Light Detection and Ranging LIDAR devices (see below), thermal imaging or adding new bands or filters to the camera that allow the segmenting of specific regions of the spectrum [44,45].

2. Stereo vision

Stereo vision systems try to correct a drawback of mono-RGB vision systems for distance measurement. The architecture of stereo vision systems emulates the behaviour of human vision using two mono vision systems. Basically, and after locating a point in two mono vision systems, it is possible to compute the distance from the point to the system. Images produced are known as depth maps [46]. A stereo vision system has been used by Biskup and colleagues [47] to obtain structural features of plant canopies. The 3D reconstruction has been successfully employed to obtain 3-D models of plants, thus demonstrating the power of this approach [48]. Simple depth reconstructions helped to define stems, leaves and grapes showing the potential of this technology [49]. A RGB camera mounted on a mobile robot is used as an automated 3D phenotyping of vineyards under field conditions. Sequentially, the system captures a set of images, which are used to reconstruct a textured 3D point cloud of the whole grapevine row [50]. A stereo vision has been developed to perform high throughput analysis of rapeseed leaf traits. The system uses two identical RGB cameras to obtain stereo images for canopy and 3-D reconstruction [51]. Developing a 3D-mesh segmentation has allowed cotton growth to be analysed [52], showing the further possibilities of 3D imaging.

The main advantage of 3-D systems is their simplicity, two cameras are enough to obtain depth maps. The stereo vision has evolved in multi-view stereo (MSV) and has found a place in plant phenotyping [53]. Furthermore, the MSV is a low cost 3D image acquisition system compared with other technologies such as LIDAR or tomography imaging [54]. Stereo vision systems have important weaknesses. They are affected by changes of the scene illumination, they need a high performance computational system to carry out stereo matching algorithms, and they have a poor depth resolution [55]. These limitations are increased in outdoor environments, as image segmentation becomes more challenging.

3. Multi and hyper spectral cameras

The multispectral and hyperspectral cameras have been used in numerous fields of science and in industrial applications [56–61]. The spectral resolution is the main factor that distinguishes multispectral imagery from hyperspectral imagery [62]. Multispectral cameras are devices able to capture images from a number of discrete spectral bands. The number of bands has increased in the last decade as technology has improved. Currently, the main camera manufacturers offer multispectral cameras acquiring between three and twenty five bands, including the visible RGB channels, Near Infra-Red (NIR) or a set of custom bands, with a tendency to provide increasing number of bands [63]. The spectral bands may not be continuous, thus for one pixel we obtain a vector of information comprising the number of elements corresponding to the number of bands registered. Hyperspectral systems may reach resolutions of a few nanometers in wavelength, obtaining for each pixel a digital signature that may contain several hundreds of continuous bands within a specific range of wavelengths [64]. Traditionally, both multispectral and hyperspectral imaging have been used for remote sensing and have an increased number of applications in phenomics. A multispectral system has been developed to improve the original colour of images for fruit recognition [65]. The authors fused the original colour image with an infrared image using the nonlinear Daubechies wavelet transform (DWT). Thus, the additional information from a second image allows the original one to be improved.

The use of hyperspectral cameras is increasing in phenotyping experiments as they allow the identification of physiological responses, pathologies or pests in a non-invasive way. Using hyperspectral images, a system has been developed to identify pathogens in barley leaves using probabilistic topic models [66]. A hyperspectral microscope was used to determine spectral changes on the leaf and cellular level of barley (*Hordeum vulgare*) during resistance reactions against powdery mildew (*Blumeria graminis f.sp. hordei, isolate K1*) [67]. A detailed description of the different wavelengths and combinations used in multispectral and hyperspectral cameras can be seen in Figure 2, and their uses in Table 2. We expect to see an increase in phenomic setups using multispectral and hyperspectral cameras in the future. An emerging issue will be the data analysis as the number of pictures doubles with each additional spectrum used for analysis (see below).

4. ToF cameras

The Time of Flight cameras or ToF cameras have been one of the last imaging devices to be incorporated into automatic plant phenotyping [68]. ToF has as a general principle the measurement of the distance between the objective of the camera and each pixel. This is achieved measuring the time it takes for a signal emitted in NIR to come back, reflected by the object. This allows a precision 3D reconstruction. Stereo vision coupled with ToF images have been implemented to increase the performance of methods of image segmentation to obtain leaf areas [69]. Beyond the tedious hand work required for manual analysis sampling is done in a non-destructive way. Depth maps obtained by a ToF camera together with colour images are used to carry out the 3D modelling of leaves. The system is mounted on a robotic arm which allows image acquisition to be automated [70]. A ToF has been successfully used to identify QTL regulating shoot architectures of *Sorghum* by mean of 3D reconstruction [71].

Microsoft Kinect is a low cost image acquisition system designed for video gaming which can be used for characterization and for tracking of phenological parameters [72]. The device is composed of an infrared projector and camera that generates a grid from which the location of a nearby object in 3 dimensions can be ascertained [73]. Kinect has been used to measure plant structure and size for two species growing in California grassland [74]. The quantitative 3D measurements of the architecture of the shoot and

structure of the leaves can be performed when proper segmentation algorithms are developed suggesting some potential for ToF systems [75].

The main disadvantages of this acquisition system are the low resolution, a reduced distance range of a few meters and the high dependence on the reflecting surface for imaging. As a result, they cannot operate under strong sunlight and are more appropriate for indoor conditions. Its reduced cost and the possibility of obtaining 3D structures of entire plants, as well as of individual organs make these devices very attractive for indoor phenotyping.

5. LIDAR technology

Light Detection and Ranging (LIDAR) is a remote sensing technology developed at the beginning of the 70s to monitor the Earth's Surface [76]. LIDAR uses a laser pulse light to measure the distance between the light source and the object by calculating the time of emission and time of reflected light detection. It allows the creation of a cloud of points that reconstruct the 3D structure of an object [77,78]. LIDAR has been used in image acquisition from distances of thousands of kilometres to centimetres, demonstrating the great potential of these type of devices. Satellite-based LIDAR systems are used for the measurements of vegetation canopy height, area, volume or biomass, etc. [79–81]. Recent development using both manned and unmanned flights have allowed the estimation of biomass dynamics of a coniferous forest using Landsat satellite images together with ground and airborne LIDAR measurements [82].Terrestrial LIDAR sensors are applied to detect and discriminate maize plants and weeds from soil surface [83]. Short range LIDAR can be deployed for high-throughput phenotyping (HTP) systems for cotton plant phenotyping in the field [84] or tomato leaf area by 3-D laser reconstruction [85]. Fully automated crop monitoring is feasible using centimetre ranges from robotized or gantry systems [43]. An autonomous robotic system has allowed 3D mapping of plant structures to be performed with millimetric precision [86]. A LASER SCAN mounted on a XYZ gantry system was used to estimate the growth measures and structural information of plants through laser triangulation techniques [87]. Thus, using different devices LIDAR has an impressive range of possibilities for plant phenomics.

Some shortcomings of LIDAR devices for pant phenotyping are the absence of colour in the measurement, excessive time to compute the cloud points, low precision for massive phenotyping, scanning noises caused by wind, rain, insects, small particles in the air, and the requirement of calibration. Recent advantages suggest that the use of LIDAR technologies could overcome some of challenges for the next-generation phenotyping technologies [88]. Developments in multispectral LIDAR instruments show novel systems which are capable of measuring multiple wavelengths and of obtaining vegetation indexes (see below) [89,90] or to measure arboreal parameters [91]. The massive adoption of LASER technologies by autonomous car manufactures has fostered the development of 3D High Definition LIDAR (HDL) with real time (RT) capacities. The new 3D HDLs are capable of generating 1.3 million points per second with precisions of 2 cm and distances of up to 120 meters [92]. These new devices open the door to the RT massive phenotyping in outdoor and indoor crops.

6. Thermography and Fluorescence Imaging

Thermography is a widely-used technology in remote sensing and plant phenotyping [93–96]. Thermographic cameras are able to acquire images at wavelengths ranging from 300 to 14,000nm [97], thus allowing the conversion of the irradiated energy into temperature values, once the environmental temperature is assessed. Plants open stomata in response to environmental cues and circadian clock depending on the type of photosynthetic metabolism they have [98,99]. The evapotranspiration can be assessed with thermography [100], and quantification can be made at different scales such as a leaf, a tree, a field or a complete region. Water stress and irrigation management are two fields of application of thermography imaging [101–104]. Thermography imaging can detect local changes of temperature produced due to pathogen infection or defence mechanisms [105]. *Oerke et al.* used a digital infrared thermography to correlate the maximum temperature difference (MTD) of apple leaves with all stages of scab development [106].

Fluorescence imaging has been used in a large number of experimental setups as UV light in the range of 340-360 nm is reflected by different plant components as discrete wavelengths [32]. The corresponding wavelengths emitted are cinnamic acids in the range of green-blue (440-520 nm). Early experiments using reflected fluorescence

allowed the identification of phenylpropanoid synthesis mutants in Arabidopsis [107]. Chlorophyll fluorescence emits in red and far-red (690-740 nm). It is an important parameter that has been studied as a proxy for different biological processes such as circadian clock or plant health [8,108,109]. A system based on a UV light lamp and a conventional camera provided of a UV-filter to avoid RGB and IR images has been used to identify changes in UV absorbance related to pollination [110]. Multicolour fluorescence detection uses the combination of chlorophyll and secondary metabolites emitted fluorescence to determine plant health in leaf tissues [111].

Thermography imaging results in an estimable tool for monitoring of genotypes and detection of plant diseases [112] where all the specimens are located under strict control conditions: temperature, wind velocity, irradiance, leaf angle or canopy leaf structures are potential issues for quality image acquisition. The next generation of thermography imaging for phenotyping will have to resolve drawbacks related to temporal variations of environment conditions, aspects relating to angles of view, distance, sensitivity and reproducibility of the measurements [104]. Both thermographic and fluorescent images capture a single component and images are in principle easy to analyse as segmentation based on thresholds can be applied to the acquired images. Combining thermographic and fluorescent imaging requires sophisticated data analysis methods based on neural networks to obtain quality data but are an emerging solution [111].

7. Tomography imaging

Magnetic Resonance Imaging (MRI) is a non-invasive imaging technique which uses Radio Frequency (RF) magnetic fields to construct tomographic images [113]. Commonly MRI has been used to investigate the anatomy structure of the body (especially the brain) in both health and disease [114]. In plant phenomics, MRI is used to visualize internal structures and metabolites. This method poses a great potential to monitor physiological processes occurring *in vivo* [115]. MRI has allowed the development of root systems over time in bean to be mapped [116], moisture distribution to be visualized during development in rice [117] and the water presence to be analysed during maturity process of barley grains [118].

Positron Emission Tomography (PET) is a nuclear medicine imaging modality that allows the assessment of biochemical processes *in vivo*, to diagnose and stage diseases

and monitor their treatment [119]. *Karve et al*. [120] presented a study about C-allocation (Carbon allocation from CO2 through photosysthesis) in large grasses such as *Sorghum bicolor*. The study concluded that the commercial PET scanners can be used reliably, not only to measure C-allocation in plants but also to study dynamics in photoassimilate transport.

X-ray Computed Tomography (X-ray CT) employs X-rays to produce tomographic images of specific areas of the scanned object. The process of attenuation of rays together with a rotation and axial movement over objects produces 3D images [32]. A high throughput phenotyping system based on X-ray CT is ten times more efficient than human operators, being capable of detecting a single tiller mutant among thousands of rice plants [121]. The remarkable penetration of X-rays, has made this technology a great ally of phenotyping carried out below-ground. The study of root systems and their quantification has been a field of habitual application of X-ray CT [122–126]. New developments address the reduction of penetrability and the increase of the image resolution of X-ray CT in plant tissue using phosphotungstate as a contrasting agent, due to its capacity of increasing the contrast and penetrability of thick samples [127].

MRI, PET and X-ray imaging techniques are available for screening 3-D objects. MRI and PET are two non-destructive and non-invasive scanning technologies that have been applied in plant sciences to acquire 3-D structural information [128]. MRI and PET data acquisition is time consuming, and software tools need to be further developed to analyse data and obtain physiologically interpretable results [97]. High-Resolution X-ray computed tomography (HRXCT) promises to be the broadest non-destructive imaging method used in plant sciences. HRXCT will provide 3-D data at a resolution suited for detailed analysis of morphological traits of *in vivo* plant samples and at a cellular resolution for *ex vivo* samples [128]. From of a point of view of the devices the trend will be to increase the resolution of images, the size of the fields of view, and increase its portability [129].

Image analysis

Extracting information from images is performed through the process of segmentation. The aim of a segmentation procedure is to extract the components of an image that are of interest i.e. object or region of interest from the rest of the image i.e. background of the image or irrelevant components. Thus, we end up with a partitioned image with significant regions. The significant regions may be defined as foreground versus background, or by selecting a number of individual components from an image. The construction of the selected regions is based on the image characteristics such as colour (colour spaces), spectral radiance (vegetation indexes), edge detection, neighbour similarity [130] or combinations that are integrated via a machine learning process [131]. In some cases, pre-processing is required in order to obtain a meaningful segmentation.

1. Image pre-processing

Image preprocessing is an important aspect of image analysis. The aim of image preprocessing is to improve contrast and eliminate noise in order to enhance the objects of interest in a given image [132]. This process can be extremely helpful to enhance the feature extraction quality and the downstream image analysis [133]. Preprocessing can include simple operations such as image cropping, contrast improvement or others significantly more complex such as dimensionality reduction via Principal Component Analysis or Clustering [33]. One preprocessing pipeline has been proposed for plant phenotyping based on converting the image to grayscale, application of a median filter, binarization and edge detection [134]. A similar preprocessing has been developed to identify plant species under varying illumination conditions [135]. It comprises conversion to grayscale, image binarization, smoothing and application of a filter to detect edges. In a comparative study to analyze leaf diseases, histogram equalization was found to be the best way to obtain preprocessing of color images converted to grayscale [136]. However RGB images have been found to perform better than grayscale conversions when identifying leaf pathogens [137].

We cannot conclude that a single preprocessing method will outperform other methods. The quality and type of image are fundamental to select a type of preprocessing procedure. Nevertheless, preprocessing is a basic step that can improve image analysis,

and sometimes make it possible. It should be described in the materials and methods ofimage procedures to make data comply the new standards -Findability, Accessibility, Interoperability, and Reusability (FAIR) [138]

2. Image segmentation

As we mentioned above, image segmentation is the core of image processing for artificial vision-based plant phenotyping. Segmentation allows the isolation and identification of objects of interest from an image, and it aims to discriminate background or irrelevant objects [139]. The objects of interest are defined by the internal similarity of pixels in parameters such as texture, colour, statistic [133], etc. (See a list of Open software libraries for image segmentation in Table 1).

One of the simplest algorithms used is threshold segmentation, based on creating groups of pixels on a grayscale according to the level of intensity, thus separating the background from targets. Such an approach has been used with Android OS (ApLeaf) in order to identify plant leaves [140].

The Otsu's method [141] is a segmentation algorithm that searches for a threshold that minimizes the weighted within class variance [132]. This method has been used for background subtraction in a system that records and performs automatic plant recognition [142], and can give high contrast segmented images in an automatic fashion [143]. Under certain circumstances, it can underestimate the signal causing under segmentation, and is significantly slower than other thresholding methods [132].

The Watershed [144] transformation is a popular algorithm for segmentation. It treats an image as a topological surface that is flooded, and seed regions are included, usually by the user. This generates an image with gradients of magnitudes, where crests appear in places where borders are apparent (strong edges), and causes segmentation to stop at those points [130]. It has been used to identify growth rate [145], recognition of partially occluded leaves [56], individual tree crown delineation [146] or leaf segmentation [147].

Grabcut [148] is a segmentation algorithm based on graph cut [149]. It is created on graph theory to tackle the problem of separating an object or foreground from the background. The user should mark a rectangle (bounding box) surrounding the object of interest thus defining the outrebound of the box as background [150]. This algorithm has been tested to extract trees from a figure but it has been successful only with very simple backgrounds [151]. More recently Grabcut has been deployed as a segmentation algorithm in a pipeline for plant recognition with multimodal information i.e. leaf contour, flower contour etc [152]. Grabcut loses precision or even fails when pictures have complex backgrounds but is highly precise with simple backgrounds [151,153].

Snakes are a special type of active contour [154], and are used as methods to fit lines (splines) either to open or close edges and lines in an image. These methods have been used for face recognition, iris segmentation and medical image analysis. Within the field of plant phenotyping, there are procedures where active contours are used inside a protocol constructing a vector of features with data of colour intensity, local texture and a previous knowledge of the plant incorporated via Gaussian Mixture Models, previously segmented [155] . These steps give an initial rough segmentation upon which, active contours can operate with a much higher precision.

Active contours have used for plant recognition via images of flowers [156], based on a combination of the algorithm proposed by Yonggang and Karl [157] and the model of active contours without edges [158]. Whilst the work proposed by Minervini et al [155] appears to give significantly better results compared to those of Suta et al [156], the usage of images with a natural background maybe related to the apparent differences in segmentation. Thus, a current problem concerning the comparison of algorithms and procedures lies on the different backgrounds used for image acquisition.

3. Features extraction

Features extraction constitutes one of the pillars of the identification and classification of objects based on computer vision. Beyond the raw image, a feature is information which is used to resolve a specific computer vision problem. The features extracted from an image are disposed in the so-called “feature vectors”. The construction of feature vectors uses a wide set of methods to identify the objects in an image. The main

features are edges, intensity of image pixels [39], geometries [159], textures [155,160], image transformations e.g. Fourier [161], or Wavelet [65,162] or combinations of pixels of different colour spaces [131]. The end goal of feature extraction is to feed up a set of classifiers and machine learning algorithms (see below).

One system proposed uses a feature vector composed of a combination of RGB and CIE L*a*b* colour spaces to segment the images captured during the day [131]. The night-time image segmentation computed a vector composed of statistical features over two decomposition levels of the wavelet transform using IR images.
Iyer-Pascuzzi et al. presented an imaging and analysis platform for automatic phenotyping to identify genes underlying root system architecture. The authors employed a set of 16 statistical, geometrics and shape features obtained from 2,297 images from 118 individuals such as median and maximum number of roots, the total root length, perimeter, depth, among others [163].

There are a number of algorithms to identify invariant features detectors and descriptors. This type of image analysis ensures the detection of points of interest in a scale and rotation independent manner. This is crucial for camera calibration and for matching to produce a set of corresponding image points in 3D image reconstruction. Furthermore, it allows the identification of points of interest even when they change scale and/or position or situations of uncontrolled illumination, a common issue when phenotyping plants. The Scale Invariant Features Transforms (SIFT) [164], Speeded-Up Robust Features (SURF) [165] and the Histograms of Oriented Gradients (HoG) [166] are algorithms used to extract characteristics in computer vision and they have been extended to plant phenotyping. Wei et al. [167] presented an image-based method that automatically detects the flowering of paddy rice. The method uses a scale-invariant feature transform descriptor, bag of visual words, and a machine learning method. The SIFT algorithm has been used to combine stereo and ToF images with automatic plant phenotyping. It can create dense depth maps to identify pepper leaf in glasshouses [69]. SIFT and SURF algorithms have been tested for detecting local invariant features for obtaining a 3D plant model from a multi-view stereo images [168]. A HoG framework allows the extraction of a reliable quantity of phenotypic data of grapevine berry using a feature vector composed of colour information [169].

So far, feature extraction is an arduous and difficult task requiring the testing of hundreds of feature extraction algorithms and a greater number of combinations between them. This task demands expert skills in different subjects. The success in the identification does not depend on the robustness of the classification methods, but on the robustness of the data.

4. Machine Learning in plant image analysis

The amount of data generated in current and future phenomic setups with high throughput imaging technologies has brought the use of Machine Learning (ML) statistical approaches. Machine Learning is applied in many fields of research [170–172]. As phenotyping can generate Terabytes of information, ML tools provide a good framework for data analysis. A list of ML libraries can be found in Table 3. A major advantage of ML is the possibility to explore large datasets to identify patterns, using combinations of factors instead of performing independent analysis
[33].

Among the ML algorithms a predictive model of regression has been used to phenotype Arabidopsis leaves, based on geometric features as training dataset [159]. Three different algorithms were tested, k Nearest Neighbour (kNN), Support Vector Machine (SVM) and Naïve Bayes to segment Antirrhinum majus leaves. Colour images have as a characteristic vector intensity in the RBG and CIE L*a*b*, while the NIR vector is obtained with the wavelet transform. The best results were obtained with kNN for colour images and SVM for NIR. This shows that segmentation has several components as mentioned before including the wavelength of image acquisition [131].

As the specific wavelength used for image acquisition plays a key role in the type of data obtained, hyperspectral cameras are becoming important tools, however, hyper images can be in the order of Gbites of size, making ML a necessity. Examples of coupling hyperspectral and thermal imaging with ML have allowed the early detection of stress caused by *Alternaria* in Brassica [173]. The best image classification was obtained doing a second derivative transformation of the hyperspectral images together with a back propagation of neural networks allowing the identification of fungi on leaves days after infection [173].

A current concept derived from ML is Deep Learning (DL) comprising a set of algorithms aimed to model with a high level of abstraction. This allows the development of complex concepts starting from simpler ones, thus getting closer to the idea of Artificial Intelligence (AI) (www.deeplearningbook.org). Convolutional Neural Networks (CNN), are an example of DL derived of Artificial Neural Networks (ANN). These multi-layered networks are formed by a layer of neurons that work in a convolutional way reducing the sampling process and end with a layer of perception neurons for final classification [174]. Recently DL has been implemented using a CNN to automatically classify and identify different plant parts [175], thus obtaining both classification and localization that significantly improve the current methods. A CNN has been used to detect plant pathogen attacks [176]. Although the training period is computationally heavy, requiring several hours of CPU clusters, classification was performed in less than one second [176]. Nevertheless, DL is a step forward in ML and has great potential to allow the management and analysis of the data produced in phenomic experiments.

Although direct testing maybe the best way to determine the superior algorithm in each case, there is a number of examples that may guide initial approaches [33,177,178]. As a general rule discriminating methods such as SVM, ANN, K-NN, give better results in large datasets that are labelled [33]. Generative methods such as Naive Bayes, Gaussian Mixture Models, Hide Markov Models, give better results with smaller datasets, both labelled and unlabelled. The use of unsupervised algorithms i.e. k-means may help identify unexpected characteristics on a dataset. As mentioned above, preprocessing plays a fundamental role in increasing the ML output. A summary of the complete pipeline of image analysis including sensors, preprocessing, segmentation procedures, feature extractions and machine learning algorithms can be found in Table 4.

Conclusions and future prospects

The implementation of phenomic technologies is a welcome change towards reproducibility and unbiased data acquisition in basic and applied research. A successful approach requires integrating sensors, with wavelength and image acquisitions that will allow the proper identification of the items under analysis. A lot of work has been made

in indoor-setups where reasonable conditions can be created to obtain high quality images, amenable to further processing. The difficulty in outdoor setups increases as a result of limitations in the actual image acquisition devices and the uncontrolled conditions that directly affect image quality. The new technologies such as the high definition LIDAR or the multi-hyperspectral cameras have a great potential to improve in the near future, specially in outdoor environments.

The pre-processing and segmentation data are two aspects of data treatment and acquisition that require careful design in order to avoid distortions and reproducibility [138]. As images are machine-produced data, but image types and processing procedures may be very different, the standardization of image capture, preprocessing and segmentation may play an important role. Furthermore, a single procedure for image analysis cannot be considered as a better choice and it is the researcher that needs to assess the different algorithms to come with an optimized procedure for their specific setup. It is a matter of time that databases with raw image will become part of the standard in phenomics using images very much like NCBI or Uniprot play a key role in genomic and proteomic projects. With the decrease in price of hyperspectral devices, new experiments may be performed that produce even larger data sets, and these data sets will have to go through Artificial Intelligence-based data analysis in order to give the researchers results interpretable by humans. We guess that like in other omic approaches, there will be a confluence to standard procedures that are not currently common ground, making the current literature look intimidatingly diverse. Nevertheless, most of the basic processes described here are shared by the different experimental setups and data analysis pipes.

Abbreviations

AI: Artificial intelligence

ANN: Artificial neural networks

CAI: Cellulose Absorption Index

CAR: Chlorophyll absorption ratio

CCD: Charge coupled device

Cig: Coloration green

Cir: Coloration Index red

CMOS: Complementary metal oxide semiconductor
CNN: Convolutional neural networks
CPU: Central processing unit
DL: Deep learning
DLAI: Difference Leaf Area Index
DSWI: Disease water stress index
DWT: Daubechies wavelet transform
EVI: Enhanced vegetation index
FAIR: Findability, Accessibility, Interoperability, and Reusability
GI: Greenness Index
GMM: Gaussian mixture model
GNDVI: Green normalized difference vegetation index
HOG: Histograms of oriented gradients
KNN: K nearest neighbour
LAI: Leaf area index
LCA: Lignin-Cellulose Absorption Index
LIDAR: Light detection and ranging
LWVI-1: Normalized Difference Leaf water VI 1
MCARI: Modified Chlorophyll Absorption Ratio Index
MCFI: Multicolour fluorescence imaging
ML: Machine learning
NDVI: Normalized Difference Vegetation index
NIR: Near infrared
NLI: Nonlinear vegetation index
NTDI: Normalized Tillage Difference Index
OSAVI: Optimized Soil Adjusted Vegetation Index
PCA: Principal component analysis
PWI: Plant Water Index
QTL: Quantitative trait locus
RGB: Red, green, blue
ROI: Region of interest
SIFT: Scale invariant features transforms
SURF: Speeded-up robust features
SVM: Support vector machine

TDI: Time delay and integration

ToF: Time of flight

Competing interests

The authors declare they have no competing interests

Funding

This work was funded by grants FEDER BFU-2013-45148-R, Fundación Séneca 19398/PI/14 to MEC and FEDER ViSelTR (TIN2012-39279) to PJN

Availability of supporting data and material

Not applicable

Authors contributions

FPS, MEC and PJN defined the scope of the manuscript, FPS, MEC and PJN wrote and corrected the manuscript, MEC and PJN wrote the grant applications.

Acknowledgments

We would like to thank Leanne Rebecca Miller for the edition of the manuscript and Victoria Ruiz-Hernández and Julia Weiss for comments on the manuscript.

References

1. Tucker C. Red and photographic infrared linear combinations for monitoring vegetation. Remote Sens. Environ. [Internet]. 1979 [cited 2016 Oct 11]; Available from: http://www.sciencedirect.com/science/article/pii/0034425779900130

2. Myneni RB, Keeling CD, Tucker CJ, Asrar G, Nemani RR. Increased plant growth in the northern high latitudes from 1981 to 1991. Nature. 1997;386:698–702.

3. DeFries R, Townshend J. NDVI-derived land cover classifications at a global scale. Int. J. Remote [Internet]. 1994 [cited 2016 Oct 11]; Available from: http://www.tandfonline.com/doi/abs/10.1080/01431169408954345

4. Pettorelli N, Vik J, Mysterud A, Gaillard J. Using the satellite-derived NDVI to assess

ecological responses to environmental change. Trends Ecol. [Internet]. 2005 [cited 2016 Oct 11]; Available from: http://www.sciencedirect.com/science/article/pii/S016953470500162X

5. Mkhabela MS, Bullock P, Raj S, Wang S, Yang Y. Crop yield forecasting on the Canadian Prairies using MODIS NDVI data. Agric. For. Meteorol. 2011;151:385–93.

6. GROTEN SME. NDVI—crop monitoring and early yield assessment of Burkina Faso. Int. J. Remote Sens. [Internet]. Taylor & Francis Group ; 1993 [cited 2016 Dec 6];14:1495–515. Available from: http://www.tandfonline.com/doi/abs/10.1080/01431169308953983

7. Jones HG, Stoll M, Santos T, de Sousa C, Chaves MM, Grant OM. Use of infrared thermography for monitoring stomatal closure in the field: application to grapevine. J. Exp. Bot. [Internet]. Oxford University Press; 2002 [cited 2016 Dec 6];53:2249–60. Available from: http://www.ncbi.nlm.nih.gov/pubmed/12379792

8. Chaerle L, Van der Straeten D. Seeing is believing: imaging techniques to monitor plant health. Biochim. Biophys. Acta-Gene Struct. Expr. 2001;1519:153–66.

9. Sirault XRR, James RA, Furbank RT, Bernstein L, Hayward H, Flowers T, et al. A new screening method for osmotic component of salinity tolerance in cereals using infrared thermography. Funct. Plant Biol. [Internet]. CSIRO PUBLISHING; 2009 [cited 2016 Dec 6];36:970. Available from: http://www.publish.csiro.au/?paper=FP09182

10. Lobet G, Pagès L, Draye X. A Novel Image Analysis Toolbox Enabling Quantitative Analysis of Root System Architecture. Plant Physiol. [Internet]. 2011;157:29–39. Available from: http://www.ncbi.nlm.nih.gov/pubmed/21771915

11. Galkovskyi T, Mileyko Y, Bucksch A, Moore B, Symonova O, Price CA, et al. GiA Roots: software for the high throughput analysis of plant root system architecture. BMC Plant Biol. [Internet]. BioMed Central; 2012 [cited 2016 Sep 10];12:116. Available from: http://bmcplantbiol.biomedcentral.com/articles/10.1186/1471-2229-12-116

12. French A, Ubeda-Tomas S, Holman TJ, Bennett MJ, Pridmore T. High-Throughput Quantification of Root Growth Using a Novel Image-Analysis Tool. Plant Physiol. [Internet]. American Society of Plant Biologists; 2009 [cited 2016 Sep 20];150:1784–95. Available from: http://www.pubmedcentral.nih.gov/articlerender.fcgi?artid=2719150&tool=pmcentrez&rendertype=abstract

13. Golzarian MR, Frick RA, Rajendran K, Berger B, Roy S, Tester M, et al. Accurate inference of shoot biomass from high-throughput images of cereal plants. Plant Methods [Internet]. BioMed Central; 2011 [cited 2016 Sep 10];7:2. Available from: http://plantmethods.biomedcentral.com/articles/10.1186/1746-4811-7-2

14. Fabre J, Dauzat M, Negre V, Wuyts N, Tireau A, Gennari E, et al. PHENOPSIS DB: an Information System for Arabidopsis thaliana phenotypic data in an environmental context. BMC Plant Biol. 2011;11.

15. Araus JL, Cairns JE. Field high-throughput phenotyping: The new crop breeding frontier. Trends Plant Sci. 2014;19:52–61.

16. Furbank RT. Plant phenomics: from gene to form and function. Funct. Plant Biol. [Internet]. 2009 [cited 2016 Aug 31];36:V–Vi. Available from: http://citeseerx.ist.psu.edu/viewdoc/download?doi=10.1.1.547.5673&rep=rep1&type=pdf

17. Poorter H, Fiorani F, Pieruschka R, Putten WH Van Der, Kleyer M, Schurr U. Tansley review Pampered inside , pestered outside ? Differences and similarities between plants growing in controlled conditions and in the field. New Phytol. 2016;838–55.

18. Yang W, Duan L, Chen G, Xiong L, Liu Q. Plant phenomics and high-throughput phenotyping: Accelerating rice functional genomics using multidisciplinary technologies. Curr. Opin. Plant Biol. [Internet]. Elsevier Ltd; 2013;16:180–7. Available from: http://dx.doi.org/10.1016/j.pbi.2013.03.005

19. White J, Andrade-Sanchez P, Gore M. Field-based phenomics for plant genetics research. F. Crop. [Internet]. 2012 [cited 2016 Aug 31]; Available from: http://www.sciencedirect.com/science/article/pii/S037842901200130X

20. Fahlgren N, Gehan MA, Baxter I. Lights, camera, action: High-throughput plant phenotyping is ready for a close-up. Curr. Opin. Plant Biol. 2015. p. 93–9.

21. Furbank RT, Tester M. Phenomics - technologies to relieve the phenotyping bottleneck. Trends Plant Sci. 2011;16:635–44.

22. Granier C, Vile D. Phenotyping and beyond: Modelling the relationships between traits. Curr. Opin. Plant Biol. [Internet]. Elsevier Ltd; 2014;18:96–102. Available from: http://dx.doi.org/10.1016/j.pbi.2014.02.009

23. White J, Andrade-Sanchez P, Gore M. Field-based phenomics for plant genetics research. F. Crop. 2012;

24. Furbank RT, Tester M. Phenomics - technologies to relieve the phenotyping bottleneck. Trends Plant Sci. 2011;16:635–44.

25. Simko I, Jimenez-Berni JA, Sirault XRR. Phenomic approaches and tools for phytopathologists. Phytopathology [Internet]. 2016;PHYTO-02-16-0082-RVW. Available from: http://apsjournals.apsnet.org/doi/10.1094/PHYTO-02-16-0082-RVW

26. da Silva Marques J. Monitoring Photosynthesis by In Vivo Chlorophyll Fluorescence : Application to High-Throughput Plant Phenotyping. Appl. Photosynth. - New Prog. 2016;Intech:3–22.

27. Gonzalez RC, Woods RE. Digital image processing. Prentice Hall Press ; 2002.

28. Russ J, Woods R. The image processing handbook. 1995 [cited 2017 Apr 25]; Available from: http://journals.lww.com/jcat/Citation/1995/11000/The_Image_Processing_Handbook,_2nd_Ed.26.aspx

29. Jain A. Fundamentals of digital image processing. 1989 [cited 2017 Apr 25]; Available from: http://dl.acm.org/citation.cfm?id=59921

30. Sonka M, Hlavac V, Boyle R. Image processing, analysis, and machine vision [Internet]. 4th ed. CL Engineering; 2014 [cited 2017 Apr 24]. Available from: https://books.google.es/books?hl=en&lr=&id=QePKAgAAQBAJ&oi=fnd&pg=PR11&dq=image+analysis+a+review&ots=95qB21F9B-&sig=kSGTMS9GfxkddVJUHnxnBzU2VL8

31. Soille P. Morphological image analysis: principles and applications [Internet]. Springer; 2013 [cited 2017 Apr 24]. Available from: https://books.google.es/books?hl=en&lr=&id=ZFzxCAAAQBAJ&oi=fnd&pg=PA1&dq=image+analysis+a+review&ots=-oc-0SEZ6g&sig=wLoRbdNSusr-5UtgD_RvtMHVqjQ

32. Li L, Zhang Q, Huang D. A Review of Imaging Techniques for Plant Phenotyping. Sensors. 2014;14:20078–111.

33. Singh A, Ganapathysubramanian B, Singh AK, Sarkar S. Machine Learning for High-Throughput Stress Phenotyping in Plants. Trends Plant Sci. 2016. p. 110–24.

34. Fiorani F, Schurr U. Future Scenarios for Plant Phenotyping. Annu. Rev. Plant Biol [Internet]. 2013 [cited 2016 Sep 21];64:267–91. Available from: http://www.ncbi.nlm.nih.gov/pubmed/23451789

35. Lepage G, Bogaerts J, Meynants G. Time-delay-integration architectures in CMOS image sensors. IEEE Trans. Electron Devices [Internet]. 2009 [cited 2017 Apr

26];56:2524–33. Available from: http://s3.amazonaws.com/academia.edu.documents/34420865/05272462.pdf?AWSAccessKeyId=AKIAIWOWYYGZ2Y53UL3A&Expires=1493227777&Signature=%2BCCwsvCdYXhWhL83LxFrED1f9pY%3D&response-content-disposition=inline%3B filename%3D05272462.pdf

36. Yu C, Nie K, Xu J, Gao J. A Low Power Digital Accumulation Technique for Digital-Domain CMOS TDI Image Sensor. Sensors [Internet]. Multidisciplinary Digital Publishing Institute; 2016 [cited 2017 Apr 24];16:1572. Available from: http://www.mdpi.com/1424-8220/16/10/1572

37. Teledyne Dalsa. No Title. https://www.teledynedalsa.com/corp/.

38. IMEC. Imec launches TDI, multispectral and hyperspectral sensors [Internet]. [cited 2017 Apr 24]. Available from: http://optics.org/news/8/2/8

39. Van Der Heijden G, Song Y, Horgan G, Polder G, Dieleman A, Bink M, et al. SPICY: Towards automated phenotyping of large pepper plants in the greenhouse. Funct. Plant Biol. CSIRO PUBLISHING; 2012;39:870–7.

40. Navarro PJ, Fernández C, Weiss J, Egea-Cortines M. Development of a configurable growth chamber with a computer vision system to study circadian rhythm in plants. Sensors (Basel). [Internet]. 2012 [cited 2016 Sep 20];12:15356–75. Available from: http://www.ncbi.nlm.nih.gov/pubmed/23202214

41. Subramanian R, Spalding EP, Ferrier NJ. A high throughput robot system for machine vision based plant phenotype studies. Mach. Vis. Appl. [Internet]. 2013;24:619–36. Available from: http://link.springer.com/10.1007/s00138-012-0434-4

42. Zhang X, Huang C, Wu D, Qiao F, Li W, Duan L, et al. High-throughput phenotyping and QTL mapping reveals the genetic architecture of maize plant growth. Plant Physiol. [Internet]. 2017;pp.01516.2016. Available from: http://www.plantphysiol.org/lookup/doi/10.1104/pp.16.01516

43. Virlet N, Sabermanesh K, Sadeghi-Tehran P, Hawkesford MJ. Field Scanalyzer: An automated robotic field phenotyping platform for detailed crop monitoring. Funct. Plant Biol. [Internet]. 2017 [cited 2017 May 11];44:143. Available from: http://www.publish.csiro.au/?paper=FP16163

44. Deery D, Jimenez-Berni J, Jones H, Sirault X, Furbank R. Proximal Remote Sensing Buggies and Potential Applications for Field-Based Phenotyping. Agronomy [Internet].

Multidisciplinary Digital Publishing Institute; 2014 [cited 2017 May 23];4:349–79. Available from: http://www.mdpi.com/2073-4395/4/3/349/

45. Comar A, Burger P, de Solan B, Baret F, Daumard F, Hanocq J-F, et al. A semi-automatic system for high throughput phenotyping wheat cultivars in-field conditions: description and first results. Funct. Plant Biol. CSIRO PUBLISHING; 2012;39:914.

46. Brown MZ, Burschka D, Hager GD. Advances in computational stereo. IEEE Trans. Pattern Anal. Mach. Intell. [Internet]. 2003 [cited 2017 Apr 26];25:993–1008. Available from: http://ieeexplore.ieee.org/document/1217603/

47. Biskup B, Scharr H, Schurr U, Rascher U. A stereo imaging system for measuring structural parameters of plant canopies. Plant, Cell Environ. [Internet]. Blackwell Publishing Ltd; 2007 [cited 2016 Sep 20];30:1299–308. Available from: http://doi.wiley.com/10.1111/j.1365-3040.2007.01702.x

48. Nguyen TT, Slaughter DC, Max N, Maloof JN, Sinha N. Structured light-based 3D reconstruction system for plants. Sensors (Switzerland) [Internet]. Multidisciplinary Digital Publishing Institute; 2015 [cited 2016 Sep 21];15:18587–612. Available from: http://www.mdpi.com/1424-8220/15/8/18587/

49. Klodt M, Herzog K, Töpfer R, Cremers D, Töpfer R, Hausmann L, et al. Field phenotyping of grapevine growth using dense stereo reconstruction. BMC Bioinformatics [Internet]. BioMed Central; 2015 [cited 2016 Oct 7];16:143. Available from: http://www.biomedcentral.com/1471-2105/16/143

50. Rose J, Kicherer A, Wieland M, Klingbeil L, Töpfer R, Kuhlmann H. Towards Automated Large-Scale 3D Phenotyping of Vineyards under Field Conditions. Sensors [Internet]. Multidisciplinary Digital Publishing Institute; 2016 [cited 2017 May 11];16:2136. Available from: http://www.mdpi.com/1424-8220/16/12/2136

51. Xiong X, Yu L, Yang W, Liu M, Jiang N, Wu D, et al. A high-throughput stereo-imaging system for quantifying rape leaf traits during the seedling stage. Plant Methods [Internet]. BioMed Central; 2017 [cited 2017 Feb 8];13:7. Available from: http://plantmethods.biomedcentral.com/articles/10.1186/s13007-017-0157-7

52. Paproki A, Sirault XRR, Berry S, Furbank RT, Fripp J. A novel mesh processing based technique for 3D plant analysis. BMC Plant Biol. [Internet]. 2012;12:63. Available from: http://www.biomedcentral.com/1471-2229/12/63

53. Nguyen TT, Slaughter DC, Maloof JN, Sinha N. Plant phenotyping using multi-view

stereo vision with structured lights. In: Valasek J, Thomasson JA, editors. International Society for Optics and Photonics; 2016 [cited 2017 May 19]. p. 986608. Available from: http://proceedings.spiedigitallibrary.org/proceeding.aspx?doi=10.1117/12.2229513

54. Rose JC, Paulus S, Kuhlmann H. Accuracy analysis of a multi-view stereo approach for phenotyping of tomato plants at the organ level. Sensors (Basel). [Internet]. Multidisciplinary Digital Publishing Institute (MDPI); 2015 [cited 2017 May 19];15:9651–65. Available from: http://www.ncbi.nlm.nih.gov/pubmed/25919368

55. Schwartz S. An overview of 3D plant phenotyping methods [Internet]. Phenospex. Smart Plant Analyis. 2015 [cited 2017 Jun 19]. Available from: https://phenospex.com/blog/an-overview-of-3d-plant-phenotyping-methods/#ref

56. Lee K-S, Cohen WB, Kennedy RE, Maiersperger TK, Gower ST. Hyperspectral versus multispectral data for estimating leaf area index in four different biomes. Remote Sens. Environ. [Internet]. 2004 [cited 2017 Apr 27];91:508–20. Available from: http://www.sciencedirect.com/science/article/pii/S0034425704001282

57. Dozier J, Painter TH. MULTISPECTRAL AND HYPERSPECTRAL REMOTE SENSING OF ALPINE SNOW PROPERTIES. Annu. Rev. Earth Planet. Sci. [Internet]. Annual Reviews; 2004 [cited 2017 Apr 27];32:465–94. Available from: http://www.annualreviews.org/doi/10.1146/annurev.earth.32.101802.120404

58. Adam E, Mutanga O, Rugege D. Multispectral and hyperspectral remote sensing for identification and mapping of wetland vegetation: a review. Wetl. Ecol. Manag. [Internet]. Springer Netherlands; 2010 [cited 2017 Apr 27];18:281–96. Available from: http://link.springer.com/10.1007/s11273-009-9169-z

59. Qin J, Chao K, Kim MS, Lu R, Burks TF. Hyperspectral and multispectral imaging for evaluating food safety and quality. J. Food Eng. [Internet]. 2013 [cited 2017 Apr 27];118:157–71. Available from: http://www.sciencedirect.com/science/article/pii/S0260877413001659

60. van der Meer FD, van der Werff HMA, van Ruitenbeek FJA, Hecker CA, Bakker WH, Noomen MF, et al. Multi- and hyperspectral geologic remote sensing: A review. Int. J. Appl. Earth Obs. Geoinf. [Internet]. 2012 [cited 2017 Apr 27];14:112–28. Available from: http://www.sciencedirect.com/science/article/pii/S0303243411001103

61. P. M. Mehl PM, K. Chao K, M. Kim M, Y. R. Chen YR. DETECTION OF DEFECTS ON SELECTED APPLE CULTIVARS USING HYPERSPECTRAL AND MULTISPECTRAL IMAGE

ANALYSIS. Appl. Eng. Agric. [Internet]. American Society of Agricultural and Biological Engineers; 2002 [cited 2017 Apr 27];18:219. Available from: http://elibrary.asabe.org/abstract.asp??JID=3&AID=7790&CID=aeaj2002&v=18&i=2&T=1

62. Ferrato L-J. COMPARING HYPERSPECTRAL AND MULTISPECTRAL IMAGERY FOR LAND CLASSIFICATION OF THE LOWER DON RIVER, TORONTO. [cited 2017 Apr 27]; Available from: http://www.geography.ryerson.ca/wayne/MSA/LisaJenFerratoMRP2012.pdf

63. Cubert. S 137 - ButterflEYE NIR - Cubert-GmbH [Internet]. [cited 2017 Jun 4]. Available from: http://cubert-gmbh.com/product/s-137-butterfleye-nir/

64. Kise M, Park B, Heitschmidt GW, Lawrence KC, Windham WR. Multispectral imaging system with interchangeable filter design. Comput. Electron. Agric. 2010;72:61–8.

65. Li P, Lee S-H, Hsu H-Y, Park J-S. Nonlinear Fusion of Multispectral Citrus Fruit Image Data with Information Contents. Sensors [Internet]. Multidisciplinary Digital Publishing Institute; 2017 [cited 2017 Jan 23];17:142. Available from: http://www.mdpi.com/1424-8220/17/1/142

66. Wahabzada M, Mahlein A-K, Bauckhage C, Steiner U, Oerke E-C, Kersting K. Plant Phenotyping using Probabilistic Topic Models: Uncovering the Hyperspectral Language of Plants. Sci. Rep. [Internet]. Nature Publishing Group; 2016 [cited 2017 Jan 24];6:22482. Available from: http://www.ncbi.nlm.nih.gov/pubmed/26957018

67. Kuska M, Wahabzada M, Leucker M, Dehne H-W, Kersting K, Oerke E-C, et al. Hyperspectral phenotyping on the microscopic scale: towards automated characterization of plant-pathogen interactions. Plant Methods [Internet]. 2015 [cited 2017 May 11];11:28. Available from: http://www.plantmethods.com/content/11/1/28

68. Klose R, Penlington J. Usability study of 3D time-of-flight cameras for automatic plant phenotyping. Bornimer [Internet]. 2009 [cited 2017 May 2]; Available from: https://www.hs-osnabrueck.de/fileadmin/HSOS/Homepages/COALA/Veroeffentlichungen/2009-CBA-3DToF.pdf

69. Song Y, Glasbey CA, van der Heijden GWAM, Polder G, Dieleman JA. Combining Stereo and Time-of-Flight Images with Application to Automatic Plant Phenotyping.

Springer Berlin Heidelberg; 2011. p. 467–78.

70. Alenyà G, Dellen B, Torras C. 3D modelling of leaves from color and ToF data for robotized plant measuring. Robot. Autom. (ICRA), [Internet]. 2011 [cited 2017 May 2]; Available from: http://ieeexplore.ieee.org/abstract/document/5980092/

71. McCormick RF, Truong SK, Mullet JE. 3D Sorghum Reconstructions from Depth Images Identify QTL Regulating Shoot Architecture. Plant Physiol. [Internet]. American Society of Plant Biologists; 2016 [cited 2017 May 2];172:823–34. Available from: http://www.ncbi.nlm.nih.gov/pubmed/27528244

72. Paulus S, Behmann J, Mahlein A, Plümer L. Low-cost 3D systems: suitable tools for plant phenotyping. Sensors [Internet]. 2014 [cited 2017 May 2]; Available from: http://www.mdpi.com/1424-8220/14/2/3001/htm

73. Microsoft. Kinect for Windows Sensor Components and Specifications [Internet]. 2010. [cited 2017 May 7]. Available from: https://msdn.microsoft.com/en-us/library/jj131033.aspx

74. Azzari G, Goulden M, Rusu R. Rapid Characterization of Vegetation Structure with a Microsoft Kinect Sensor. Sensors [Internet]. Multidisciplinary Digital Publishing Institute; 2013 [cited 2017 May 7];13:2384–98. Available from: http://www.mdpi.com/1424-8220/13/2/2384/

75. Chéné Y, Rousseau D, Lucidarme P, Bertheloot J, Caffier V, Morel P, et al. On the use of depth camera for 3D phenotyping of entire plants. Comput. Electron. Agric. 2012;82:122–7.

76. Wang G, Weng Q. Remote sensing of natural resources. [cited 2017 May 9]. p. 532. Available from: https://books.google.es/books?id=wIDNBQAAQBAJ&pg=PA9&dq=Light+Detection+and+Ranging+(LIDAR)+1970s&hl=es&sa=X&ved=0ahUKEwi0mbSksePTAhVJDxoKHaKxC6UQ6AEIJjAA#v=onepage&q=Light Detection and Ranging (LIDAR) 1970s&f=false

77. Lin Y. LiDAR: An important tool for next-generation phenotyping technology of high potential for plant phenomics? Comput. Electron. Agric. Elsevier B.V.; 2015;119:61–73.

78. Vázquez-Arellano M, Griepentrog HW, Reiser D, Paraforos DS. 3-D Imaging Systems for Agricultural Applications-A Review. Sensors (Basel). [Internet]. Multidisciplinary Digital Publishing Institute (MDPI); 2016 [cited 2017 May 2];16. Available from: http://www.ncbi.nlm.nih.gov/pubmed/27136560

79. Chen JM, Cihlar J. Retrieving leaf area index of boreal conifer forests using Landsat TM images. Remote Sens. Environ. [Internet]. 1996 [cited 2016 Sep 26];55:155–62. Available from: http://linkinghub.elsevier.com/retrieve/pii/0034425795001956

80. Gwenzi D, Helmer E, Zhu X, Lefsky M, Marcano-Vega H. Predictions of Tropical Forest Biomass and Biomass Growth Based on Stand Height or Canopy Area Are Improved by Landsat-Scale Phenology across Puerto Rico and the U.S. Virgin Islands. Remote Sens. [Internet]. Multidisciplinary Digital Publishing Institute; 2017 [cited 2017 May 9];9:123. Available from: http://www.mdpi.com/2072-4292/9/2/123

81. Kellndorfer JM, Walker WS, LaPoint E, Kirsch K, Bishop J, Fiske G. Statistical fusion of lidar, InSAR, and optical remote sensing data for forest stand height characterization: A regional-scale method based on LVIS, SRTM, Landsat ETM+, and ancillary data sets. J. Geophys. Res. Biogeosciences [Internet]. 2010 [cited 2017 May 9];115:n/a-n/a. Available from: http://doi.wiley.com/10.1029/2009JG000997

82. Badreldin N, Sanchez-Azofeifa A. Estimating Forest Biomass Dynamics by Integrating Multi-Temporal Landsat Satellite Images with Ground and Airborne LiDAR Data in the Coal Valley Mine, Alberta, Canada. Remote Sens. [Internet]. Multidisciplinary Digital Publishing Institute; 2015 [cited 2017 May 9];7:2832–49. Available from: http://www.mdpi.com/2072-4292/7/3/2832/

83. Andújar D, Rueda-Ayala V, Moreno H, Rosell-Polo JR, Escolà A, Valero C, et al. Discriminating crop, weeds and soil surface with a terrestrial LIDAR sensor. Sensors (Switzerland) [Internet]. Multidisciplinary Digital Publishing Institute; 2013 [cited 2017 May 8];13:14662–75. Available from: http://www.mdpi.com/1424-8220/13/11/14662/

84. Sun S, Li C, Paterson A. In-Field High-Throughput Phenotyping of Cotton Plant Height Using LiDAR. Remote Sens. [Internet]. Multidisciplinary Digital Publishing Institute; 2017 [cited 2017 May 8];9:377. Available from: http://www.mdpi.com/2072-4292/9/4/377

85. Hosoi F, Nakabayashi K, Omasa K. 3-D modeling of tomato canopies using a high-resolution portable scanning lidar for extracting structural information. Sensors (Basel). [Internet]. Multidisciplinary Digital Publishing Institute (MDPI); 2011 [cited 2017 May 7];11:2166–74. Available from: http://www.ncbi.nlm.nih.gov/pubmed/22319403

86. Chaudhury A, Ward C, Talasaz A, Ivanov AG, Norman PAH, Grodzinski B, et al.

Computer Vision Based Autonomous Robotic System for 3D Plant Growth Measurement. 12th Conf. Comput. Robot Vis. 2015;290–6.

87. Kjaer KH, Ottosen C-O. 3D Laser Triangulation for Plant Phenotyping in Challenging Environments. Sensors (Basel). [Internet]. Multidisciplinary Digital Publishing Institute (MDPI); 2015 [cited 2017 Jan 18];15:13533–47. Available from: http://www.ncbi.nlm.nih.gov/pubmed/26066990

88. Lin Y. LiDAR: An important tool for next-generation phenotyping technology of high potential for plant phenomics? Comput. Electron. Agric. [Internet]. 2015 [cited 2017 May 8];119:61–73. Available from: http://www.sciencedirect.com/science/article/pii/S0168169915003245

89. Wallace A, Nichol C, Woodhouse I. Recovery of Forest Canopy Parameters by Inversion of Multispectral LiDAR Data. Remote Sens. [Internet]. Molecular Diversity Preservation International; 2012 [cited 2017 May 19];4:509–31. Available from: http://www.mdpi.com/2072-4292/4/2/509/

90. Morsy S, Shaker A, El-Rabbany A. Multispectral LiDAR Data for Land Cover Classification of Urban Areas. Sensors [Internet]. 2017 [cited 2017 May 19];17:958. Available from: http://www.ncbi.nlm.nih.gov/pubmed/28445432

91. Wallace AM, McCarthy A, Nichol CJ, Ximing Ren, Morak S, Martinez-Ramirez D, et al. Design and Evaluation of Multispectral LiDAR for the Recovery of Arboreal Parameters. IEEE Trans. Geosci. Remote Sens. [Internet]. 2014 [cited 2017 May 19];52:4942–54. Available from: http://ieeexplore.ieee.org/document/6672004/

92. Navarro P, Fernández C, Borraz R, Alonso D. A Machine Learning Approach to Pedestrian Detection for Autonomous Vehicles Using High-Definition 3D Range Data. Sensors. Multidisciplinary Digital Publishing Institute; 2016;17:18.

93. Padhi J, Misra RK, Payero JO. Estimation of soil water deficit in an irrigated cotton field with infrared thermography. F. Crop. Res. [Internet]. 2012 [cited 2017 May 12];126:45–55. Available from: http://linkinghub.elsevier.com/retrieve/pii/S0378429011003303

94. Guilioni L, Jones HG, Leinonen I, Lhomme JP. On the relationships between stomatal resistance and leaf temperatures in thermography. Agric. For. Meteorol. [Internet]. 2008 [cited 2017 May 12];148:1908–12. Available from: http://linkinghub.elsevier.com/retrieve/pii/S0168192308002074

95. Kranner I, Kastberger G, Hartbauer M, Pritchard HW. Noninvasive diagnosis of seed viability using infrared thermography. Proc. Natl. Acad. Sci. [Internet]. 2010 [cited 2017 May 12];107:3912–7. Available from: http://www.pnas.org/cgi/doi/10.1073/pnas.0914197107

96. Jones HG. Use of infrared thermography for monitoring stomatal closure in the field: application to grapevine. J. Exp. Bot. [Internet]. 2002 [cited 2017 May 12];53:2249–60. Available from: https://academic.oup.com/jxb/article-lookup/doi/10.1093/jxb/erf083

97. Fiorani F, Rascher U, Jahnke S, Schurr U. Imaging plants dynamics in heterogenic environments. Curr. Opin. Biotechnol. [Internet]. 2012 [cited 2017 May 12];23:227–35. Available from: http://www.sciencedirect.com/science/article/pii/S0958166911007531

98. Mallona I, Egea-Cortines M, Weiss J. Conserved and divergent rhythms of CAM-related and core clock gene expression in the cactus Opuntia ficus-indica. Plant Physiol. 2011;156:1978–89.

99. Somers DE, Webb a a, Pearson M, Kay S a. The short-period mutant, toc1-1, alters circadian clock regulation of multiple outputs throughout development in Arabidopsis thaliana. Development [Internet]. 1998;125:485–94. Available from: http://www.ncbi.nlm.nih.gov/pubmed/9425143

100. Costa JM, Grant OM, Chaves MM, I D, M F, RD J, et al. Thermography to explore plant-environment interactions. J. Exp. Bot. [Internet]. Oxford University Press; 2013 [cited 2017 Jan 24];64:3937–49. Available from: https://academic.oup.com/jxb/article-lookup/doi/10.1093/jxb/ert029

101. Zia S, Romano G, Spreer W, Sanchez C, Cairns J, Araus JL, et al. Infrared Thermal Imaging as a Rapid Tool for Identifying Water-Stress Tolerant Maize Genotypes of Different Phenology. J. Agron. Crop Sci. [Internet]. 2013 [cited 2017 May 12];199:75–84. Available from: http://doi.wiley.com/10.1111/j.1439-037X.2012.00537.x

102. Jones HG, Serraj R, Loveys BR, Xiong L, Wheaton A, Price AH. Thermal infrared imaging of crop canopies for the remote diagnosis and quantification of plant responses to water stress in the field. Funct. Plant Biol. [Internet]. CSIRO PUBLISHING; 2009 [cited 2017 May 12];36:978. Available from: http://www.publish.csiro.au/?paper=FP09123

103. Jones HG, Stoll M, Santos T, de Sousa C, Chaves MM, Grant OM. Use of infrared thermography for monitoring stomatal closure in the field: application to grapevine. J. Exp. Bot. Oxford University Press; 2002;53:2249–60.

104. Prashar A, Jones H. Infra-Red Thermography as a High-Throughput Tool for Field Phenotyping. Agronomy [Internet]. Multidisciplinary Digital Publishing Institute; 2014 [cited 2017 May 21];4:397–417. Available from: http://www.mdpi.com/2073-4395/4/3/397/

105. Mahlein A-K. Precision agriculture and plant phenotyping are information-and technology-based domains with specific demands and challenges for. Plant Dis. [Internet]. Plant Disease; 2016 [cited 2017 Jan 18];100:241–51. Available from: http://apsjournals.apsnet.org/doi/10.1094/PDIS-03-15-0340-FE

106. Oerke E-C, Fröhling P, Steiner U. Thermographic assessment of scab disease on apple leaves. Precis. Agric. [Internet]. Springer US; 2011 [cited 2017 May 21];12:699–715. Available from: http://link.springer.com/10.1007/s11119-010-9212-3

107. Chapple CC, Vogt T, Ellis BE, Somerville CR. An Arabidopsis mutant defective in the general phenylpropanoid pathway. Plant Cell [Internet]. American Society of Plant Biologists; 1992 [cited 2017 Jun 13];4:1413–24. Available from: http://www.ncbi.nlm.nih.gov/pubmed/1477555

108. Gould PD, Diaz P, Hogben C, Kusakina J, Salem R, Hartwell J, et al. Delayed fluorescence as a universal tool for the measurement of circadian rhythms in higher plants. Plant J. [Internet]. 2009 [cited 2011 Jul 15];58:893–901. Available from: http://www.ncbi.nlm.nih.gov/pubmed/19638147

109. Sweeney BM, Prezelin BB, Wong D, Govindjee. Invivo Chlorophyll-a Fluorescence Transients and the Circadian-Rhythm of Photosynthesis in Gonyaulax-Polyedra. Photochem. Photobiol. 1979;30:309–11.

110. Sheehan H, Moser M, Klahre U, Esfeld K, Dell’Olivo A, Mandel T, et al. MYB-FL controls gain and loss of floral UV absorbance, a key trait affecting pollinator preference and reproductive isolation. Nat. Genet. [Internet]. 2015; Available from: http://www.nature.com/doifinder/10.1038/ng.3462

111. Pérez-Bueno ML, Pineda M, Cabeza FM, Barón M. Multicolor Fluorescence Imaging as a Candidate for Disease Detection in Plant Phenotyping. Front. Plant Sci. [Internet]. Frontiers Media SA; 2016 [cited 2017 Jan 24];7:1790. Available from:

http://www.ncbi.nlm.nih.gov/pubmed/27994607

112. Fang Y, Ramasamy R. Current and Prospective Methods for Plant Disease Detection. Biosensors [Internet]. Multidisciplinary Digital Publishing Institute; 2015 [cited 2017 May 21];5:537–61. Available from: http://www.mdpi.com/2079-6374/5/3/537/

113. Boviki A. HANDBOOKOF IMAGE Am VIDEO PROCESSING.

114. Zhang Y, Wang S, Sun P. Pathological brain detection based on wavelet entropy and Hu moment invariants. Bio-medical Mater. [Internet]. 2015 [cited 2016 Oct 18]; Available from: http://content.iospress.com/articles/bio-medical-materials-and-engineering/bme1426

115. Borisjuk L, Rolletschek H, Neuberger T. Surveying the plant’s world by magnetic resonance imaging [Internet]. Plant J. 2012 [cited 2017 May 15]. p. 129–46. Available from: http://doi.wiley.com/10.1111/j.1365-313X.2012.04927.x

116. Rascher U, Blossfeld S, Fiorani F, Jahnke S, Jansen M, Kuhn AJ, et al. Non-invasive approaches for phenotyping of enhanced performance traits in bean. Funct. Plant Biol. 2011;38:968–83.

117. Horigane A, Engelaar W, Maruyama S. Visualisation of moisture distribution during development of rice caryopses (Oryza sativa L.) by nuclear magnetic resonance microimaging. J. Cereal [Internet]. 2001 [cited 2017 May 15]; Available from: http://www.sciencedirect.com/science/article/pii/S0733521000903485

118. Glidewell S. NMR imaging of developing barley grains. J. Cereal Sci. [Internet]. 2006 [cited 2017 May 15]; Available from: http://www.sciencedirect.com/science/article/pii/S0733521005000913

119. Converse A, Ahlers E, Bryan T. Positron emission tomography (PET) of radiotracer uptake and distribution in living plants: methodological aspects. Radioanal. … [Internet]. 2013 [cited 2017 May 15]; Available from: http://link.springer.com/article/10.1007/s10967-012-2383-9

120. Karve AA, Alexoff D, Kim D, Schueller MJ, Ferrieri RA, Babst BA. In vivo quantitative imaging of photoassimilate transport dynamics and allocation in large plants using a commercial positron emission tomography (PET) scanner. BMC Plant Biol. [Internet]. 2015 [cited 2017 May 15];15:273. Available from: http://www.biomedcentral.com/1471-2229/15/273

121. Yang W, Xu X, Duan L, Luo Q, Chen S, Zeng S, et al. High-throughput measurement of rice tillers using a conveyor equipped with x-ray computed tomography. Rev. Sci. Instrum. [Internet]. 2011 [cited 2017 May 15];82:25102. Available from: http://aip.scitation.org/doi/10.1063/1.3531980

122. Tracy SR, Roberts JA, Black CR, McNeill A, Davidson R, Mooney SJ. The X-factor: Visualizing undisturbed root architecture in soils using X-ray computed tomography. J. Exp. Bot. 2010;61:311–3.

123. Mooney SJ, Pridmore TP, Helliwell J, Bennett MJ. Developing X-ray computed tomography to non-invasively image 3-D root systems architecture in soil. Plant Soil. 2012;352:1–22.

124. Metzner R, Eggert A, van Dusschoten D, Pflugfelder D, Gerth S, Schurr U, et al. Direct comparison of MRI and X-ray CT technologies for 3D imaging of root systems in soil: potential and challenges for root trait quantification. Plant Methods [Internet]. 2015 [cited 2017 May 15];11:17. Available from: http://www.plantmethods.com/content/11/1/17

125. Lontoc-Roy M, Dutilleul P, Prasher SO, Han L, Brouillet T, Smith DL. Advances in the acquisition and analysis of CT scan data to isolate a crop root system from the soil medium and quantify root system complexity in 3-D space. Geoderma [Internet]. 2006 [cited 2017 May 16];137:231–41. Available from: http://www.sciencedirect.com/science/article/pii/S0016706106002576

126. Perret JS, Al-Belushi ME, Deadman M. Non-destructive visualization and quantification of roots using computed tomography. Soil Biol. Biochem. [Internet]. 2007 [cited 2017 May 16];39:391–9. Available from: http://www.sciencedirect.com/science/article/pii/S003807170600321X

127. Staedler YM, Masson D, Schönenberger J, Fischer G, Comes H. Plant Tissues in 3D via X-Ray Tomography: Simple Contrasting Methods Allow High Resolution Imaging. Sun M, editor. PLoS One [Internet]. Public Library of Science; 2013 [cited 2017 May 16];8:e75295. Available from: http://dx.plos.org/10.1371/journal.pone.0075295

128. Dhondt S, Vanhaeren H, Van Loo D, Cnudde V, Inzé D. Plant structure visualization by high-resolution X-ray computed tomography. Trends Plant Sci. [Internet]. 2010 [cited 2017 May 16];15:419–22. Available from: http://www.sciencedirect.com/science/article/pii/S1360138510000956

129. Brodersen CR, Roddy AB. New frontiers in the three-dimensional visualization of plant structure and function. Am. J. Bot. [Internet]. Botanical Society of America; 2016 [cited 2017 May 16];103:184–8. Available from: http://www.ncbi.nlm.nih.gov/pubmed/26865119

130. Jan Erik Solem. Programming Computer Vision with Python. Andy Oram, Mike Hendrikosn, editors. Program. Comput. Vis. with Python [Internet]. 1st ed. Sebastopol: O'Reilly Media; 2012;264. Available from: http://programmingcomputervision.com/

131. Navarro PJ, Pérez F, Weiss J, Egea-Cortines M. Machine learning and computer vision system for phenotype data acquisition and analysis in plants. Sensors (Switzerland) [Internet]. Multidisciplinary Digital Publishing Institute; 2016 [cited 2016 Sep 21];16:1–12. Available from: http://www.mdpi.com/1424-8220/16/5/641

132. Hamuda E, Glavin M, Jones E. A survey of image processing techniques for plant extraction and segmentation in the field [Internet]. Comput. Electron. Agric. 2016 [cited 2017 May 10]. p. 184–99. Available from: http://www.sciencedirect.com/science/article/pii/S0168169916301557

133. Krig S. Computer vision metrics: Survey, Taxonomy, and Analysis. Weiss S, Douglas S, editors. ApressOpen; 2014.

134. Wang Z, Li H, Zhu Y, Xu T. Review of Plant Identification Based on Image Processing. Arch. Comput. Methods Eng. [Internet]. 2016 [cited 2017 May 10]; Available from: http://link.springer.com/10.1007/s11831-016-9181-4

135. Bhagwat R, Dandawate Y. Indian plant species identification under varying illumination and viewpoint conditions. 2016 Conf. Adv. Signal Process. [Internet]. IEEE; 2016 [cited 2017 May 9]. p. 469–73. Available from: http://ieeexplore.ieee.org/document/7746217/

136. Thangadurai K, Padmavathi K. Computer visionimage enhancement for plant leaves disease detection. Proc. - 2014 World Congr. Comput. Commun. Technol. WCCCT 2014 [Internet]. IEEE; 2014 [cited 2017 May 10]. p. 173–5. Available from: http://ieeexplore.ieee.org/document/6755131/

137. Padmavathi K, Thangadurai K. Implementation of RGB and grayscale images in plant leaves disease detection - Comparative study. Indian J. Sci. Technol. [Internet]. 2016 [cited 2017 May 20];9. Available from: http://www.indjst.org/index.php/indjst/article/view/77739

138. Wilkinson MD, Dumontier M, Aalbersberg IjJ, Appleton G, Axton M, Baak A, et al. The FAIR Guiding Principles for scientific data management and stewardship. Sci. Data [Internet]. 2016;3:160018. Available from: http://www.pubmedcentral.nih.gov/articlerender.fcgi?artid=4792175&tool=pmcentrez&rendertype=abstract

139. Singh V, Misra AK. Detection of plant leaf diseases using image segmentation and soft computing techniques. Inf. Process. Agric. [Internet]. 2017 [cited 2017 May 30];4:41–9. Available from: http://www.sciencedirect.com/science/article/pii/S2214317316300154

140. Zhao ZQ, Ma LH, Cheung Y ming, Wu X, Tang Y, Chen CLP. ApLeaf: An efficient android-based plant leaf identification system. Neurocomputing [Internet]. 2015 [cited 2017 May 30];151:1112–9. Available from: http://www.sciencedirect.com/science/article/pii/S0925231214013368

141. Otsu N. A Threshold Selection Method from Gray-Level Histograms. IEEE Trans. Syst. Man. Cybern. [Internet]. 1979 [cited 2017 May 30];9:62–6. Available from: http://ieeexplore.ieee.org/document/4310076/

142. Liu J-C, Lin T-M. Location and Image-Based Plant Recognition and Recording System. 2015 [cited 2017 May 30];6. Available from: http://www.jihmsp.org/~jihmsp/2015/vol6/JIH-MSP-2015-05-007.pdf

143. Chéné Y, Rousseau D, Belin É tienn., Garbez M, Galopin G, Chapeau-Blondeau F. Shape descriptors to characterize the shoot of entire plant from multiple side views of a motorized depth sensor. Mach. Vis. Appl. [Internet]. Springer Berlin Heidelberg; 2016 May 19 [cited 2017 May 30];1–15. Available from: http://link.springer.com/10.1007/s00138-016-0762-x

144. Vincent L, Vincent L, Soille P. Watersheds in Digital Spaces: An Efficient Algorithm Based on Immersion Simulations. IEEE Trans. Pattern Anal. Mach. Intell. [Internet]. 1991 [cited 2017 May 30];13:583–98. Available from: http://ieeexplore.ieee.org/document/87344/

145. Patil S, Soma S, Nandyal S. Identification of Growth Rate of Plant based on leaf features using Digital Image Processing Techniques. Int. J. Emerg. Technol. Adv. Eng. Website www.ijetae.com ISO Certif. J. 2013;3.

146. Barnes C, Balzter H, Barrett K, Eddy J, Milner S, Suárez J. Individual Tree Crown

Delineation from Airborne Laser Scanning for Diseased Larch Forest Stands. Remote Sens. [Internet]. Multidisciplinary Digital Publishing Institute; 2017 [cited 2017 Apr 24];9:231. Available from: http://www.mdpi.com/2072-4292/9/3/231

147. Vukadinovic D, Polder G. Watershed and supervised classification based fully automated method for separate leaf segmentation. COST FA 1306 -The quest Toler. Var. plant Cell. Lev. Gatersleben; 2015.

148. Rother C, Kolmogorov V, Blake A. GrabCut -Interactive Foreground Extraction using Iterated Graph Cuts. ACM Trans. Graph. [Internet]. 2004. Available from: https://www.microsoft.com/en-us/research/publication/grabcut-interactive-foreground-extraction-using-iterated-graph-cuts/

149. Boykov YY, Jolly M-P. Interactive graph cuts for optimal boundary & region segmentation of objects in N-D images. Proc. Eighth IEEE Int. Conf. Comput. Vision. ICCV 2001 [Internet]. IEEE Comput. Soc; 2001 [cited 2016 Nov 3]. p. 105–12. Available from: http://ieeexplore.ieee.org/document/937505/

150. Sonka M, Hlavac V, Boyle R. Image Processing, Analysis, and Machine Vision. 3rd ed. Thomson, editor. Toronto: Thomson; 2008.

151. Wang X. The GrabCut Segmentation Technique as Used in the Study of Tree Image Extraction. In: Zhu FG and X, editor. Proc. 2009 Int. Work. Inf. Secur. Appl. (IWISA 2009). Qingdao, China: Academy Publisher; 2009.

152. Liu J-C, Chiang C-Y, Chen S. Image-Based Plant Recognition by Fusion of Multimodal Information. 2016 10th Int. Conf. Innov. Mob. Internet Serv. Ubiquitous Comput. [Internet]. IEEE; 2016 [cited 2017 May 30]. p. 5–11. Available from: http://ieeexplore.ieee.org/document/7794433/

153. Liu J-C, Lin T-M. Location and Image-Based Plant Recognition and Recording System. 2015;6. Available from: http://www.jihmsp.org/~jihmsp/2015/vol6/JIH-MSP-2015-05-007.pdf

154. Kass M, Witkin A, Terzopoulos D. Snakes: Active contour models. Int. J. Comput. Vis. [Internet]. Kluwer Academic Publishers; 1988 [cited 2016 Nov 5];1:321–31. Available from: http://link.springer.com/10.1007/BF00133570

155. Minervini M, Abdelsamea MM, Tsaftaris SA. Image-based plant phenotyping with incremental learning and active contours. Ecol. Inform. 2014;23:35–48.

156. Suta L, Bessy F, Veja C, Vaida M-F. Active contours: Application to plant

recognition. 2012 IEEE 8th Int. Conf. Intell. Comput. Commun. Process. [Internet]. IEEE; 2012 [cited 2016 Nov 5]. p. 181–7. Available from: http://ieeexplore.ieee.org/document/6356183/

157. Shi Y, Karl WC. A real-time algorithm for the approximation of level-set-based curve evolution. IEEE Trans. Image Process. [Internet]. 2008 [cited 2016 Nov 5];17:645–56. Available from: http://ieeexplore.ieee.org/document/4480128/

158. Chan TF, Vese LA. Active contours without edges. IEEE Trans. Image Process. [Internet]. IEEE Press; 2001 [cited 2016 Nov 5];10:266–77. Available from: http://ieeexplore.ieee.org/document/902291/

159. Pape J-M, Klukas C. Utilizing machine learning approaches to improve the prediction of leaf counts and individual leaf segmentation of rosette plant images. Proc. Comput. Vis. Probl. Plant Phenotyping [Internet]. British Machine Vision Association; 2015 [cited 2016 Sep 21];1–12. Available from: http://www.bmva.org/bmvc/2015/cvppp/papers/paper003/index.html

160. Arivazhagan S, Shebiah RN, Ananthi S, Vishnu Varthini S. Detection of unhealthy region of plant leaves and classification of plant leaf diseases using texture features. Agric. Eng. Int. CIGR J. 2013;15:211–7.

161. Mouille G, Robin S, Lecomte M, Pagant S, Höfte H. Classification and identification of *Arabidopsis* cell wall mutants using Fourier-Transform InfraRed (FT-IR) microspectroscopy. Plant J. [Internet]. Blackwell Science Ltd; 2003 [cited 2017 May 27];35:393–404. Available from: http://doi.wiley.com/10.1046/j.1365-313X.2003.01807.x

162. Guijarro M, Riomoros I, Pajares G, Zitinski P. Discrete wavelets transform for improving greenness image segmentation in agricultural images. Comput. Electron. Agric. 2015;118:396–407.

163. Iyer-Pascuzzi AS, Symonova O, Mileyko Y, Hao Y, Belcher H, Harer J, et al. Imaging and analysis platform for automatic phenotyping and trait ranking of plant root systems. Plant Physiol. [Internet]. American Society of Plant Biologists; 2010 [cited 2017 May 28];152:1148–57. Available from: http://www.ncbi.nlm.nih.gov/pubmed/20107024

164. Lowe DG. Distinctive Image Features from Scale-Invariant Keypoints. Int. J. Comput. Vis. [Internet]. Kluwer Academic Publishers; 2004 [cited 2016 Dec 7];60:91–

110. Available from: http://link.springer.com/10.1023/B:VISI.0000029664.99615.94

165. Bay H, Ess A, Tuytelaars T, Van Gool L. Speeded-Up Robust Features (SURF). Comput. Vis. Image Underst. 2008;110:346–59.

166. Dalal N, Triggs B. Histograms of Oriented Gradients for Human Detection. 2005 IEEE Comput. Soc. Conf. Comput. Vis. Pattern Recognit. [Internet]. IEEE; [cited 2016 Dec 9]. p. 886–93. Available from: http://ieeexplore.ieee.org/document/1467360/

167. Guo W, Fukatsu T, Ninomiya S. Automated characterization of flowering dynamics in rice using field-acquired time-series RGB images. Plant Methods [Internet]. 2015 [cited 2017 May 28];11:7. Available from: http://www.plantmethods.com/content/11/1/7

168. Santos T, Oliveira A. Image-based 3D digitizing for plant architecture analysis and phenotyping. … SIBGRAPI 2012 (XXV Conf. … [Internet]. 2012 [cited 2016 Dec 8]; Available from: http://www.cnptia.embrapa.br/~thiago/pool/2012-08-24_sibgrapi.pdf

169. Roscher R, Herzog K, Kunkel A, Kicherer A, T??pfer R, F??rstner W. Automated image analysis framework for high-throughput determination of grapevine berry sizes using conditional random fields. Comput. Electron. Agric. [Internet]. 2014 [cited 2017 May 29];100:148–58. Available from: http://www.sciencedirect.com/science/article/pii/S0168169913002780

170. Lantz B. Machine Learning with R. 1st ed. Jones J, Sheikh A, editors. Birmingham: Packt Publishing; 2013.

171. Müller A, Guido S. Introduction to Machine Learning with Python. 1st ed. Schanafelt D, editor. Sebastopol: O'Reilly Media; 2016.

172. Smola A, Vishwanathan SV. Introduction to Machine Learning. 1st ed. Cambridge: Cambridge University Press; 2008.

173. Baranowski P, Jedryczka M, Mazurek W, Babula-Skowronska D, Siedliska A, Kaczmarek J. Hyperspectral and thermal imaging of oilseed rape (Brassica napus) response to fungal species of the genus Alternaria. Wilson RA, editor. PLoS One [Internet]. Public Library of Science; 2015 [cited 2016 Nov 6];10:e0122913. Available from: http://dx.plos.org/10.1371/journal.pone.0122913

174. Fukushima K. Neocognitron: a self organizing neural network model for a mechanism of pattern recognition unaffected by shift in position. Biol. Cybern. [Internet]. 1980 [cited 2016 Nov 6];36:193–202. Available from:

http://www.ncbi.nlm.nih.gov/pubmed/7370364

175. Pound MP, Burgess AJ, Wilson MH, Atkinson JA, Griffiths M, Jackson AS, et al. Deep Machine Learning provides state-of-the-art performance in image-based plant phenotyping. bioRxiv [Internet]. 2016 [cited 2016 Sep 21];53033. Available from: http://biorxiv.org/lookup/doi/10.1101/053033

176. Mohanty SP, Hughes D, Salathé M. Using Deep Learning for Image-Based Plant Disease Detection. 2016;1–7.

177. Tsaftaris SA, Minervini M, Scharr H. Machine Learning for Plant Phenotyping Needs Image Processing [Internet]. Trends Plant Sci. 2016 [cited 2017 May 27]. p. 989–91. Available from: http://linkinghub.elsevier.com/retrieve/pii/S1360138516301613

178. Naik HS, Zhang J, Lofquist A, Assefa T, Sarkar S, Ackerman D, et al. A real-time phenotyping framework using machine learning for plant stress severity rating in soybean. Plant Methods [Internet]. 2017 [cited 2017 May 29];13:23. Available from: http://plantmethods.biomedcentral.com/articles/10.1186/s13007-017-0173-7

179. Nagler PL, Inoue Y, Glenn E., Russ A., Daughtry CS. Cellulose absorption index (CAI) to quantify mixed soil–plant litter scenes. Remote Sens. Environ. 2003;87:310–25.

180. Ren H, Zhou G, Zhang F, Zhang X. Evaluating cellulose absorption index (CAI) for non-photosynthetic biomass estimation in the desert steppe of Inner Mongolia. Chinese Sci. Bull. SP Science China Press; 2012;57:1716–22.

181. Serbin G, Daughtry CST, Hunt ER, Reeves JB, Brown DJ. Effects of soil composition and mineralogy on remote sensing of crop residue cover. Remote Sens. Environ. 2009;113:224–38.

182. Eskandari I, Navid H, Rangzan K. Evaluating spectral indices for determining conservation and conventional tillage systems in a vetch-wheat rotation. Int. Soil Water Conserv. Res. 2016;4:93–8.

183. Galvão LS, Formaggio AR, Tisot DA. Discrimination of sugarcane varieties in Southeastern Brazil with EO-1 Hyperion data. Remote Sens. Environ. 2005;94:523–34.

184. Price J. Leaf area index estimation from visible and near-infrared reflectance data. Remote Sens. Environ. 1995;52:55–65.

185. Zarco-Tejada P, Berjón A, Miller J. Stress detection in crops with hyperspectral remote sensing and physical simulation models. Proc. Airborne. 2004;

186. Cai J, Golzarian MR, Miklavcic SJ. Novel Image Segmentation Based on Machine Learning and Its Application to Plant Analysis. Int. J. Inf. Electron. Eng. 2011;1.

187. Gong P, Pu R, Biging G. Estimation of forest leaf area index using vegetation indices derived from Hyperion hyperspectral data. IEEE Trans. 2003;

188. Brown HE, Diuk-Wasser MA, Guan Y, Caskey S, Fish D. Comparison of three satellite sensors at three spatial scales to predict larval mosquito presence in Connecticut wetlands. Remote Sens. Environ. 2008;112:2301–8.

189. Apan A, Held A, Phinn S, Markley J. Detecting sugarcane “orange rust” disease using EO-1 Hyperion hyperspectral imagery. Int. J. Remote Sens. Taylor & Francis Group ; 2004;25:489–98.

190. Tucker CJ, Slayback DA, Pinzon JE, Los SO, Myneni RB, Taylor MG. Higher northern latitude normalized difference vegetation index and growing season trends from 1982 to 1999. Int. J. Biometeorol. Springer-Verlag; 2001;45:184–90.

191. Haboudane D, Miller JR, Pattey E, Zarco-Tejada PJ, Strachan IB. Hyperspectral vegetation indices and novel algorithms for predicting green LAI of crop canopies: Modeling and validation in the context of precision agriculture. Remote Sens. Environ. 2004;90:337–52.

192. Pagani A, Echeverría HE, Andrade FH, Sainz Rozas HR. Characterization of Corn Nitrogen Status with a Greenness Index under Different Availability of Sulfur. Agron. J. American Society of Agronomy; 2009;101:315.

193. Blackburn GA. Spectral indices for estimating photosynthetic pigment concentrations: A test using senescent tree leaves. Int. J. Remote Sens. Taylor & Francis Group ; 1998;19:657–75.

194. Hunt ER, Dean Hively W, Fujikawa SJ, Linden DS, Daughtry CST, McCarty GW. Acquisition of NIR-green-blue digital photographs from unmanned aircraft for crop monitoring. Remote Sens. Molecular Diversity Preservation International; 2010;2:290–305.

195. Bell GE, Howell BM, Johnson GV, Raun WR, Solie JB, Stone ML. Optical Sensing of Turfgrass Chlorophyll Content and Tissue Nitrogen. HortScience. American Society for Horticultural Science; 2004;39:1130–2.

196. Haboudane D, Miller JR, Tremblay N, Zarco-Tejada PJ, Dextraze L. Integrated narrow-band vegetation indices for prediction of crop chlorophyll content for

application to precision agriculture. Remote Sens. Environ. 2002;81:416–26.

197. Timm BC, McGarigal K. Fine-scale remotely-sensed cover mapping of coastal dune and salt marsh ecosystems at Cape Cod National Seashore using Random Forests. Remote Sens. Environ. 2012;127:106–17.

198. Parenteau MP, Bannari A, El-Harti A, Bachaoui M, El-Ghmari A. Characterization of the state of soil degradation by erosion using the hue and coloration indices. IGARSS 2003. 2003 IEEE Int. Geosci. Remote Sens. Symp. Proc. (IEEE Cat. No.03CH37477). IEEE; p. 2284–6.

199. Pajares G, Ruz JJ, de la Cruz JM. Performance analysis of homomorphic systems for image change detection. Pattern Recognit. Image Anal. Pt 1 [Internet]. Springer, Berlin, Heidelberg; 2005 [cited 2017 Jul 25]. p. 563–70. Available from: http://link.springer.com/10.1007/11492429_68

200. Perez AJ, Lopez F, Benlloch J V., Christensen S. Colour and shape analysis techniques for weed detection in cereal fields. Comput. Electron. Agric. [Internet]. 2000 [cited 2017 Jul 25];25:197–212. Available from: http://linkinghub.elsevier.com/retrieve/pii/S016816999900068X

201. Oluleye B, Leisa A, Jinsong L, Dean D. On the Application of Genetic Probabilistic Neural Networks and Cellular Neural Networks in Precision Agriculture. Asian J. Comput. Inf. Syst. [Internet]. 2014 [cited 2017 Aug 8];2:90–101. Available from: http://ro.ecu.edu.au/ecuworkspost2013/677

202. Knoll FJ, Holtorf T, Hussmann S. Modified 3D time-of-flight camera for object separation in organic farming. In: Kress BC, Osten W, Urbach HP, editors. International Society for Optics and Photonics; 2017 [cited 2017 Aug 9]. p. 103351R. Available from: http://proceedings.spiedigitallibrary.org/proceeding.aspx?doi=10.1117/12.2270276

203. Huang K-Y. Application of artificial neural network for detecting Phalaenopsis seedling diseases using color and texture features. Comput. Electron. Agric. [Internet]. 2007 [cited 2017 Sep 11];57:3–11. Available from: http://linkinghub.elsevier.com/retrieve/pii/S0168169907000385

204. Mokhtar U, Ali MAS, Hassanien AE, Hefny H. Identifying Two of Tomatoes Leaf Viruses Using Support Vector Machine. Springer, New Delhi; 2015 [cited 2017 Sep 11]. p. 771–82. Available from: http://link.springer.com/10.1007/978-81-322-2250-7_77

205. Casanova J, O'Shaughnessy S, Evett S, Rush C. Development of a Wireless

Computer Vision Instrument to Detect Biotic Stress in Wheat. Sensors [Internet]. Multidisciplinary Digital Publishing Institute; 2014 [cited 2017 Sep 11];14:17753–69. Available from: http://www.mdpi.com/1424-8220/14/9/17753/

206. Bauer SD, Korč F, Förstner W. The potential of automatic methods of classification to identify leaf diseases from multispectral images. Precis. Agric. [Internet]. Springer US; 2011 [cited 2017 Sep 11];12:361–77. Available from: http://link.springer.com/10.1007/s11119-011-9217-6

207. Atkinson JA, Lobet G, Noll M, Meyer PE, Griffiths M, Wells DM. Combining semi-automated image analysis techniques with machine learning algorithms to accelerate large scale genetic studies. bioRxiv [Internet]. 2017 [cited 2017 Aug 8]; Available from: http://www.biorxiv.org/content/early/2017/06/20/152702

208. Ballabeni A, Apollonio FI, Gaiani M, Remondino F. Advances in image pre-processing to improve automated 3d reconstruction. Int. Arch. Photogramm. Remote Sens. Spat. Inf. Sci. - ISPRS Arch. [Internet]. 2015 [cited 2017 Jul 20]. p. 315–23. Available from: http://www.int-arch-photogramm-remote-sens-spatial-inf-sci.net/XL-5-W4/315/2015/

209. Klodt M, Herzog K, Töpfer R, Cremers D. Field phenotyping of grapevine growth using dense stereo reconstruction. BMC Bioinformatics [Internet]. BioMed Central; 2015 [cited 2017 Jul 20];16:143. Available from: http://www.ncbi.nlm.nih.gov/pubmed/25943369

210. Zhang X, Huang C, Wu D, Qiao F, Li W, Duan L, et al. High-throughput phenotyping and QTL mapping reveals the genetic architecture of maize plant growth. Plant Physiol. [Internet]. 2017;pp.01516.2016. Available from: http://www.plantphysiol.org/lookup/doi/10.1104/pp.16.01516

211. Tsung-Shiang H. An Improvement Stereo Vision Images Processing for Object Distance Measurement. Int. J. Autom. Smart Technol. [Internet]. 2015 [cited 2017 Aug 9];5:85–90. Available from: http://www.ausmt.org/index.php/AUSMT/article/view/460

212. Raza SEA, Smith HK, Clarkson GJJ, Taylor G, Thompson AJ, Clarkson J, et al. Automatic detection of regions in spinach canopies responding to soil moisture deficit using combined visible and thermal imagery. Merks RMH, editor. PLoS One [Internet]. Public Library of Science; 2014 [cited 2017 Aug 9];9:e97612. Available from:

http://dx.plos.org/10.1371/journal.pone.0097612

213. Raza SEA, Prince G, Clarkson JP, Rajpoot NM. Automatic detection of diseased tomato plants using thermal and stereo visible light images. Perovic D, editor. PLoS One [Internet]. Narosa Publishing House; 2015 [cited 2017 Aug 9];10:e0123262. Available from: http://dx.plos.org/10.1371/journal.pone.0123262

214. Kazmi W, Foix S, Alenyà G, Andersen HJ. Indoor and outdoor depth imaging of leaves with time-of-flight and stereo vision sensors: Analysis and comparison. ISPRS J. Photogramm. Remote Sens. [Internet]. 2014 [cited 2017 Aug 9];88:128–46. Available from: http://linkinghub.elsevier.com/retrieve/pii/S0924271613002748

215. Wahabzada M, Mahlein A-K, Bauckhage C, Steiner U, Oerke E-C, Kersting K. Plant Phenotyping using Probabilistic Topic Models: Uncovering the Hyperspectral Language of Plants. Sci. Rep. [Internet]. Nature Publishing Group; 2016 [cited 2016 Oct 18];6:22482. Available from: http://www.nature.com/articles/srep22482

216. Ochoa D, Cevallos J, Vargas G, Criollo R, Romero D, Castro R, et al. Hyperspectral imaging system for disease scanning on banana plants. Kim MS, Chao K, Chin BA, editors. Proc. SPIE 9864, Sens. Agric. Food Qual. Saf. VIII [Internet]. International Society for Optics and Photonics; 2016 [cited 2017 Jul 20];4:98640M. Available from: http://proceedings.spiedigitallibrary.org/proceeding.aspx?doi=10.1117/12.2224242

217. Mahlein A-K, Kuska MT, Thomas S, Bohnenkamp D, Alisaac E, Behmann J, et al. Plant disease detection by hyperspectral imaging: from the lab to the field. Adv. Anim. Biosci. [Internet]. 2017 [cited 2017 Jul 20];8:238–43. Available from: https://www.cambridge.org/core/product/identifier/S2040470017001248/type/journal_article

218. Pandey P, Ge Y, Stoerger V, Schnable JC. High Throughput In vivo Analysis of Plant Leaf Chemical Properties Using Hyperspectral Imaging. Front. Plant Sci. [Internet]. Frontiers; 2017 [cited 2017 Aug 9];8:1348. Available from: http://journal.frontiersin.org/article/10.3389/fpls.2017.01348/full

219. Wahabzada M, Mahlein AK, Bauckhage C, Steiner U, Oerke EC, Kersting K. Metro maps of plant disease dynamics-automated mining of differences using hyperspectral images. Lightfoot DA, editor. PLoS One [Internet]. Springer; 2015 [cited 2017 Sep 11];10:e0116902. Available from: http://dx.plos.org/10.1371/journal.pone.0116902

220. Chattopadhyay S, Akbar SA, Elfiky NM, Medeiros H, Kak A. Measuring and

modeling apple trees using time-of-flight data for automation of dormant pruning applications. 2016 IEEE Winter Conf. Appl. Comput. Vision, WACV 2016 [Internet]. IEEE; 2016 [cited 2017 Sep 4]. p. 1–9. Available from: http://ieeexplore.ieee.org/document/7477596/

221. Chéné Y, Rousseau D, Lucidarme P, Bertheloot J, Caffier V, Morel P, et al. On the use of depth camera for 3D phenotyping of entire plants. Comput. Electron. Agric. [Internet]. 2012 [cited 2017 Sep 4];82:122–7. Available from: http://linkinghub.elsevier.com/retrieve/pii/S016816991100319X

222. Weiss U, Biber P, Laible S, Bohlmann K, Zell A. Plant species classification using a 3D LIDAR sensor and machine learning. Proc. - 9th Int. Conf. Mach. Learn. Appl. ICMLA 2010 [Internet]. IEEE; 2010 [cited 2017 Aug 8]. p. 339–45. Available from: http://ieeexplore.ieee.org/document/5708854/

223. Greaves HE, Vierling LA, Eitel JUH, Boelman NT, Magney TS, Prager CM, et al. Applying terrestrial lidar for evaluation and calibration of airborne lidar-derived shrub biomass estimates in Arctic tundra. Remote Sens. Lett. [Internet]. Taylor & Francis; 2017 [cited 2017 Aug 8];8:175–84. Available from: https://www.tandfonline.com/doi/full/10.1080/2150704X.2016.1246770

224. Paulus S, Dupuis J, Mahlein A-K, Kuhlmann H. Surface feature based classification of plant organs from 3D laserscanned point clouds for plant phenotyping. BMC Bioinformatics [Internet]. 2013 [cited 2017 Sep 11];14:238. Available from: http://bmcbioinformatics.biomedcentral.com/articles/10.1186/1471-2105-14-238

225. Liu S, Baret F, Abichou M, Boudon F, Thomas S, Zhao K, et al. Estimating wheat green area index from ground-based LiDAR measurement using a 3D canopy structure model. Agric. For. Meteorol. [Internet]. 2017 [cited 2017 Aug 8];247:12–20. Available from: http://linkinghub.elsevier.com/retrieve/pii/S016819231730223X

226. Lopatin J, Dolos K, Hernández HJ, Galleguillos M, Fassnacht FE. Comparing Generalized Linear Models and random forest to model vascular plant species richness using LiDAR data in a natural forest in central Chile. Remote Sens. Environ. [Internet]. 2016 [cited 2017 Aug 8];173:200–10. Available from: http://linkinghub.elsevier.com/retrieve/pii/S0034425715302169

227. García-Tejero IF, Hernández A, Padilla-Díaz CM, Diaz-Espejo A, Fernández J. Assessing plant water status in a hedgerow olive orchard from thermography at plant

level. Agric. Water Manag. [Internet]. 2017 [cited 2017 Sep 6];188:50–60. Available from: http://linkinghub.elsevier.com/retrieve/pii/S0378377417301269

228. Deery DM, Rebetzke GJ, Jimenez-Berni JA, James RA, Condon AG, Bovill WD, et al. Methodology for High-Throughput Field Phenotyping of Canopy Temperature Using Airborne Thermography. Front. Plant Sci. [Internet]. Frontiers Media SA; 2016 [cited 2017 Sep 6];7:1808. Available from: http://www.ncbi.nlm.nih.gov/pubmed/27999580

229. Rousseau C, Belin E, Bove E, Rousseau D, Fabre F, Berruyer R, et al. High throughput quantitative phenotyping of plant resistance using chlorophyll fluorescence image analysis. Plant Methods [Internet]. 2013 [cited 2017 Sep 6];9:17. Available from: http://plantmethods.biomedcentral.com/articles/10.1186/1746-4811-9-17

230. Wetterich CB, Felipe de Oliveira Neves R, Belasque J, Ehsani R, Marcassa LG. Detection of Huanglongbing in Florida using fluorescence imaging spectroscopy and machine-learning methods. Appl. Opt. [Internet]. Optical Society of America; 2017 [cited 2017 Sep 7];56:15. Available from: https://www.osapublishing.org/abstract.cfm?URI=ao-56-1-15

231. Hillnhutter C, Sikora RA, Oerke E-C, van Dusschoten D. Nuclear magnetic resonance: a tool for imaging belowground damage caused by Heterodera schachtii and Rhizoctonia solani on sugar beet. J. Exp. Bot. [Internet]. Kluwer Academic Publishers, Wageningen, The Netherlands; 2012 [cited 2017 Sep 5];63:319–27. Available from: https://academic.oup.com/jxb/article-lookup/doi/10.1093/jxb/err273

232. Yang W, Xu X, Duan L, Luo Q, Chen S, Zeng S, et al. High-throughput measurement of rice tillers using a conveyor equipped with x-ray computed tomography. Rev. Sci. Instrum. [Internet]. American Institute of Physics; 2011 [cited 2017 Sep 5];82:25102. Available from: http://aip.scitation.org/doi/10.1063/1.3531980

233. Donis-González IR, Guyer DE, Fulbright DW, Pease A. Postharvest noninvasive assessment of fresh chestnut (Castanea spp.) internal decay using computer tomography images. Postharvest Biol. Technol. [Internet]. 2014 [cited 2017 Sep 7];94:14–25. Available from: http://linkinghub.elsevier.com/retrieve/pii/S0925521414000805

Tables

Table 1. List of software tools for image processing

Vision libraries	Source	Language
OpenCV<br>EmguCV	http://opencv.org<br>http://www.emgu.com/	C++, Python, Java, C#
PlantCV<br>Scikit-image	*http://plantcv.danforthcenter.org*<br>*http://scikit-image.org*	Python
Bioimagetools, bayesimages, edci, DRIP, dpmixsim, raster, ...	https://cran.r-project.org/	R
Cimg<br>Simplecv<br>Fastcv<br><br>Ccv<br>Vxl	*http://cimg.eu*<br>*http://*Simplecv.org<br>https://developer.qualcomm.com/software/fastcv-sdk<br>*http://*libccv.org<br>*http://*vxl.sourceforge.net	C++
BoofCV<br>OpenIMAJ<br>JavaCV	http://boofcv.org<br>http://openimaj.org<br>https://github.com/bytedeco/javacv	Java

Table 2. A list of indexes, the corresponding wavelength ranges and their use to analyse plant material.

Index	Range nm	Applications
**CAI** – Cellulose Absorption Index	2200-2000	Quantification mixed soil–plant litter scenes [179], estimation of non-photosynthetic biomass [180]
**LCA** – Lignin-Cellulose Absorption Index	2365-2145	Measure the effects of soil composition and mineralogy of crop residue cover [181]
**NTDI** – Normalized Difference Tillage Index	2359-1150	Used for identifying crop residue cover in conventional and conservation tillage systems [182]
**LWVI-1** – Normalized Difference Leaf water VI 2	1094-893	Discrimination of sugarcane varieties, allowed to detect large amounts of non photosynthetically-active constituents within the canopy [183]
**DLAI** – Difference Leaf Area Index	1725-970	Used for estimating leaf area index based on the radiation measurements in the visible and near-infrared [184]
**PWI** – Plant Water Index	970-902	Water content estimation and study of the characteristics of canopy spectrum and growth status [185][186]
**NLI** – Nonlinear vegetation index	1400-780	Measurement of plant leaf water content. In combination with others indexes can detect interaction of biochemicals such as protein, nitrogen, lignin, cellulose, sugar, and starch [187]
**DWSI** – Disease water stress index	1657-547	To predict larval mosquito presence in wetland [188]and detect sugarcane 'orange rust' disease [189]
**NDVI** – Normalized Difference Vegetation Index	800-670	Measurement significant variations in photosynthetic activity and growing season length at different latitudes [190]
**MCARI** – Modified Chlorophyll Absorption Ratio Index	700-670	Study of vegetation biophysical parameters, as well as to external factors affecting canopy reflectance [191]
**GI** – Greenness Index	670-550	Characterization of corn nitrogen status [192]
**CAR** – Chlorophyll absorption ratio	700-500	Estimating the concentration of individual photosynthetic pigments within vegetation [193]
**GNDVI** – Green normalized difference vegetation index	800-550	Providing important information for site-specific agricultural decision making [194] and for identification of chlorophyll content and tissue nitrogen [195]
**OSAVI** – Optimized Soil Adjusted Vegetation Index	800-670	Measurement with high sensitive of chlorophyll content variations and very resistant to the variations of LAI and solar zenith angle [196]
**CI r** – Coloration Index red	780-710	Mapping of coastal dune and salt marsh ecosystems [197]
**CI g** – Coloration Index green	780-550	Characterization of the state of soil degradation by erosion [198]

Table 3. List of Machine Learning software libraries and their languages

**Libraries ML/DL**	**Source**	**Language**
MICE, rpart, Party, CARET, randomForest, nnet, e1071, KernLab, igraph, glmnet, ROCR, tree, Rweka, earth, klaR,	https://cran.r-project.org/	R
Scikit-learn<br>Tensorflow<br>Theano<br>Pylearn2,<br>NuPIC<br>Caffe<br>PyBrain	http://scikit-learn.org/stable/<br>*https://www.tensorflow.org/*<br>*http://deeplearning.net/software/theano*<br>*http://deeplearning.net/software/pylearn2*<br>*http://numenta.org/*<br>*http://caffe.berkeleyvision.org/*<br>*http://pybrain.org/*	Python
Weka<br>Spark<br>Mallet<br>JSAT<br>ELKI<br>Java-ML	http://www.cs.waikato.ac.nz/ml/weka/<br>http://spark.apache.org/<br>http://mallet.cs.umass.edu/<br>https://github.com/EdwardRaff/JSAT<br>http://elki.dbs.ifi.lmu.de/<br>http://java-ml.sourceforge.net/	Java
Accord<br>Multiboost<br>Shogun<br>LibSVM<br>mlpack<br>Shark<br>MLC++	http://accord-framework.net/<br>http://www.multiboost.org/<br>http://shogun-toolbox.org/<br>http://www.csie.ntu.edu.tw/~cjlin/libsvm/<br>http://mlpack.org/<br>http://image.diku.dk/shark/<br>http://www.sgi.com/tech/mlc/source.html	C#, C++, C

Table 4 A list of current procedures procedures for image analysis based on the type of sensor used.

Data type /Source	Pre-processing	Segmentation	Feature extraction	Machine Learning
Mono – RGB	* Homomorphic filtering to minimize illumination issues in outdoor images [199]<br><br>* Filtering and histogram equalization in plant disease detection [136]	* Many vegetation indexes apply to segmentation in [132]<br><br>* NDVI index to discriminate background foreground [200]<br><br>* Cellular neural networks edge detection [201]<br><br>* HSV-algorithm [202]	* Fourier descriptors and Zernike moments [201]<br><br>* Statistical parameters and Wavelet transform with geometric characteristics [131]<br><br>* SIFT and SURF in 3D reconstruction images from multiple RGB cameras with basil specimen [168]<br><br>* Histogram to color features and Fast Fourier Transform + Discrete Wavelet Transform to texture features extraction [145]	* ANN to detect Phalaenopsis seedling diseases [203]<br><br>* SVM to detect tomatoes leaf viruses [204]<br><br>* Gaussian mixture model to detect biotic stress in wheat [205]<br><br>* k-NN to identify leaf disease [206]<br><br>* Probabilistic Neural Networks and Genetic Algorithm [201]<br><br>* Random forest to QTL analysis [207]
StereoVision	*Complete and general pre-processing pipeline [208]<br><br>* Rectification of image based on SIFT and epipolar transformation, in vitis vinifera segmentation [209]<br><br>* Camera stereo calibration, leaf quantifying Brassica napus [210]<br><br>* RGB2GrayScale [211]<br>* Align and depth estimation [212,213]	* Otsu's method & growing region [211]<br><br>* SVM to remove background [213]	* Graph-cut and local correlation [214]<br><br>* SURF to stereo matching images base on theirs feature vectors [211]<br><br>* Combined with thermal images (Global and local features (temperature, depth, color) using PCA and ANOVA [213]<br><br>* Simple statistical and intensity values [212]	* SVM identify diseased pixels in leaves [213]<br><br>* SVM & Gaussian Processes Classifier (GPC) to detect soil moisture deficit [212]
Multi-Hyper spectral	* Savitzky-Golay filter: remove noise and smooth the image. [215]<br><br>* Gaussian filter to	* NDVI (750-705/750+705) nm with threshold of 0.20 [218]	* Pixels averaged to obtain average reflectance [218]	* Cascade of data mining techniques to detect foliar disease in barley leaves [219]

	remove noise: detection of disease in banana leaves [216]<br><br>* Savitzky-Golay filter: detection of disease in plants [217]			* Bayes, logistic, random forest and decision trees to detect biotic stress in Alternaria genus [173]<br><br>* k-NN to identify leaf disease [206]<br><br>* PCA and partial least squares regression to predict water, macro and micronutrients concentrations [218]
ToF	* Correction of the distance error caused by the extra contribution of electrons from sunlight using an offset parameter [68]<br><br>* Carry-out a calibration stage before to fuse the depth data and color information [69,70]<br><br>* Removal spurious individual points (outliers) using statistical filter [74]<br><br>* Removal of lens distortion [220]	* Combine hierarchical color segmentation with quadratic surface fitting using ToF depth data [70]<br><br>* The maximally stable extremal regions algorithm for the segmentation of single object over background in gray level images [221]<br><br>* Removal background by simple thresholding pixel values greater than a certain threshold [220]<br><br>*Segmentation inspired from the maximally stable extremal regions algorithm [221]	* SIFT, Hough Transform and RANSAC algorithm to extract relevant features [220]	
LIDAR	* RANSAC algorithm to detect ground plane [222]<br><br>* Reduction of noise, filtering points clouds based on deviation [223]	* Clustering to detect individual plants [222]	* Statistical features from reflectace and geometry [222]<br><br>* Surface feature histograms to characterize the grapevine and wheat organs [224]	* ANN for wheat green area index measurement [225]<br><br>* ANN, SVM, logistic regression to plant identification (the best results) [222]<br><br>* Generalized linear model (the best) to model plant richness [226]<br><br>* SVM obtained

				highly reliable classification about 96% [224]
Thermography / Flourescence	*Align with stereo images (In combination with stereo images) [212,213]<br><br>* Normalize thermal information with thermal indexes [227]<br><br>* Trimming extraneous images from image stack [228]	* Semi automated segmentation through a geometric algorithm implemented in Python-based software ChopIt [228]<br><br>* Manual thresholding comparing conventional color images with fluorescences images (Fv/Fm) [229]	* Combined with thermal images (Global and local features: temperature, depth, color) using PCA and ANOVA [213]	* SVM identify diseased pixels in leaves [213]<br><br>* SVM and Gaussian Processes Classifier (GPC) to detect soil moisture deficit [212]<br><br>* ANOVA (not ML) to analyze different water status [227]<br><br>* ANN and SVM to detect zinc-deficiency stress using fluorescence imaging [230]
MRI/ Tomography	* 2D and 3D Fourier transformations (MRI) [231]<br><br>* Median filter, binaryzation, fill holes, remove small particle and morphological filter (erosion) [232]<br><br>* Re-slicing, cropping and contrast enhancement [233]	* Yang2011: watershed segmentation [232]<br><br>* Histogram thresholding method to binaryze the image [233]	* Intensity features, Haralick textural features, intensity local binary pattern features, contrast features and Gabor intensity textural features [233]	* Supervised learning with ANN, Mahalanobis distance, Linear discriminant analysis and quadratic discriminant analysis to determine boundary lines [233]

Figure Legends

Figure 1. Basic workflow in computer vision-based plant phenotyping

Figure 2. An overview of different spectra used for phenotyping and the associated cameras. Names of different indexes are found in Table 2.

Figure 1 Click here to download Figure figure1GigascienceV2.png

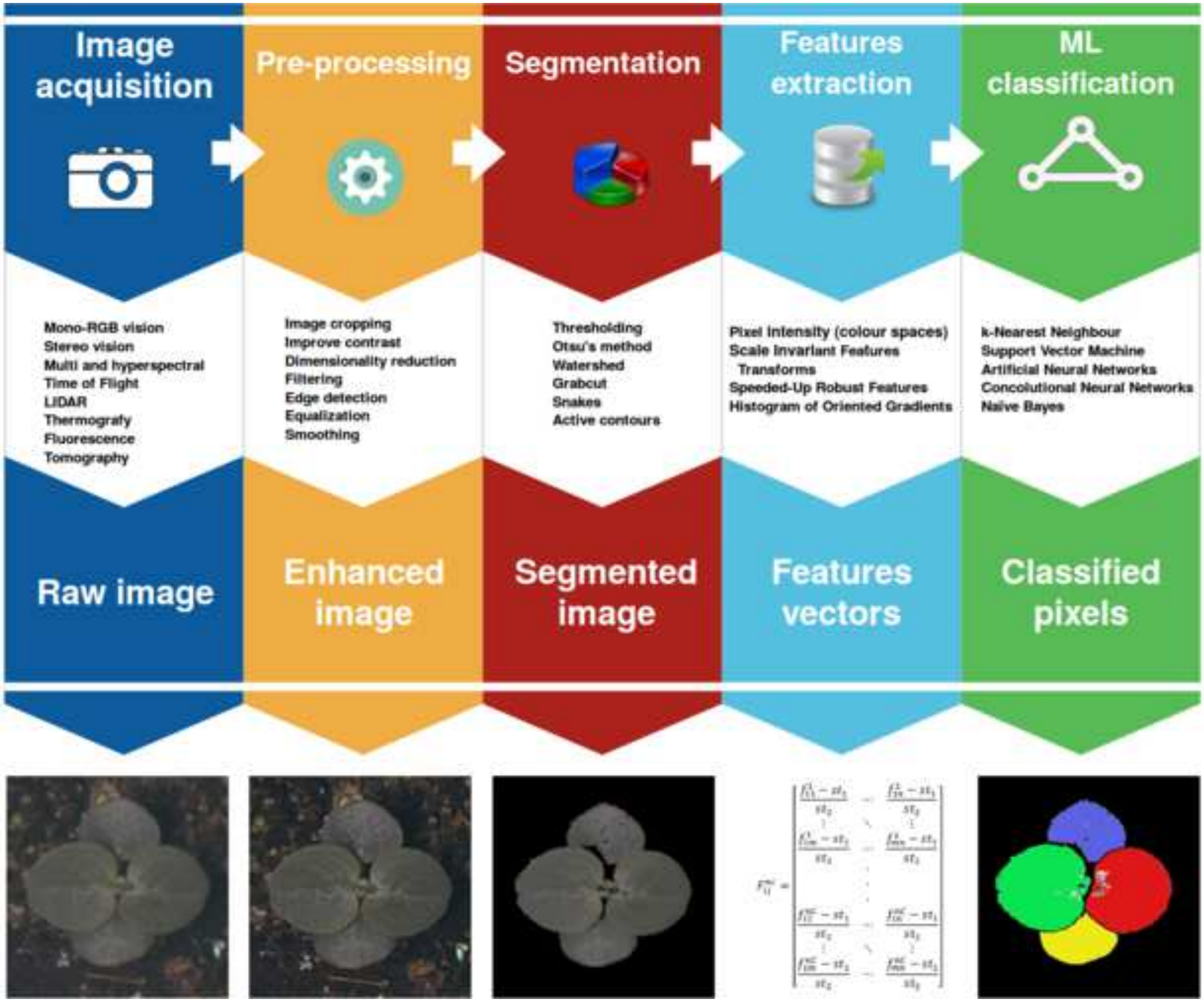

Figure 2

Click here to download Figure figure2Gigascience.pdf

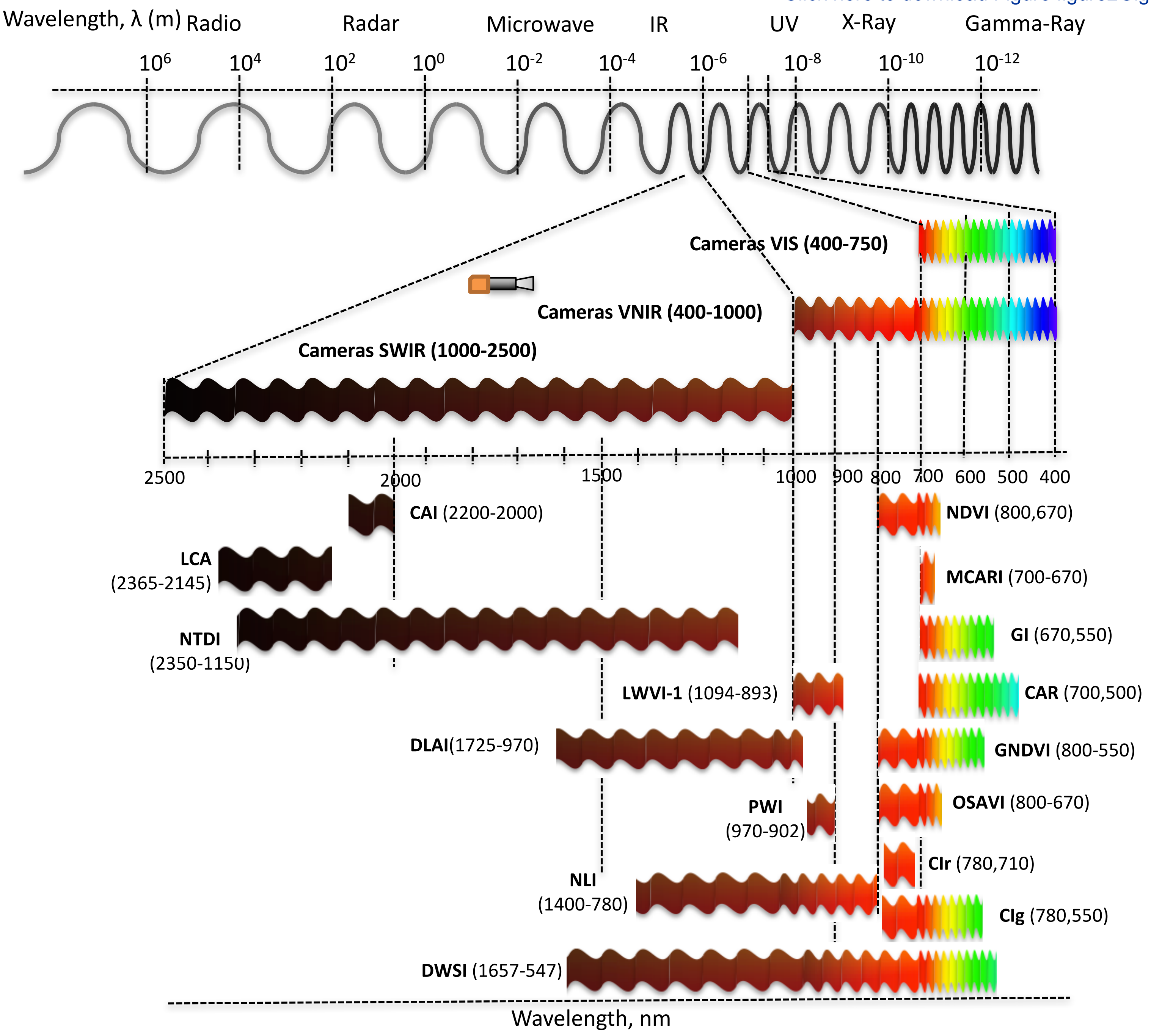

Supplement: GIGA-D-17-00043_Revision-2.pdf [file gix092_giga-d-17-00043_revision-2.pdf]
