# Supplementary material for: Plant phenomics: an overview of image acquisition technologies and image data analysis algorithms
Source: Gigascience. 2017 Oct 3;6(11):1–18. doi: 10.1093/gigascience/gix092 (PMC5737281; doi:10.1093/gigascience/gix092)
Supplement: Response-to-Reviewer-Comments_Original-Submission.pdf [file gix092_response-to-reviewer-comments_original-submission.pdf]

Author's Response To Reviewer Comments

Dear Dr. Nogoy, thank you very much for the speedy review and the high quality of the referees you chose. We believe that their input has improved our manuscript and we have taken their and your advice into account.
Please find enclosed a detailed response that can be also found in the manuscript. We have done a major rewriting to center it around two questions, image acquisition and data analysis. We have deleted a part we wrote about the different types of indexes used as it was informative but not really related to the major points.

As a result, we believe that all the referees requests have been met. Some via direct correction and many by rewriting to clarify the points. In those cases where rewriting deleted a request, we have written it down on the letter found below.

Please note that the new manuscript has all corrections and rewriting in red.

We hope the referees will find this version suitable for publication.

1. Reviewer reports:

Reviewer #1: # Review giga science 20170317

The manuscript from Perez-Sanz et al is a review of image acquisition technologies and image data analysis algorithms used in plant sciences.

While the manuscript provides a nice overview of the available techniques and algorithms, I feel that it is, at least in this form, not suited for publication. The text needs to be clarified in numerous places (see comments below). Also, I think it is, too technical and would fit better in a more specialised journal such as Plant Methods.

General comments:

1- The English should be improved

We have used professional English edition to improve the language.

2- The scope in the text is varying a lot between sections, which makes the reading hard. Sometimes the text provide very precise information about a given experiment (e.g. line 59), while it stays very vague in other places. I think the whole text should be homogenised for easier understanding.

We have done a major rewriting to address this issue

3- The manuscript given the impression to be willing to make an overview of whole the existing sensors / algorithms used for plant image analysis. My feeling is that this tack is inherently huge, since each image acquisition / analysis problem will call for a specific solution. A thorough review would be enormous. This is not the case f this manuscript, which gives more of an overview.

We have addressed this problem by giving the major advantages/drawbacks and technical characteristics of image acquisition devices and image analysis procedures.

Some specific comments, to give an idea of the modifications that should be made (I haven't done the whole document):

4- Line 19 (first line of the abstract!): No, phenomics is not the field of atomic phenotype acquisition technologies. It is the field of phenome analysis and is not, strictly speaking, linked to any specific technology. Phenomics can be done by hand, with a ruler.

We agree with the referee and we have changed the formulation of the abstract.

5- Line 30: NDVI is not defined
We have defined NDVI and other abbreviations throughout the manuscript
6- line 41: the sentence discuses roots techniques, but cites shoot-related article
We have corrected this part by rewriting. The references in the previous manuscript(9-12 now 11- 13 were correct and did refer to roots

7- line 41: what do you mean by "Analysis of direct imaging"?
We have changed the phrase as we refer to extraction of quantitative data from images (now line 42-43
8- line 44: I guess that author mean growing setups
We have corrected it (now line 46)
9- line 47-67: I am not sure to understand the aim of this paragraph. How does this fit with the rest of the text? I have the feeling it justifies to use of reporter lines, not the use of imaging setups...

We have rewritten part of the paragraph to explain why. In principle reporter genes, specifically Green Fluorescent Protein and Luciferase fostered the use of artificial vision systems early on. (now 49-76)

10- line 67: why is drawback in crops?

We have clarified this point. Now line 72-76

11- Monovision: can't the infrared and fluorescence imaging setup be classified here? They would fit the definition given in line 101-103.

Although there are IR cameras acquiring a single wavelength most are RGB-IR so we have included this in the multispectral cameras section. (see lines 194-211)

12- 113: "developed to quantify QTL's" ->
We have corrected to "developed to identify QTL's" Now line 146
13- 114: "large POPULATION of RIL's"

This part was rewritten
14- 115: what do you mean by "elite lines"?
This part was rewritten (line 148). An elite line is a genetic line useful for further breeding. Usually they have pyramided QTLs and or dominant alleles conferring superior traits sought after.
15- 123: isn't it a "DEPTH map"?
The mistake is corrected. Deep map is replaced by depth map. (Line 168)
16- 125: ToF is not defined
We have added a complete description of ToF devices-(line 228-249)
17- 134: why are stereo vision low throughput? Not sure it is true. Many plant phenotyping platform have a stereo vison imaging inside the imaging cabinet for 3D reconstruction -> high throughput

We have eliminated that text and added a paragraph with merits and drawbacks of 3-D systems. (Line 182-190)

Figure 1: What do the two arrows mean?

We have remade Figure 1 that describes the process of image acquisition and analysis

Reviewer #2: Due to the diversity of plant phenotyping techniques and different goals of plant research, it is a challenge to review and summarize major works enrolled in plenty of imaging techniques, image analysis pipeline, and image processing algorithms. The authors attempt to review some efforts of images acquisition and image processing, which is encouraged. However, the structure of review is confused, and massive fundamental knowledge of images analysis (read like a textbook of digital image processing) exists in this main text, which also lacks the references and the authors' own opinions. In addition, more applications of plant phenotyping should be cited in this review. More discussions and more comparisons with different image analysis should be summarized and added combined with the authors' suggestions, which can guide and benefit the readers.

1. Line 3: I wonder this review whether focus on plant phenomics, if yes, please change the title to plant phenomics.
We have changed the title as suggested

2. Line 30: please use the full name of "NDVI" and other abbreviations for the first time in this article.

We have modified all abbreviations and introduced first the name.
We have rewritten the complete part to make it easier to read, and deleted the part on different indexes. We have kept Figure 2 and on Table 2 different indexes can be found.

3. Line 33: please add reference for the "analyse plant growth and biomass".
We have added a reference (Myneni et al Nature 1997) (Line 34, reference [2].
4. Line 41: what dose "direct imaging" mean?
We have changed the phrase as we refer to extraction of quantitative data from images (Line 42-43)

5. Line 48-52: please add references for the "Historically, the first type of screenings was developed using the Luciferase reporter gene driven by a promoter" and "Upon mutagenesis of a parental line harbouring a regulatory region activated or repressed by a certain biological process or an environmental condition, new germplasm has been recovered".

We have increased this part and included more references
6. line 69-72: The authors paid plenty of words to introduce the development of screening techniques in the second paragraph of the Background. However, why the purpose of this review is lacking. Why review of image acquisition and image analysis is needed?
This is a very good point, we have made a statement about this, as most literature about image processing is found in books describing how to do them and not as reviews about what to use and why. (Line 78-88)

7. Line 81: TDI is a new sensor? Or it is a new imaging technique with CCD?
TDI is not a new sensor; it is a special imaging acquisition technology that can be implemented over CCD or CMOS imaging sensors to improve their features. Currently it is possible to find TDI cameras in the portfolios of the most important cameras manufactures. We have modified the paragraph and included new references (Lines 102-108).
8. Line 77-93: please add references and add author's own opinion, instead of some general knowledge.

We have added some perspective about trends in all the types of cameras we have described (see last paragraph of each of the devices.

9. Line 96: five groups?
We have increased them to 7. This is a good point as it gives a clearer picture.
10. Line 105: please use the full name of "SPICY" for the first time.
We have corrected this throughout the paper
11. Line 99: "mono vision" should be changed to "mono RGB vision".

We have done this correction (now line 130 and following parts)

12. Line 121-123: please add reference for the "Basically, and after locating a point in two mono vision systems, it is possible to compute the distance from the point to the system. Images produced are known as deep maps".
We have added references
13. Line 125: please use the full name of "ToF" for the first time
We have corrected this point.
14. Line 134: the drawback of stereo vision system is low throughput, however, the author cited a reference "high-throughput stereo-vision system" in line 130.

We have corrected this and clarified it (line 192-190)
15. Line 138-139: "usually between 2 and 10?" please add reference.
This range that classify the multispectral cameras is changing along the last decade as technology is improved. We have found different manufacturers with multispectral cameras between 3 until 25 bands. We have added a reference for a multispectral camera with 25 bands. But in months, new cameras will be in the market with increased capacities. (Line 194-202).

16. Line 156: the citing of "Figure 2" appeared earlier than Figure 1. Please check it carefully.

We have corrected this (line 222)
17. Line 160-163: please add references.
18. Line 167-169: please add references.
19. Line 173-177: please add references.

20. Line 178-181: More applications of plant phenotpying with LIDAR in recent years should be cited. Please discuss the disadvantage of the LIDAR.
21. Line 177: the end of the sentence lacks punctuation.
We have rewritten this whole part and included new references
22. Line 186: "14.000 nm" should be change to "14,000 nm". The image which obtained by thermographic camera should include a range of wavelength. Moreover, please add the reference.
We have added the reference. We have added fluorescence imaging with the corresponding ranges and references (line 297-322)

23. Line 196: "as a result of UV light excitation" is not rigorous, and please add the reference.
We have rewritten this part (see above line 397-322)
24. Line 75-203: more image acquisition techniques, such as x-ray CT, should be added. And the authors should summarize the merit and drawback of these imaging techniques.

25. Line 227-229: please add the reference of the "In fact, when information is measured as entropy, pre-processing causes a decrease in entropy". Or this is the author's own opinion.
We have rewritten this entire secction
26. Line 235-265: please introduce the procedures of image correction and images enhancement more concisely, and please add the reference.
We have rewritten this entire secction
27. Line 271-272: please add the references to the "Leaf Area Index (LAI), biomass, chlorophyll concentration, photosynthetic activity", respectively.

28. Line 287: please add the references to the "RDVI" and "MSR".
29. Line 294: what "NIR" and "VIS" represented?
30. Line 301: "EVI (enhanced vegetation index)" should be changed into "enhanced vegetation index (EVI)". Please check the similar mistake carefully in the main text.
31. Line 305: you should add the meaning of "RED" and "BLUE".
32. Line 267-312: the summarization of indexes in Table 1 is appreciated. But the "Vegetation indexes" part may not be appropriate for the "Image pre-processing" part, and this part is too redundant.
33. Line 320: 3D or 3-D.
34. Line 336-337: please add the references of the "1500-1590 nm" and "1390-1430 nm".
35. Line 355: Despite RGB and HSV colour space, other colour components such as ExG are also frequently used in plant detection. The authors should introduce more colour components.
36. Line 359-360: please add the references of the "hue can discriminate to detect chlorophyll".
37. Line 368: what is the meaning of "h(.)"?
38. Line 394: please add the references of the "Gaussian Mixture Model (GMM)". And what is the meaning of "I"?
39. Line 474: what are the meaning of "(892-934)" and "(281-245)"?
40. Line 476: 28 in SURF?
41. Line 487: please use the full name of "FAST" for the first time.
42. Line 442-517: The authors give too much detail about the features. Little was introduced about the application of these features in plant phenotyping.
43. Line 538-544: The authors should give some suggestion about when to select supervised/unsupervised techniques.
44. Line 545-547: I agree that the selection of ML algorithm require actual experimentation for optimal results. However, there are some general advices, the author should mention that and give some suggestions.

We have rewritten this part to make it more accessible. As a result, all the comments have been taken into account

45. According to the Figure 1, the author should review some popular algorithm or software of data analysis. And the structure of image analysis in the main text is confused, and the author should reorganize the review via the workflow of Figure 1.

We have remade Figure 1 to make it clearer and matched the review with the Figure.
We think that data analysis is a completely different topic. We have table 3 with popular software for image analysis.
