# Supplementary material for: Plant phenomics: an overview of image acquisition technologies and image data analysis algorithms
Source: Gigascience. 2017 Oct 3;6(11):1–18. doi: 10.1093/gigascience/gix092 (PMC5737281; doi:10.1093/gigascience/gix092)
Supplement: Response-to-Reviewer-Comments_Revision-1.pdf [file gix092_response-to-reviewer-comments_revision-1.pdf]

Author's Response To Reviewer Comments

Dear Dr. Nogoy,
Thanks very much for the opportunity to improve the manuscript. We have met all the requests. We wanted to point out that referee •2 requested a list of advantages and disadvantages for the different types of algorithms used in image analysis. We believe that, while that can be generalized for sensors, it is not the case in image analysis. We argue that the researchers need to test several methods to identify the best for their specific type of image, and forecasting the best outcome is not reasonable.
We have written this in the text, and as requested added yet another table.
We hope that the work will suffice for publication, waiting of course the veredict of the referees.

Bests
Marcos

Reviewer reports:
Reviewer #1:
I would like to thank the authors for the changes they made in the manuscript. I have the feeling it reads much better now and is more accessible to a broader audience.

I still have a couple of comments/suggestions (minor ones):

Line 49-line 76: I am still not sure to understand why the authors choose to focus this paragraph on the used of gene reporter, which is a very narrow example of the phenotyping today. I would personally remove this section.

We have removed it

Line 581: "The majority of the work has been made in indoor-setups ": I am really not sure about that... A lot of tools and methods have been developed recently to work outdoor and into the field.

We have rephrased it to have a less blunt statement

Reviewer #2: I am appreciated that the authors made big efforts to rewrite this review according to the reviewer's opinions. In recent years, many similar reviews related with Plant Phenomics had been published. And the authors stated that: "a detailed review on different type of data analysis is lacking. In this review, we cover the current and emerging methods of image acquisition and processing allowing image-based phenomics". This review should focus on image data analysis depending on different data type or sensors, which is lacking. Thus, two major issues should be improved and summarized more clearly in this review. Moreover, some previous comments are ignored by the authors or the related sentences had been removed, which should be declared.

We took into consideration all the previous comments, but as we made a major rewriting, they do not always appear as a word by word answer.

From the previous version:
“We have corrected this (line 222)
17. Line 160-163: please add references.
18. Line 167-169: please add references.
19. Line 173-177: please add references.

20. Line 178-181: More applications of plant phenotpying with LIDAR in recent years should be cited. Please discuss the disadvantage of the LIDAR.
21. Line 177: the end of the sentence lacks punctuation.
We have rewritten this whole part and included new references
22. Line 186: "14.000 nm" should be change to "14,000 nm". The image which obtained by thermographic camera should include a range of wavelength. Moreover, please add the reference.
We have added the reference. We have added fluorescence imaging with the corresponding ranges and references (line 297-322)

23. Line 196: "as a result of UV light excitation" is not rigorous, and please add the reference.
We have rewritten this part (see above line 397-322)
24. Line 75-203: more image acquisition techniques, such as x-ray CT, should be added. And the authors should summarize the merit and drawback of these imaging techniques.

25. Line 227-229: please add the reference of the "In fact, when information is measured as entropy, pre-processing causes a decrease in entropy". Or this is the author's own opinion.
We have rewritten this entire section
26. Line 235-265: please introduce the procedures of image correction and images enhancement more concisely, and please add the reference.
We have rewritten this entire section
27. Line 271-272: please add the references to the "Leaf Area Index (LAI), biomass, chlorophyll concentration, photosynthetic activity", respectively.
28. Line 287: please add the references to the "RDVI" and "MSR".
29. Line 294: what "NIR" and "VIS" represented?
30. Line 301: "EVI (enhanced vegetation index)" should be changed into "enhanced vegetation index (EVI)". Please check the similar mistake carefully in the main text.
31. Line 305: you should add the meaning of "RED" and "BLUE".
32. Line 267-312: the summarization of indexes in Table 1 is appreciated. But the "Vegetation indexes" part may not be appropriate for the "Image pre-processing" part, and this part is too redundant.
33. Line 320: 3D or 3-D.
34. Line 336-337: please add the references of the "1500-1590 nm" and "1390-1430 nm".
35. Line 355: Despite RGB and HSV colour space, other colour components such as ExG are also frequently used in plant detection. The authors should introduce more colour components.
36. Line 359-360: please add the references of the "hue can discriminate to detect chlorophyll".
37. Line 368: what is the meaning of "h(.)"?

38. Line 394: please add the references of the "Gaussian Mixture Model (GMM)". And what is the meaning of "I"?
39. Line 474: what are the meaning of "(892-934)" and "(281-245)"?
40. Line 476: 28 in SURF?
41. Line 487: please use the full name of "FAST" for the first time.
42. Line 442-517: The authors give too much detail about the features. Little was introduced about the application of these features in plant phenotyping.
43. Line 538-544: The authors should give some suggestion about when to select supervised/unsupervised techniques.
44. Line 545-547: I agree that the selection of ML algorithm require actual experimentation for optimal results. However, there are some general advices, the author should mention that and give some suggestions.

We have rewritten this part to make it more accessible. As a result, all the comments have been taken into account
“

As you can see, the major rewriting does take into account your ideas and suggestions.

1. The authors should summarize the imaging techniques in one table, which includes sensors, applications, advantages, disadvantages, and so on.

The authors could find a good example in Table 1 of the reference: "44. Fiorani F, Schurr U. Future scenarios for plant phenotyping. Annu. Rev. Plant Biol."

-We wrote a table as suggested. Please notice that the table is empty on the algorithms of machine learning and ToF. As of today there isn’t a single paper published applied to phenomics. The aforementioned table does describe advantages and disadvantages of sensors(Fiorani and Schurr). However, during image analysis, the advantages or disadvantages are a case by case situation. Researchers end up using the procedure giving better results for the specific image acquisition setup.

The complex combinations of background, reflected image, wavelength, sensors and the rest of elements that comprise image acquisition, makes it impossible to come with a single recipe for image analysis. The current review tries to give a number of methods for the different stages of image processing. But it will always be the researcher the one to test the different approaches to identify the combinations that give better results.

So it would be misleading to write for instance that a SIFT algorithm will have advantages over RGB2Grayscale for stereo vision (see new table 4), as it will depend on the image, background, type of plant, etc.

The fact that there are so many publications testing different methods and algorithms shows that

image analysis is very much a trial and error procedure till a defined pipeline is setup for a specific need. We think this message has to get through.

2. In image analysis section, the structure should be re-organized and more sub-sections should be added. For example in image pre-processing section, the review should provide different strategies according to different sensors or data types (such as CT raw data, hyperspectral raw data, RGB raw data, infrared raw data, and so on).
We disagree with this as once an image is taken, the downstream process does not depend on the type of sensor used. We made table 4 to show what has been done so far, as requested. But once you have a matrix of pixels, the rest should be common procedures.

We have included a comprehensive table (Table 4) has been included as requested

Line 574:
A summary of the complete pipeline of image analysis including sensors, preprocessing, segmentation procedures, feature extractions and machine learning algorithms can be found in Table 4.

I believe that different imaging data need different pre-processing strategies. The authors just reviewed many pre-processing methods, but why choose this method and how to apply is lacking.

-As we said before this is incorrect, an image is a matrix of pixels. Authors test several methods and report the one that gave the best results.

The authors stated that: "We cannot conclude that a single preprocessing method will outperform other methods". Thus, the better or common used preprocessing method to handle different images is needed and should be listed in one table, which can benefit broader readers. The similar suggestions of the section "image segmentation, feature extraction" should be considered to modify this review.

We have added on table 4 a list of the most common used procedures. How to apply the different methods is a matter of taking the raw data and using different procedures and see for EACH case what gives better results. As we wrote above, recommending one over the other would be misleading. Furthermore, the current list of methods will probably increase in the future as the number of labs doing image analysis has increased dramatically in the last years.
