# Supplementary material for: Plant phenomics: an overview of image acquisition technologies and image data analysis algorithms
Source: Gigascience. 2017 Oct 3;6(11):1–18. doi: 10.1093/gigascience/gix092 (PMC5737281; doi:10.1093/gigascience/gix092)
Supplement: Reviewer-1-Report-(Original-Submission).pdf [file gix092_reviewer-1-report-(original-submission).pdf]

Reviewer Report

Title: Plant phenomics: an overview of image acquisition technologies and image data analysis algorithms

Version: Original Submission **Date:** 3/20/2017

Reviewer name: Guillaume Lobet

Reviewer Comments to Author:

Review giga science 20170317The manuscript from Perez-Sanz et al is a review of image acquisition technologies and image data analysis algorithms used in plant sciences.While the manuscript provides a nice overview of the available techniques and algorithms, I feel that it is, at least in this form, not suited for publication. The text needs to be clarified in numerous places (see comments below). Also, I think it is, too technical and would fit better in a more specialised journal such as Plant Methods.General comments:-- The English should be improved- The scope in the text is varying a lot between sections, which makes the reading hard. Sometimes the text provide very precise information about a given experiment (e.g. line 59), while it stays very vague in other places. I think the whole text should be homogenised for easier understanding.-- The manuscript given the impression to be willing to make an overview of whole the existing sensors / algorithms used for plant image analysis. My feeling is that this tack is inherently huge, since each image acquisition / analysis problem will call for a specific solution. A thorough review would be enormous. This is not the case f this manuscript, which gives more of an overview.Some specific comments, to give an idea of the modifications that should be made (I haven't done the whole document):-- Line 19 (first line of the abstract!): No, phenomics is not the field of atomic phenotype acquisition technologies. It is the field of phenome analysis and is not, strictly speaking, linked to any specific technology. Phenomics can be done by hand, with a ruler.- Line 30: NDVI is not defined- line 41: the sentence discuses roots techniques, but cites shoot-related article- line 41: what do you mean by "Analysis of direct imaging"?- line 44: I guess that author mean growing setups- line 47-67: I am not sure to understand the aim of this paragraph. How does this fit with the rest of the text? I have the feeling it justifies to use of reporter lines, not the use of imaging setups...- line 67: why is drawback in crops?- Monovision: can't the infrared and fluorescence imaging setup be classified here? They would fit the definition given in line 101-103.- 113: "developed to quantify QTL's" -> "developed to identify QTL's"- 114: "large POPULATION of RIL's"- 115: what do you mean by "elite lines"?- 123: isn't it a "DEPTH map"?- 125: ToF is not defined- 134: why are stereo vision low throughput? Not sure it is true. Many plant phenotyping platform have a stereo vison imaging inside the imaging cabinet for 3D reconstruction -> high throughputFigure 1: What do the two arrows mean?

Level of Interest

Please indicate how interesting you found the manuscript: An article of limited interest

Quality of Written English

Please indicate the quality of language in the manuscript: Not suitable for publication unless extensively edited

Declaration of Competing Interests

Please complete a declaration of competing interests, considering the following questions:

- Have you in the past five years received reimbursements, fees, funding, or salary from an organisation that may in any way gain or lose financially from the publication of this manuscript, either now or in the future?
- Do you hold any stocks or shares in an organisation that may in any way gain or lose financially from the publication of this manuscript, either now or in the future?
- Do you hold or are you currently applying for any patents relating to the content of the manuscript?
- Have you received reimbursements, fees, funding, or salary from an organization that holds or has applied for patents relating to the content of the manuscript?
- Do you have any other financial competing interests?
- Do you have any non-financial competing interests in relation to this paper?

If you can answer no to all of the above, write 'I declare that I have no competing interests' below. If your reply is yes to any, please give details below.

I declare that I have no competing interests

I agree to the open peer review policy of the journal. I understand that my name will be included on my report to the authors and, if the manuscript is accepted for publication, my named report including any attachments I upload will be posted on the website along with the authors' responses. I agree for my report to be made available under an Open Access Creative Commons CC-BY license (http://creativecommons.org/licenses/by/4.0/). I understand that any comments which I do not wish to be included in my named report can be included as confidential comments to the editors, which will not be published.

I agree to the open peer review policy of the journal
