# Supplementary material for: Plant phenomics: an overview of image acquisition technologies and image data analysis algorithms
Source: Gigascience. 2017 Oct 3;6(11):1–18. doi: 10.1093/gigascience/gix092 (PMC5737281; doi:10.1093/gigascience/gix092)
Supplement: Reviewer-2-Report-(Original-Submission).pdf [file gix092_reviewer-2-report-(original-submission).pdf]

Reviewer Report

Title: Plant phenomics: an overview of image acquisition technologies and image data analysis algorithms

Version: Original Submission **Date:** 4/11/2017

Reviewer name: Wanneng Yang

Reviewer Comments to Author:

Due to the diversity of plant phenotyping techniques and different goals of plant research, it is a challenge to review and summarize major works enrolled in plenty of imaging techniques, image analysis pipeline, and image processing algorithms. The authors attempt to review some efforts of images acquisition and image processing, which is encouraged. However, the structure of review is confused, and massive fundamental knowledge of images analysis (read like a textbook of digital image processing) exists in this main text, which also lacks the references and the authors' own opinions. In addition, more applications of plant phenotyping should be cited in this review. More discussions and more comparisons with different image analysis should be summarized and added combined with the authors' suggestions, which can guide and benefit the readers.1. Line 3: I wonder this review whether focus on plant phenomics, if yes, please change the title to plant phenomics.2. Line 30: please use the full name of "NDVI" and other abbreviations for the first time in this article.3. Line 33: please add reference for the "analyse plant growth and biomass".4. Line 41: what dose "direct imaging" mean?5. Line 48-52: please add references for the "Historically, the first type of screenings was developed using the Luciferase reporter gene driven by a promoter" and "Upon mutagenesis of a parental line harbouring a regulatory region activated or repressed by a certain biological process or an environmental condition, new germplasm has been recovered".6. line 69-72: The authors paid plenty of words to introduce the development of screening techniques in the second paragraph of the Background. However, why the purpose of this review is lacking. Why review of image acquisition and image analysis is needed?7. Line 81: TDI is a new sensor? Or it is a new imaging technique with CCD?8. Line 77-93: please add references and add author's own opinion, instead of some general knowledge.9. Line 96: five groups?10. Line 105: please use the full name of "SPICY" for the first time.11. Line 99: "mono vision" should be changed to "mono RGB vision".12. Line 121-123: please add reference for the "Basically, and after locating a point in two mono vision systems, it is possible to compute the distance from the point to the system. Images produced are known as deep maps".13. Line 125: please use the full name of "ToF" for the first time.14. Line 134: the drawback of stereo vision system is low throughput, however, the author cited a reference "high-throughput stereo-vision system" in line 130.15. Line 138-139: "usually between 2 and 10?" please add reference.16. Line 156: the citing of "Figure 2" appeared earlier than Figure 1. Please check it carefully.17. Line 160-163: please add references.18. Line 167-169: please add references.19. Line 173-177: please add references.20. Line 178-181: More applications of plant phenotpying with LIDAR in recent years should be cited. Please discuss the disadvantage of the LIDAR.21. Line 177: the end of the sentence lacks punctuation.22. Line 186: "14.000 nm" should be change to "14,000 nm". The image which obtained by thermographic

camera should include a range of wavelength. Moreover, please add the reference.23. Line 196: "as a result of UV light excitation" is not rigorous, and please add the reference.24. Line 75-203: more image acquisition techniques, such as x-ray CT, should be added. And the authors should summarize the merit and drawback of these imaging techniques.25. Line 227-229: please add the reference of the "In fact, when information is measured as entropy, pre-processing causes a decrease in entropy". Or this is the author's own opinion.26. Line 235-265: please introduce the procedures of image correction and images enhancement more concisely, and please add the reference.27. Line 271-272: please add the references to the "Leaf Area Index (LAI), biomass, chlorophyll concentration, photosynthetic activity", respectively.28. Line 287: please add the references to the "RDVI" and "MSR".29. Line 294: what "NIR" and "VIS" represented?30. Line 301: "EVI (enhanced vegetation index)" should be changed into "enhanced vegetation index (EVI)". Please check the similar mistake carefully in the main text.31. Line 305: you should add the meaning of "RED" and "BLUE".32. Line 267-312: the summarization of indexes in Table 1 is appreciated. But the "Vegetation indexes" part may not be appropriate for the "Image pre-processing" part, and this part is too redundant.33. Line 320: 3D or 3-D.34. Line 336-337: please add the references of the "1500-1590 nm" and "1390-1430 nm".35. Line 355: Despite RGB and HSV colour space, other colour components such as ExG are also frequently used in plant detection. The authors should introduce more colour components.36. Line 359-360: please add the references of the "hue can discriminate to detect chlorophyll".37. Line 368: what is the meaning of "h(.)"?38. Line 394: please add the references of the "Gaussian Mixture Model (GMM)". And what is the meaning of "I"?39. Line 474: what are the meaning of "(892-934)" and "(281-245)"?40. Line 476: 28 in SURF?41. Line 487: please use the full name of "FAST" for the first time.42. Line 442-517: The authors give too much detail about the features. Little was introduced about the application of these features in plant phenotyping.43. Line 538-544: The authors should give some suggestion about when to select supervised/unsupervised techniques.44. Line 545-547: I agree that the selection of ML algorithm require actual experimentation for optimal results. However, there are some general advices, the author should mention that and give some suggestions.45. According to the Figure 1, the author should review some popular algorithm or software of data analysis. And the structure of image analysis in the main text is confused, and the author should reorganize the review via the workflow of Figure 1.

Level of Interest

Please indicate how interesting you found the manuscript: An article whose findings are important to those with closely related research interests

Quality of Written English

Please indicate the quality of language in the manuscript: Acceptable

Declaration of Competing Interests

Please complete a declaration of competing interests, considering the following questions:

- Have you in the past five years received reimbursements, fees, funding, or salary from an organisation that may in any way gain or lose financially from the publication of this manuscript, either now or in the future?
- Do you hold any stocks or shares in an organisation that may in any way gain or lose financially from the publication of this manuscript, either now or in the future?
- Do you hold or are you currently applying for any patents relating to the content of the manuscript?
- Have you received reimbursements, fees, funding, or salary from an organization that holds or has applied for patents relating to the content of the manuscript?
- Do you have any other financial competing interests?
- Do you have any non-financial competing interests in relation to this paper?

If you can answer no to all of the above, write 'I declare that I have no competing interests' below. If your reply is yes to any, please give details below.

I declare that I have no competing interests

I agree to the open peer review policy of the journal. I understand that my name will be included on my report to the authors and, if the manuscript is accepted for publication, my named report including any attachments I upload will be posted on the website along with the authors' responses. I agree for my report to be made available under an Open Access Creative Commons CC-BY license (http://creativecommons.org/licenses/by/4.0/). I understand that any comments which I do not wish to be included in my named report can be included as confidential comments to the editors, which will not be published.

I agree to the open peer review policy of the journal
