# Supplementary material for: Plant phenomics: an overview of image acquisition technologies and image data analysis algorithms
Source: Gigascience. 2017 Oct 3;6(11):1–18. doi: 10.1093/gigascience/gix092 (PMC5737281; doi:10.1093/gigascience/gix092)
Supplement: Reviewer-2-Report-(Revision-1).pdf [file gix092_reviewer-2-report-(revision-1).pdf]

Reviewer Report

Title: Plant phenomics: an overview of image acquisition technologies and image data analysis algorithms

Version: Revision 1 **Date:** 7/16/2017

Reviewer name: Wanneng Yang

Reviewer Comments to Author:

I am appreciated that the authors made big efforts to rewrite this review according to the reviewer's opinions. In recent years, many similar reviews related with Plant Phenomics had been published. And the authors stated that: "a detailed review on different type of data analysis is lacking. In this review, we cover the current and emerging methods of image acquisition and processing allowing image-based phenomics". This review should focus on image data analysis depending on different data type or sensors, which is lacking. Thus, two major issues should be improved and summarized more clearly in this review. Moreover, some previous comments are ignored by the authors or the related sentences had been removed, which should be declared.
1. The authors should summarize the imaging techniques in one table, which includes sensors, applications, advantages, disadvantages, and so on. The authors could find a good example in Table 1 of the reference: "44. Fiorani F, Schurr U. Future scenarios for plant phenotyping. Annu. Rev. Plant Biol."
2. In image analysis section, the structure should be re-organized and more sub-sections should be added. For example in image pre-processing section, the review should provide different strategies according to different sensors or data types (such as CT raw data, hyperspectral raw data, RGB raw data, infrared raw data, and so on). I believe that different imaging data need different pre-processing strategies. The authors just reviewed many pre-processing methods, but why choose this method and how to apply is lacking. The authors stated that: "We cannot conclude that a single preprocessing method will outperform other methods". Thus, the better or common used preprocessing method to handle different images is needed and should be listed in one table, which can benefit broader readers. The similar suggestions of the section "image segmentation, feature extraction" should be considered to modify this review.

Level of Interest

Please indicate how interesting you found the manuscript: An article whose findings are important to those with closely related research interests

Quality of Written English

Please indicate the quality of language in the manuscript: Acceptable

Declaration of Competing Interests

Please complete a declaration of competing interests, considering the following questions:

- Have you in the past five years received reimbursements, fees, funding, or salary from an organisation that may in any way gain or lose financially from the publication of this manuscript, either now or in the future?
- Do you hold any stocks or shares in an organisation that may in any way gain or lose financially from the publication of this manuscript, either now or in the future?
- Do you hold or are you currently applying for any patents relating to the content of the manuscript?
- Have you received reimbursements, fees, funding, or salary from an organization that holds or has applied for patents relating to the content of the manuscript?
- Do you have any other financial competing interests?
- Do you have any non-financial competing interests in relation to this paper?

If you can answer no to all of the above, write 'I declare that I have no competing interests' below. If your reply is yes to any, please give details below.

I declare that I have no competing interests

I agree to the open peer review policy of the journal. I understand that my name will be included on my report to the authors and, if the manuscript is accepted for publication, my named report including any attachments I upload will be posted on the website along with the authors' responses. I agree for my report to be made available under an Open Access Creative Commons CC-BY license (http://creativecommons.org/licenses/by/4.0/). I understand that any comments which I do not wish to be included in my named report can be included as confidential comments to the editors, which will not be published.

I agree to the open peer review policy of the journal
